# The ETS transcription factor ETV6 constrains the transcriptional activity of EWS–FLI to promote Ewing sarcoma

**DOI:** 10.1038/s41556-022-01059-8

**Published:** 2023-01-19

**Authors:** Diana Y. Lu, Jana M. Ellegast, Kenneth N. Ross, Clare F. Malone, Shan Lin, Nathaniel W. Mabe, Neekesh V. Dharia, Ashleigh Meyer, Amy Conway, Angela H. Su, Julia Selich-Anderson, Cenny Taslim, Andrea K. Byrum, Bo Kyung A. Seong, Biniam Adane, Nathanael S. Gray, Miguel N. Rivera, Stephen L. Lessnick, Kimberly Stegmaier

**Affiliations:** 1 Harvard/MIT MD-PhD Program, Harvard Medical School, Boston, MA USA; 2 Department of Pediatric Oncology, Dana-Farber Boston Children’s Cancer and Blood Disorders Center, Boston, MA USA; 3 The Broad Institute of MIT and Harvard, Cambridge, MA USA; 4 Center for Childhood Cancer and Blood Diseases, Abigail Wexner Research Institute at Nationwide Children’s Hospital, Columbus, OH USA; 5 Department of Cancer Biology, Dana-Farber Cancer Institute, Boston, MA USA; 6 Department of Biological Chemistry and Molecular Pharmacology, Harvard Medical School, Boston, MA USA; 7 Department of Pathology, Massachusetts General Hospital and Harvard Medical School, Boston, MA USA; 8 Center for Cancer Research, Massachusetts General Hospital and Harvard Medical School, Charlestown, MA USA; 9 Division of Pediatric Hematology, Oncology and BMT, The Ohio State University College of Medicine, Columbus, OH USA

**Keywords:** Sarcoma, Oncogenes, Cancer epigenetics

## Abstract

Transcription factors (TFs) are frequently mutated in cancer. Paediatric cancers exhibit few mutations genome-wide but frequently harbour sentinel mutations that affect TFs, which provides a context to precisely study the transcriptional circuits that support mutant TF-driven oncogenesis. A broadly relevant mechanism that has garnered intense focus involves the ability of mutant TFs to hijack wild-type lineage-specific TFs in self-reinforcing transcriptional circuits. However, it is not known whether this specific type of circuitry is equally crucial in all mutant TF-driven cancers. Here we describe an alternative yet central transcriptional mechanism that promotes Ewing sarcoma, wherein constraint, rather than reinforcement, of the activity of the fusion TF EWS–FLI supports cancer growth. We discover that ETV6 is a crucial TF dependency that is specific to this disease because it, counter-intuitively, represses the transcriptional output of EWS–FLI. This work discovers a previously undescribed transcriptional mechanism that promotes cancer.

## Abstract (web summary)

Independent but complementary studies from Vakoc and Stegmaier identify and characterize a role for ETV6 in counteracting the transcriptional activity of EWS–FLI during Ewing sarcoma development, which may be targeted for therapeutic benefits.

## Main

As fundamental drivers of cell-type-specific identity and function, aberrant TFs represent an important class of genetic dependencies across distinct cancer types^1^. Paediatric cancers exhibit few mutations genome-wide, but typically harbour sentinel mutations that alter TF proteins^2–5^. Mutant TFs can hijack wild-type lineage-specific TFs into self-reinforcing, feed-forward core regulatory circuits (CRCs)^6–14^. For example, MYCN in *MYCN-*amplified neuroblastoma and the PAX3–FOXO1 and PAX7–FOXO1 fusion proteins in alveolar rhabdomyosarcoma promote tumour growth by hijacking tumour-type-specific CRC TFs^15–19^. It is not known, however, to what extent distinct cancer types harbouring mutant TFs rely on this specific type of circuitry.

Ewing sarcoma, the second most common paediatric bone cancer, is defined by pathognomonic chromosomal translocations that fuse a gene member of the FET family of RNA-binding proteins with members of the ETS family of TFs^20,21^. In 85–90% of cases, a translocation fuses the *EWSR1* and *FLI1* genes to encode the EWS–FLI fusion protein. EWS–FLI proteins exhibit the neomorphic ability to pioneer de novo enhancers at microsatellites that contain tandem ETS 5′-GGAA-3′ motif repeats^22–31^ via multimerization and recruitment of chromatin-modifying complexes, which in turn lead to an altered gene expression programme^20,32^.

Efforts to establish key dependencies in Ewing sarcoma have prioritized the identification of specific gene targets of EWS–FLI. Studies have described cell-type-specific TFs that are activated by, and cooperate with, EWS–FLI to reinforce oncogenic programmes^23,24,32–39^, including in CRCs^40^. Unbiased and systematic approaches are needed, however, to reveal crucial disease mechanisms specific to Ewing sarcoma.

Here we describe the results of a genome-scale CRISPR–Cas9 screen revealing that the wild-type ETS TF ETS variant 6 (ETV6; also known as TEL) is a crucial Ewing-sarcoma-selective TF dependency. We validate this dependency in vitro and in vivo. In contrast to selective TF dependencies that reinforce the oncogenic programmes of mutant TFs in other cancer types, the repressive activity of ETV6 constrains *EWS*–*FLI* gene activation at 5′-GGAA-3′ repeat enhancers to promote Ewing sarcoma growth. We therefore discover a previously undescribed mechanism promoting cancer: competition on chromatin between an oncogenic fusion TF and a ‘restraining’ inhibitory TF.

## Results

### ETV6 is a selective TF dependency in Ewing sarcoma

**Fig. 1:**
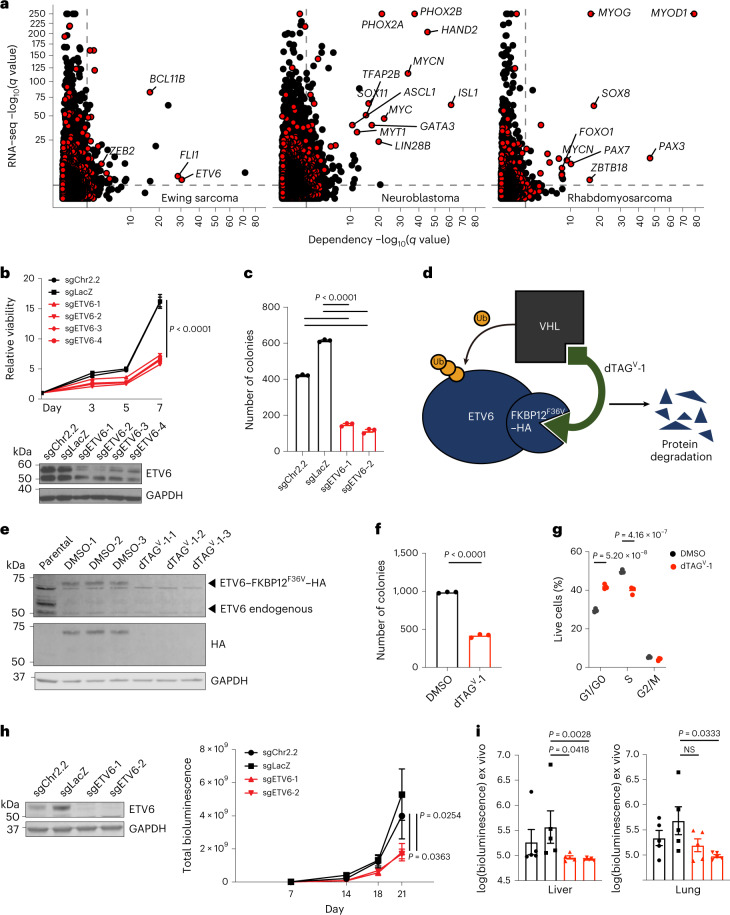
The repressive ETS TF ETV6 is a selective dependency in Ewing sarcoma cells. **a**, Scatter plot depicting 18,333 genes interrogated in the DepMap CRISPR–Cas9 screen. –log_10_(*q* value) of enrichment (*x* axis) measures the specificity of dependency for each tumour type^41^ (Ewing, *n* = 14; neuroblastoma, *n* = 20; rhabdomyosarcoma, *n* = 11). –log_10_(*q* value) of enrichment (*y* axis) measures the specificity of gene expression for each tumour type^74^ (Ewing, *n* = 20; neuroblastoma, *n* = 28; rhabdomyosarcoma, *n* = 18). TF genes^84^ are red and labelled if *x* > 8 (except *ZEB2* = 3.28). Dashed lines show –log_10_(0.05). **b**, Top: line graph depicting mean cell viability ± s.e.m. in A673 Ewing sarcoma cells transduced with CRISPR–Cas9 constructs targeting *ETV6* (sgETV6-1 to sgETV6-4) or control single guide RNAs (sgChr2.2 cutting; sgLacZ non-cutting) (*n* = 8 biological replicates, two-way analysis of variance (ANOVA), Dunnett’s multiple comparisons, *P* adjusted < 0.0001). Represents two independent experiments. Bottom: western blot shows ETV6 with GAPDH loading control. **c**, Bar plot showing mean ± s.e.m. number of A673 cell colonies in methylcellulose. ETV6 loss reduced the colony number (one-way ANOVA, *n* = 3 biological replicates, Sidak’s multiple comparisons, *P* adjusted < 0.0001). Represents two independent experiments. **d**, Schematic of the dTAG approach used to study ETV6. Ub, ubiquitin; VHL, von Hippel–Lindau. **e**, Western blot demonstrating ETV6–FKBP12^F36V^–HA protein degradation and endogenous *ETV6* knockout in A673 ETV6–dTAG cells treated with dTAG^V^-1 or DMSO for 6 h. Parental A673 lysates are shown on the left. Represents one experiment. **f**, Bar plot showing mean ± s.e.m. number of A673 ETV6–dTAG cell colonies in methylcellulose (*n* = 3 biological replicates, two-tailed *t*-test, *P* < 0.0001). Represents two independent experiments. **g**, Cell cycle analysis of A673 ETV6–dTAG cells treated for 72 h with DMSO or dTAG^V^-1 (*n* = 3 biological replicates, two-tailed *t*-test, Sidak’s multiple comparisons; G1/G0 phase, *P* adjusted = 5.20 × 10^−8^; S phase, *P* adjusted =4.16 × 10^−7^). Represents two independent experiments. **h**, Left: western blot of A673 cells implanted intramuscularly. Right: mean total body bioluminescence (±s.e.m.) (*n* = 5 mice per condition, biological replicates). ETV6 loss reduced tumour growth compared with sgChr2.2 (two-way ANOVA, Tukey’s multiple comparisons; sgETV6-1, *P* adjusted = 0.0363; sgETV6-2, *P* adjusted = 0.0254). **i**, Mean ± s.e.m. log(bioluminescence) measurements of ex vivo resected liver and lung (*n* = 5). sgLacZ livers exhibited greater bioluminescence than sgETV6-1 (Kruskal–Wallis test, Dunn’s multiple comparisons; *P* adjusted = 0.0418) or sgETV6-2 livers (*P* adjusted = 0.0028). sgLacZ lungs exhibited greater bioluminescence than sgETV6-2 (*P* adjusted = 0.0333) but not sgETV6-1 lungs (not significant (NS), *P* adjusted = 0.2763). Key is the same as **h**. Source data

**Extended Data Fig. 1:**
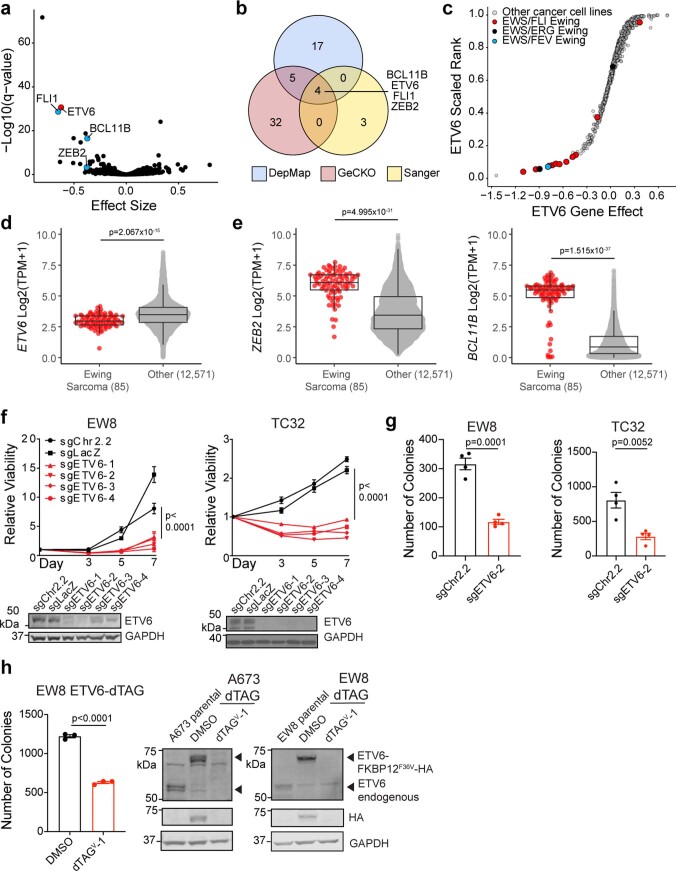
The repressive ETS transcription factor, ETV6, is a selective dependency in Ewing sarcoma cells. **a**. Volcano plot of genes in the DepMap screen in Ewing sarcoma cell lines (n = 14) compared to all other cell lines (n = 782). Effect size (x-axis) indicates the impact of gene deletion on growth. Log10(q-value) (y-axis) indicates specificity of dependency in Ewing sarcoma. Blue marks known selective TF dependencies. **b**. Venn diagram of Ewing sarcoma selective TF dependencies in the DepMap, GeCKO, and Sanger CRISPR/Cas9 screens. **c**. Scaled rank plot depicting all cell lines in DepMap. Gene effect (x-axis) measures *ETV6* deletion impact in each cell line. Ewing lines are enlarged and color-coded by specific EWS/ETS fusion (EWS/FLI n = 11, EWS/ERG n = 2, EWS/FEV n = 1). **d and e**. Expression (log2(TPM + 1); TPM, transcripts per million) of *ETV6* (**d**) and *BCL11B*, *ZEB2* (**e**) in primary tumors (Treehouse Childhood Cancer Initiative^37^, Ewing sarcoma n = 85; other n = 12,571). Points show the full range between maxima and minima. Boxes show values for the 25^th^ and 75^th^ percentiles; middle line shows median (50^th^ percentile). Whiskers extend no further than 1.5 times the inter-quartile range. Gene expression in Ewing sarcoma was different from other tumor types (Welch 2^−^sample T-test, *ETV6* p = 2.067 × 10^−15^, *ZEB2* p = 4.995 × 10^−31^, *BCL11B* p = 1.515 × 10^−37^). **f**. Line graphs depicting mean cell viability ±SEM in Ewing sarcoma cell lines (n = 8 biological replicates). *ETV6* knock-out cells exhibited lower viability than control (2^−^way ANOVA, Dunnett multiple comparisons EW8 p-adj<0.0001; TC32 p-adj<0.0001). Represents two independent experiments. Westerns show ETV6 and GAPDH loading control (kDa, kiloDaltons). **g**. Bar plots showing mean ±SEM number of methylcellulose cell colonies. *ETV6* knock-out samples formed fewer colonies (EW8 two-tailed t-test, n = 4 biological replicates, p = 0.0001; TC32 two-tailed t-test, n = 4 biological replicates, p = 0.0052). **h**. (Left) Bar plot showing mean ±SEM number of EW8 ETV6-dTAG cell colonies. dTAG^V^-1-treated cells formed fewer colonies than control (n = 3 biological replicates, two-tailed t-test, p < 0.0001). (Right) Western blot of dTAG cells shown here and in Fig. 1f. Source data

We recently reported a genome-scale CRISPR–Cas9 loss-of-function screen performed in paediatric cancer cell lines (Pediatric Cancer DepMap) that identified transcriptional activators as a strongly enriched class of selective dependencies (genetic vulnerabilities unique to a specific cancer type) in several paediatric cancer subtypes^41^. Among the most highly selective dependencies were activating CRC TFs in neuroblastoma and rhabdomyosarcoma (Fig. 1a and Supplementary Tables 1–3). TFs involved in CRCs co-opted by MYCN in neuroblastoma (PHOX2B, HAND2, ISL1 and GATA3)^15,16^ and the PAX3–FOXO1 and PAX7–FOXO1 fusion proteins in alveolar rhabdomyosarcoma (MYOD1, MYOG, SOX8 and MYCN)^17,18^ constituted the strongest dependencies specific to each tumour type. By contrast, selective TF gene dependencies in Ewing sarcoma were transcriptional repressors, including the known dependencies *BCL11B* and *ZEB2* (refs. 36, 37), which are activated by EWS–FLI, and the previously uncharacterized dependency *ETV6* (Fig. 1a and Extended Data Fig. 1a). These TFs also scored in independent screens (Extended Data Fig. 1b). *ETV6* is not recurrently mutated in Ewing sarcoma^42–44^, and *ETV6* dependency was not associated with a specific EWS–ETS fusion (that is, EWS–FLI or EWS–ERG) (Extended Data Fig. 1c). *ETV6* was not defined as a gene regulated by EWS–FLI in 18 of 19 gene sets (Supplementary Table 4) and did not exhibit a unique pattern of expression in cell lines (Fig. 1a) or in primary tumours^45^ (Extended Data Fig. 1d and Supplementary Table 5) of Ewing sarcoma. By contrast, CRC TFs in neuroblastoma and rhabdomyosarcoma exhibited tumour-type-specific expression (Fig. 1a), as did *BCL11B* and *ZEB2* in Ewing sarcoma^45^ (Extended Data Fig. 1e and Supplementary Table 5).

**Extended Data Fig. 2:**
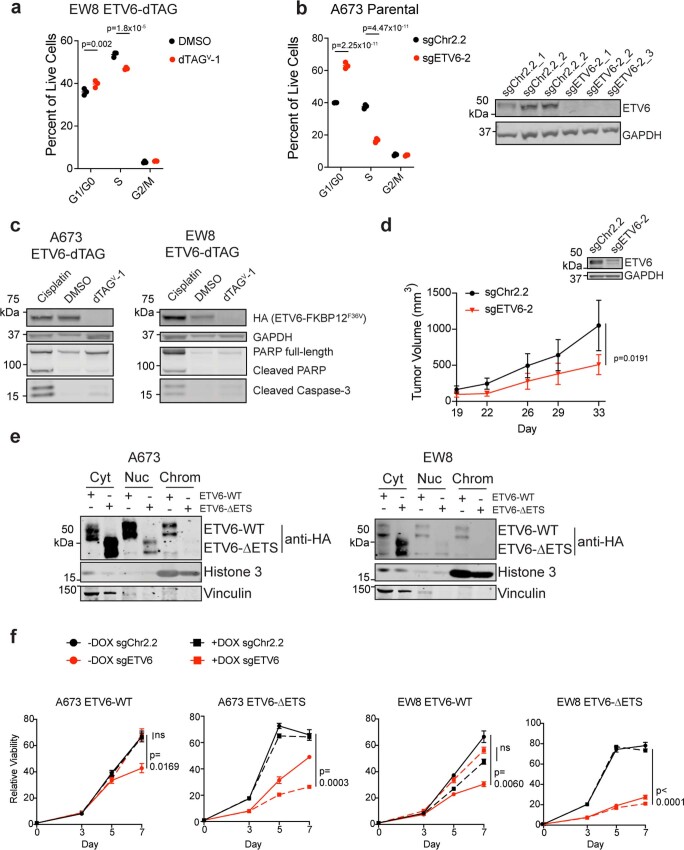
ETV6 promotes growth in Ewing sarcoma cells. **a**. Cell cycle analysis in EW8 ETV6-dTAG cells treated for 96 hours with DMSO or dTAG^V^-1 (n = 3 biological replicates, two-tailed t-test, Sidak’s multiple comparisons, G1/G0 p-adjust=0.002, S p-adj=1.8 × 10^−5^). **b**. (Left) Cell cycle analysis in A673 cells transduced with CRISPR/Cas9 constructs (n = 3 biological replicates, two-tailed t-test, Sidak’s multiple comparisons, G1/G0 p-adjust=2.25 × 10^−11^, S p-adj=4.47 × 10^−11^). (Right) Western blot of ETV6. **c**. Western blot of A673 and EW8 ETV6-dTAG cells treated with 1 µM cisplatin (as a positive control) for 24 hours or with DMSO or dTAG^V^-1 for 72 hours. Represents one independent experiment. **d**. (Left) Mean subcutaneous tumor volume in cubic millimeters ±SEM (n = 5, biological replicates) in mice implanted with CRISPR/Cas9-transduced TC32 cells (2-way ANOVA, p = 0.0191). (Right) Western blot showing ETV6. **e**. Western blots detecting exogenous expression of wild-type ETV6 protein (ETV6-WT) or mutant ETV6 harboring an ETS DNA binding domain deletion (ETV6-ΔETS) in cytosolic (Cyt), nuclear (Nuc), and chromatin (Chrom) subcellular fractions in A673 (left) and EW8 (right) cell lines. Vinculin and Histone 3 demonstrate the quality of fractionation. Represents one independent experiment. **f**. Line graphs depicting mean cell viability ±SEM (n = 4 biological replicates) in A673 and EW8 cells transduced with control (sgChr2.2, black) or *ETV6*-targeting (sgETV6, red) CRISPR/Cas9 constructs and expressing doxycycline-induced wild-type ETV6 (ETV6-WT) or ETS DNA binding domain-deleted ETV6 (ETV6-ΔETS). Cells were treated with either vehicle (circles, solid lines) or doxycycline (squares, dashed lines). Knockout of *ETV6* alone reduced cell viability (2-way ANOVA, Tukey’s multiple comparisons, A673 p-adj=0.0169; EW8 p-adj=0.0060). *ETV6* knockout in combination with wild-type *ETV6* exogenous expression did not alter cell viability (ns, A673 p-adj=0.9769; EW8 p-adj=0.0972), but *ETV6* knockout in combination with DBD-deleted *ETV6* expression reduced cell viability (A673 p-adj=0.0003; EW8 p-adj<0.0001). Source data

We validated an *ETV6* dependency in three cell lines of Ewing sarcoma, A673, EW8 and TC32, via CRISPR–Cas9 disruption. Loss of *ETV6* reduced cell growth in vitro (Fig. 1b and Extended Data Fig. 1f) and reduced anchorage-independent growth in methylcellulose (Fig. 1c and Extended Data Fig. 1g). We established a biochemical dTAG approach^46,47^ to perturb ETV6 abundance with precise temporal control and without eliciting acute DNA damage. FKBP12^F36V^-tagged proteins can be acutely degraded following exposure to the dTAG small-molecule dTAG^V^-1, which recruits the von Hippel–Lindau E3 ligase to ubiquitinate FKBP12^F36V^ (ref. 46). In the Ewing sarcoma cell lines A673 and EW8, we exogenously expressed ETV6 carboxy-terminally tagged with FKBP12^F36V^ and a human influenza haemagglutinin (HA) epitope (Fig. 1d). Simultaneously, we knocked out endogenous *ETV6* such that FKBP12^F36V^-tagged ETV6 constituted the dominant form of ETV6 protein. ETV6–FKBP12^F36V^ degradation reduced anchorage-independent growth (Fig. 1e,f and Extended Data Fig. 1h). Degradation of ETV6 (Fig. 1g and Extended Data Fig. 2a) as well as CRISPR–Cas9-mediated knockout of endogenous *ETV6* in parental A673 cells (Extended Data Fig. 2b) led to G1/G0 cell cycle arrest but did not induce apoptosis (Extended Data Fig. 2c and Supplementary Fig. 1).

In vivo, CRISPR–Cas9-mediated knockout of *ETV6* reduced the growth of subcutaneous TC32 tumours (Extended Data Fig. 2d). Using an orthotopic-like mouse model, in which A673 Ewing sarcoma cells implanted intramuscularly in the hindlimb are capable of metastasis^48^, we observed that ETV6 loss reduced primary tumour growth (Fig. 1h). ETV6 loss reduced metastasis to liver tissues (Fig. 1i, left), and lung tissues displayed the same trend in one out of two *ETV6* knockout conditions (Fig. 1i, right).

Next we asked whether the DNA-binding domain (DBD) of ETV6 was crucial to its function. We knocked out endogenous *ETV6* and exogenously expressed wild-type *ETV6* or mutant *ETV6* bearing a C-terminal DBD deletion, which precluded ETV6 binding to chromatin and partially impeded its nuclear localization. This result is consistent with the report that the nuclear localization signal of ETV6 protein lies in its C terminus^49^ (Extended Data Fig. 2e). Whereas wild-type *ETV6* expression rescued *ETV6* knockout, expression of the mutant *ETV6* did not (Extended Data Fig. 2f), which suggests that the specific activity of ETV6 on chromatin is crucial to its function in Ewing sarcoma.

### ETV6 and EWS–FLI co-occupy loci genome-wide

**Fig. 2:**
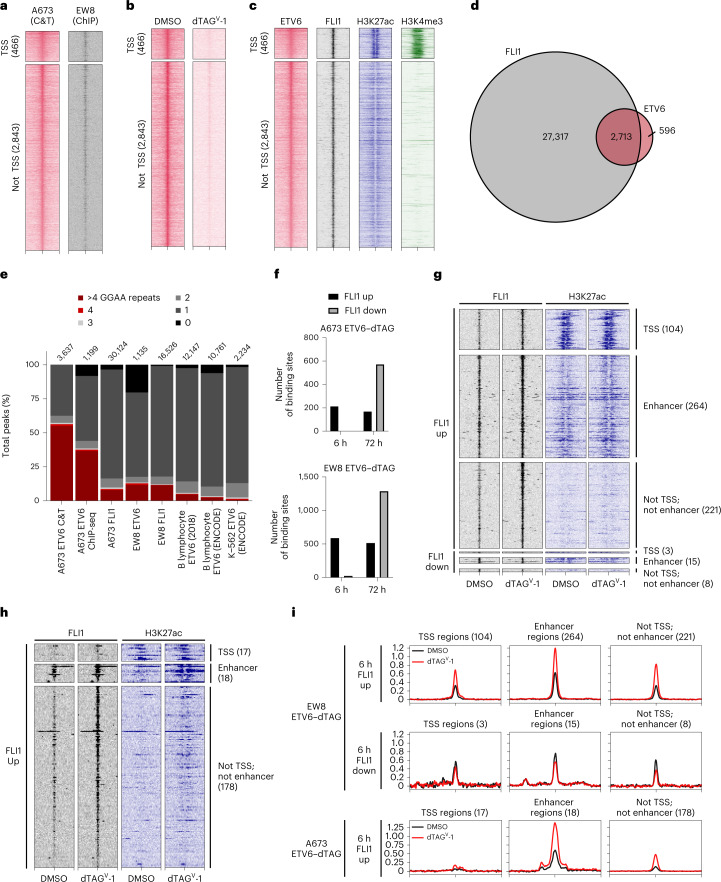
Acute loss of ETV6 leads to increased EWS–FLI binding. **a**–**c**, Heatmaps showing 3-kb windows centred at 3,309 consensus ETV6-binding sites, subplotted by overlap within 2.5 kb of transcription start sites (TSSs) and peaks ranked by maximum height. **a**, Left: CUT&Tag (C&T) of endogenous ETV6 in A673 parental cells. Right: anti-HA ChIP-seq of ETV6–FKBP12^F36V–^HA in EW8 ETV6–dTAG cells. **b**, Anti-HA CUT&Tag in A673 ETV6–dTAG cells treated with DMSO or dTAG^V^-1 for 24 h. **c**, Left to right: endogenous ETV6 CUT&Tag, EWS–FLI ChIP-seq, H3K27ac ChIP-seq and published H3K4me3 ChIP-seq^26^ in A673 cells. **d**, Venn diagram showing genomic locations of ETV6 consensus binding sites versus 30,030 EWS–FLI binding sites in A673 cells. **e**, Stacked column plot showing varying lengths of tandem 5′-GGAA-3′ motif repeats occurring at binding sites detected by (left to right): (1) endogenous ETV6 CUT&Tag in parental A673 cells; (2) ETV6–FKBP12^F36V^–HA ChIP-seq in A673 ETV6–dTAG cells; (3) EWS–FLI ChIP-seq in parental A673 cells; (4) ETV6–FKBP12^F36V^–HA ChIP-seq in EW8 ETV6–dTAG cells; (5) EWS–FLI ChIP-seq in parental EW8 cells; (6) and (7) endogenous ETV6 ChIP-seq in GM12878 B lymphocyte lymphoblastoid cells^51,52^; and (8) endogenous ETV6 ChIP-seq in K-562 chronic myelogenous leukaemia cells^52^. Number of binding sites in each dataset is shown. ETV6 bound to a higher percentage of >4 GGAA repeats in Ewing sarcoma compared to B lymphocyte ETV6 (2018) (A673 ETV6 C&T, *P* < 1 × 10^−300^; A673 ETV6 ChIP-seq, *P* = 5.41 × 10^−214^; and EW8 ETV6, *P* = 1.34 × 10^−18^; Fisher’s exact tests). **f**, Bar plots showing the number of genomic regions exhibiting significantly altered EWS–FLI binding at 6 or 72 h following ETV6 degradation identified by CSAW (CSAW using the edgeR generalized linear model; *P* < 0.05)^89^. FLI1 up sites exhibited increased EWS–FLI binding. FLI1 down sites exhibited decreased EWS–FLI binding. **g**,**h**, Heatmaps of EWS–FLI and H3K27ac ChIP-seq performed in EW8 ETV6–dTAG (**g**) and A673 ETV6–dTAG cells (**h**) at 6 h following DMSO or dTAG^V^-1 treatment. Loci exhibiting significantly altered EWS–FLI binding are subplotted by direction of change (up or down) and overlap with TSS, enhancer or neither. Enhancer locations were defined using H3K27ac ChIP-seq in parental EW8 (**g**) and A673 (**h**) cells. **i**, Metaplots of FLI1 binding at regions shown in **g** and **h**.

**Extended Data Fig. 3:**
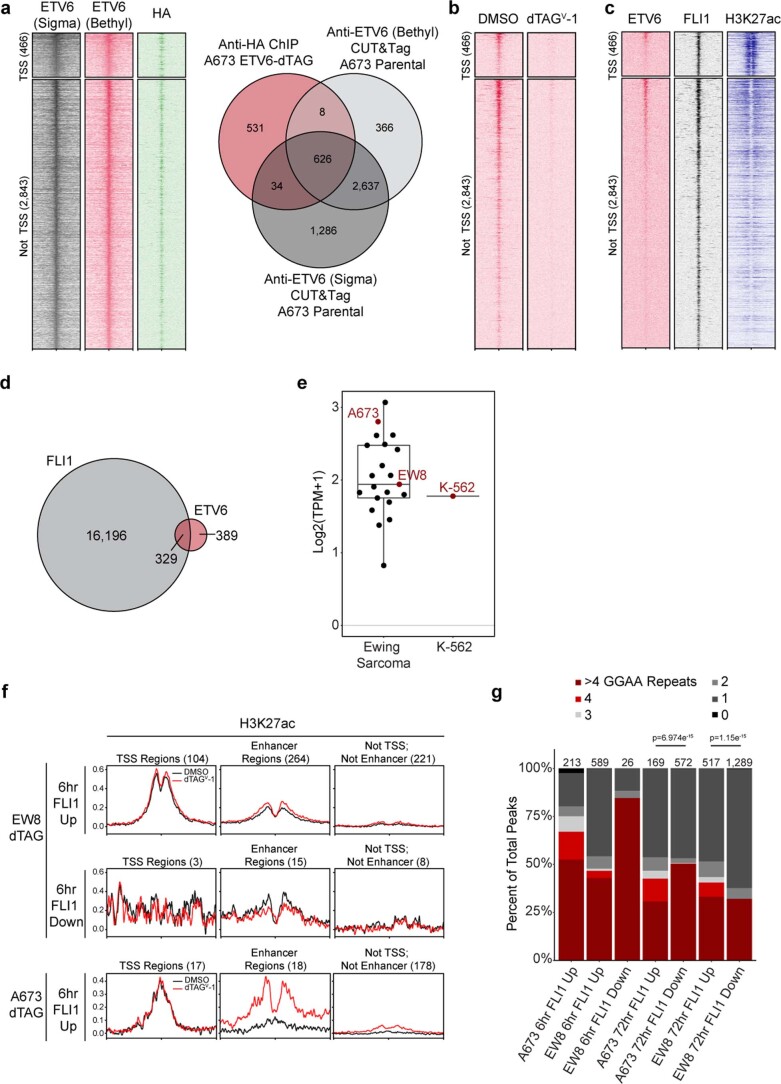
Acute loss of ETV6 leads to increased EWS/FLI binding. **a**. (Left) Heatmaps of endogenous ETV6 CUT&Tag in parental A673 cells using two commercial antibodies and anti-ETV6-FKBP12^F36V^-HA ChIP-seq in A673 ETV6-dTAG cells. Shown are ETV6 consensus binding sites sub-plotted by TSS overlap, ranked by height. (Right) Venn diagram showing that consensus binding sites were detected in at least 2 data sets. **b**. Heatmaps at consensus binding sites detected by anti-HA ChIP-seq in EW8 ETV6-dTAG cells at 24 hours DMSO or dTAG^V^-1 treatment. **c**. (Left to right): Anti-HA ChIP-seq in EW8 ETV6-dTAG cells, anti-FLI1 ChIP-seq in parental EW8 cells, anti-H3K27ac ChIP-seq in parental EW8 cells. **d**. Venn diagram showing overlap between 718 ETV6-FKBP12^F36V^-HA binding sites in EW8 ETV6-dTAG cells and 16,525 EWS/FLI binding sites in parental EW8 cells. **e**. Log2(TPM + 1) expression of *ETV6* in Ewing sarcoma cell lines (n = 21) and the K-562 leukemia cell line (n = 1) from CCLE. Specific cell lines are in red. Points show the full range between maxima and minima. Boxes show values for the 25^th^ and 75^th^ percentiles; middle line shows median (50^th^ percentile). Whiskers extend no further than 1.5 times the inter-quartile range. **f**. Metaplots of H3K27ac abundance at regions shown in Fig. 3g, h. **g**. Stacked column plot showing varying lengths of tandem 5′-GGAA-3′ motif repeats occurring at genomic regions exhibiting significantly altered EWS/FLI binding (CSAW using the edgeR generalized linear model; p < 0.05) in A673 or EW8 dTAG cells at 72 hours. The number of peaks in each data set is listed. No genomic regions lost EWS/FLI binding in A673 dTAG at 6 hours. At 72 hours, regions that gained EWS/FLI binding were enriched for repeats of 2, 3, and 4 compared to regions that lost EWS/FLI binding (Fisher exact test, A673 p = 6.974 × 10^−15^, EW8 p = 1.15 × 10^−15^).

ETV6 and EWS–FLI harbour the ETS family DBD, which recognizes consensus 5′-GGA(A/T)-3′ motifs. We therefore asked whether they co-localized on chromatin. We profiled endogenous ETV6 binding sites in parental A673 cells using cleavage under targets and tagmentation (CUT&Tag)^50^ and profiled ETV6–FKBP12^F36V^–HA binding sites in ETV6–dTAG cells using anti-HA chromatin immunoprecipitation with sequencing (ChIP-seq). These analyses defined a consensus list of ETV6-binding sites (Extended Data Fig. 3a and Fig. 2a). dTAG^V^-1 treatment reduced ETV6 abundance on chromatin in both dTAG models (Fig. 2b and Extended Data Fig. 3b). In parental Ewing sarcoma cells, we performed histone H3 lysine 27 acetylation (H3K27ac) ChIP-seq and analysed public histone H3 lysine 4 trimethylation (H3K4me3) ChIP-seq data^26^ to annotate ETV6-binding sites. The results showed that these sites occurred at active promoters and enhancers (Fig. 2c and Extended Data Fig. 3c). We performed ChIP-seq for EWS–FLI in A673 and EW8 parental cells by immunoprecipitating the C-terminal FLI1 domain. This is an accepted approach to identify EWS–FLI-binding sites because wild-type *FLI1* typically is not expressed in Ewing sarcoma cells^39,43^. EWS–FLI bound ubiquitously at ETV6-binding sites in both models (Fig. 2c and Extended Data Fig. 3c), although co-occupied binding sites constituted only a small proportion of total EWS–FLI-binding sites (Fig. 2d and Extended Data Fig. 3d). EWS–FLI pioneers closed chromatin at GGAA repeat microsatellites^27^, including at repeats of four or more^26^. ETV6 localized at these longer consecutive GGAA repeats at a higher frequency in Ewing sarcoma than in B lymphocytes or in K-562 leukaemia cells^51,52^, which express *ETV6* (*P* < 2.2 × 10^−16^) (Fig. 2e and Extended Data Fig. 3e).

### Loss of ETV6 increases EWS–FLI occupancy

We next asked whether loss of ETV6 alters EWS–FLI chromatin occupancy. We degraded ETV6 and profiled EWS–FLI binding by ChIP-seq at 6 and 72 h. At 6 h, in both dTAG models, significant alterations in EWS–FLI occupancy primarily constituted increases in binding (Fig. 2f). At 72 h, alterations were more dynamic, exhibiting both increases and decreases (Fig. 2f). We categorized loci by whether they gained or lost EWS–FLI binding at 6 h and whether they occurred at transcription start sites (TSSs) or at H3K27ac-defined enhancers (Fig. 2g,h). Regions that lost EWS–FLI binding did not change to as great a degree as regions that gained binding (Fig. 2i). Thus, the loss of ETV6 led acutely and predominantly to increased EWS–FLI binding, which provides support for the hypothesis that these TFs compete for binding. Additionally, ChIP-seq of H3K27ac at 6 h in both models (Fig. 2g,h) demonstrated a modest increase in H3K27ac abundance at enhancer regions that gained EWS–FLI binding (Extended Data Fig. 3f).

Differential EWS–FLI binding was highly dynamic at tandem 5′-GGAA-3′ repeats (Extended Data Fig. 3g). Notably, genomic regions that gained EWS–FLI binding were more likely to contain shorter tandem repeats of 2, 3 or 4 motifs compared with regions that lost EWS–FLI binding (*P* < *P* = 6.974 × 10^−15^). Consistent differences were not observed for single GGAA motifs or >4 GGAA repeats.

### ETV6 is a transcriptional repressor in Ewing sarcoma

**Fig. 3:**
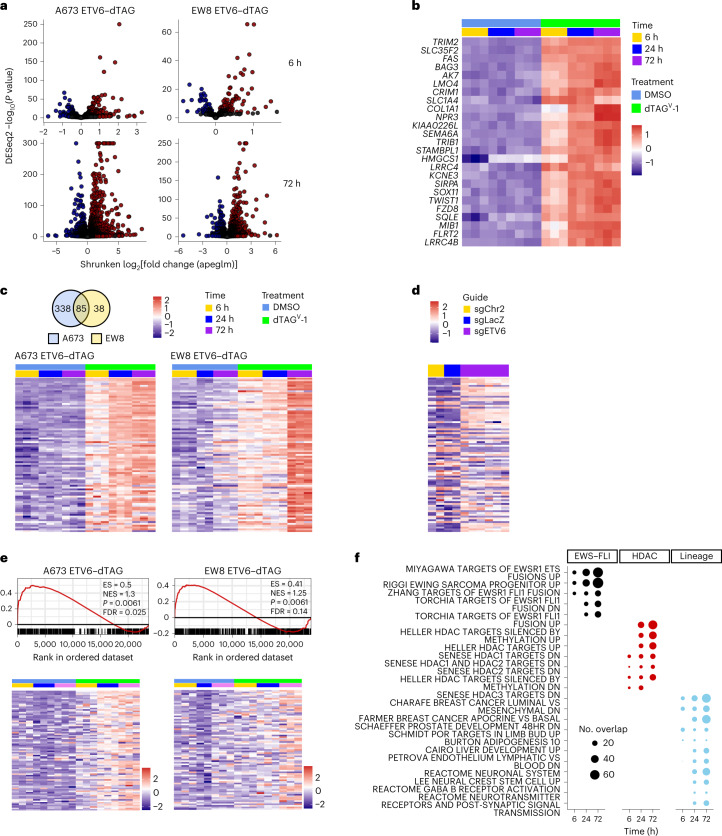
ETV6 is primarily a transcriptional repressor in Ewing sarcoma. **a**, RNA-seq volcano plot in A673 ETV6–dTAG and EW8 ETV6–dTAG cells treated with DMSO or dTAG^V^-1 for 6 and 72 h (*n* = 3 biological replicates). Red indicates genes upregulated in dTAG^V^-1-treated cells (DESeq2 Wald test Benjamini–Hochberg *P* adjusted < 0.05; A673: *n* = 423 at 6 h and 2,554 at 72 h; EW8: *n* = 123 at 6 h and 1,614 at 72 h). Blue indicates genes downregulated in dTAG^V^-1-treated cells (*P* adjusted < 0.05; A673: *n* = 221 at 6 h and 2,556 at 72 h; EW8: *n* = 67 at 6 h and 1,208 at 72 h). **b**, Row-normalized RNA-seq log_2_(transcripts per million (TPM) + 1) heatmap of the 25 most differentially repressed genes in A673 ETV6–dTAG cells, identified from 6-h data, ranked by *P* value (DESeq2 *P* adjusted < 0.05 and log_2_(fold change) > 1.5). **c**, Top left: Venn diagram of genes identified as ETV6-repressed in A673 ETV6–dTAG (423 genes) and EW8 ETV6–dTAG cells (123 genes) at 6 h (*P* adjusted < 0.05), identifying 85 common ETV6-repressed genes. Bottom: row-normalized RNA-seq log_2_(TPM + 1) heatmap of 85 common ETV6-repressed genes in A673 ETV6–dTAG and EW8 ETV6–dTAG cells. **d**. Row-normalized log_2_(TPM + 1) RNA-seq heatmap of 85 ETV6-repressed genes, ranked as shown in **c**, in parental A673 cells transduced with CRISPR–Cas9 vectors, identifying 53 ETV6-repressed genes using this approach (one-sided hypergeometric test, *P* = 2.66 × 10^−20^). **e**, Top: gene set enrichment analysis (GSEA) plots of ETV6-bound genes enriched in ETV6-regulated genes in A673 ETV6–dTAG and EW8 ETV6–dTAG cells. ETV6-bound genes were defined by CUT&Tag and ChIP-seq in A673 cells and by ChIP-seq in EW8 cells. ETV6-regulated genes were defined by RNA-seq at 24 h. Bottom: RNA-seq heatmaps of ETV6-repressed core enrichment genes. ES, enrichment score; FDR, false discovery rate; NES, normalized enrichment score. Colour coding for time and treatment is the same as in **c**. **f**, Combined enrichment plot of MSigDB c2 pathways significantly enriched in ETV6-repressed genes defined by RNA-seq at 24 h common to both models (hypergeometric enrichment test, *P* < 0.05). Gene sets are ranked by significance. Dot size indicates the number of genes in the overlap between the gene set and common ETV6-repressed genes at 6, 24 and 72 h (85, 251 and 832 genes, respectively). Missing dots indicate non-significance. ‘EWS–FLI’, ‘HDAC’ and ‘Lineage’ gene sets characterize genes regulated by EWS–FLI, genes regulated by histone deacetylase enzymes, and genes underlying tissue-specific development or function, respectively.

**Extended Data Fig. 4:**
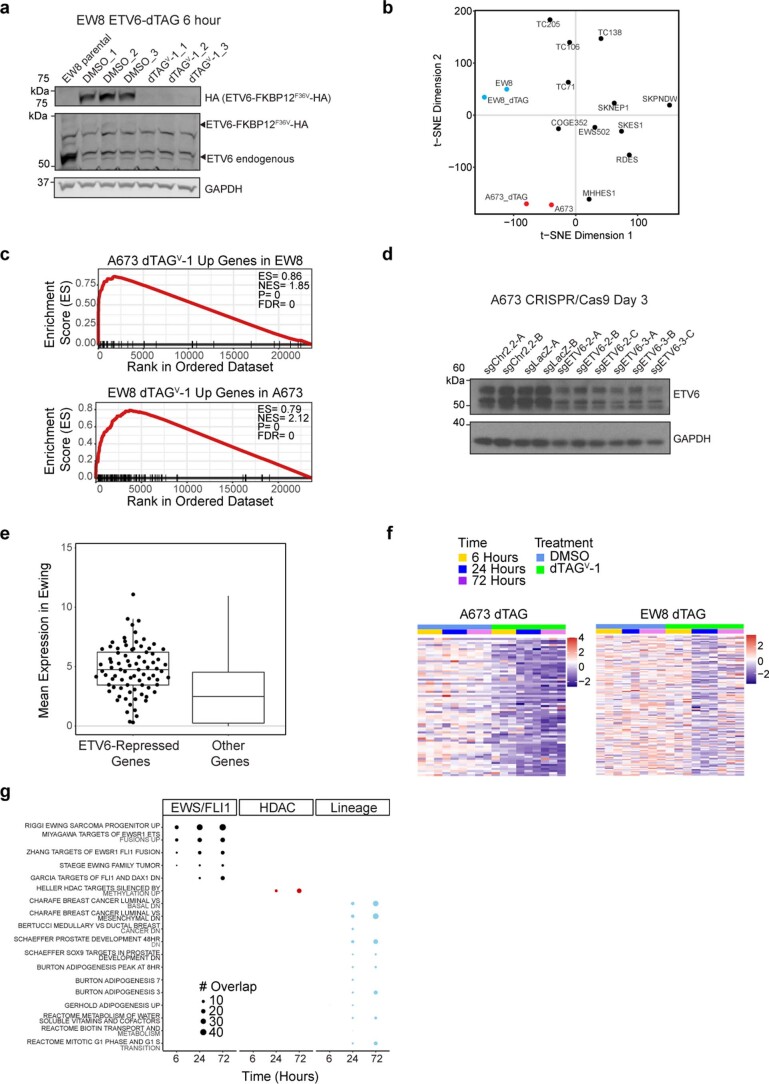
ETV6 is primarily a transcriptional repressor in Ewing sarcoma. **a**. Western blot of EW8 ETV6-dTAG cells treated for 6 hours with DMSO or dTAG^V^-1. Represents one experiment. **b**. t-SNE of RNA-seq expression in parental Ewing sarcoma cell lines from the Cancer Cell Line Encyclopedia (CCLE)^66^ and in A673 ETV6-dTAG and EW8 ETV6-dTAG cells treated for 6 hours with DMSO. EW8 parental and EW8 ETV6-dTAG cell samples are blue. A673 parental and A673 ETV6-dTAG cell samples are red. **c**. Gene set enrichment analysis (GSEA) of ETV6-repressed genes identified at 6 hours in A673 ETV6-dTAG compared to EW8 ETV6-dTAG RNA-seq data at 6 hours (top) and vice versa (bottom). **d**. Western blot of A673 parental cells transduced with sgChr2.2, sgLacZ, and sgETV6 CRISPR/Cas9 constructs. Represents one independent experiment. **e**. RNA-seq mean Log2(TPM + 1) for 81 of 85 ETV6-repressed genes, compared to all other genes, across Ewing sarcoma cell lines in CCLE^66^. Boxes show the values for the 25^th^ and 75^th^ percentiles; middle line shows the median (50^th^ percentile). Whiskers extend up from the 75^th^ percentile and down from the 25^th^ percentile, no further than 1.5*IQR (where IQR is the inter−quartile range, or distance between the 25^th^ and 75^th^ percentiles). Points show the full range between maxima and minima. **f**. RNA-seq heatmap of ETV6-activated core enrichment genes, identified by GSEA described in Fig. 3e, in A673 (68 genes) and EW8 dTAG cells (126 genes). **g**. Combined enrichment plot of MSigDB c2 pathways enriched in ETV6-activated genes in both dTAG models (24-hour RNA-seq, hypergeometric enrichment test; p < 0.05). Gene sets are ranked by significance; missing dots indicate insignificance. Dot size indicates the number of ETV6-activated genes at 6, 24, and 72 hours also in the gene set (33, 130, and 543 genes, respectively). ‘EWS/FLI’, ‘HDAC’, and ‘Lineage’ gene sets characterize EWS/FLI-regulated genes, histone deacetylase enzyme-regulated genes, and genes exhibiting tissue-specific expression, respectively.

We next characterized genes regulated by ETV6, a reported transcriptional repressor^53–57^. We performed RNA sequencing (RNA-seq) in both dTAG models at 6, 24 and 72 h following treatment with dimethylsulfoxide (DMSO) or dTAG^V^-1 (Fig. 1e and Extended Data Fig. 4a). Globally, the expression profiles of each of the engineered dTAG cell lines approximated that of their corresponding parental cell lines (Extended Data Fig. 4b). At 6 h, the majority of differentially expressed genes were upregulated, which suggests that ETV6 acts predominantly as a transcriptional repressor in Ewing sarcoma (Fig. 3a). Strongly ETV6-repressed genes increased in expression over time following ETV6 degradation (Fig. 3b). We observed concordance in regulated genes between dTAG models (Extended Data Fig. 4c) and identified a common set of 85 ETV6-repressed genes (Fig. 3c and Supplementary Table 6). We performed RNA-seq on parental A673 cells transduced with *ETV6* CRISPR knockout (Fig. 3d and Extended Data Fig. 4d). The results showed that most of the 85 genes were also repressed by endogenous levels of wild-type ETV6 (*P* = 2.66 × 10^−20^). Consistent with the localization of ETV6 at active promoters and enhancers, ETV6-repressed genes were expressed and not completely silenced (Extended Data Fig. 4e). Additionally, ETV6-binding sites were enriched in ETV6-regulated genes (Fig. 3e and Extended Data Fig. 4f).

ETV6 is a master TF implicated in the normal development of neural and mesenchymal lineages^58,59^. Developmental lineage-specific gene sets were enriched in ETV6-repressed genes (Fig. 3f and Supplementary Tables 6–11) and in ETV6-activated genes (Extended Data Fig. 4g and Supplementary Tables 12–17). ETV6-repressed genes, but not activated genes, were strongly enriched for genes regulated by histone deacetylases (HDACs), which may reflect the ability of ETV6 to recruit HDACs^54,60–63^. We also observed strong enrichment of EWS–FLI-regulated genes in ETV6-regulated genes (Fig. 3f), consistent with their co-localization on chromatin.

### Loss of ETV6 alters gene expression and the chromatin state

**Fig. 4:**
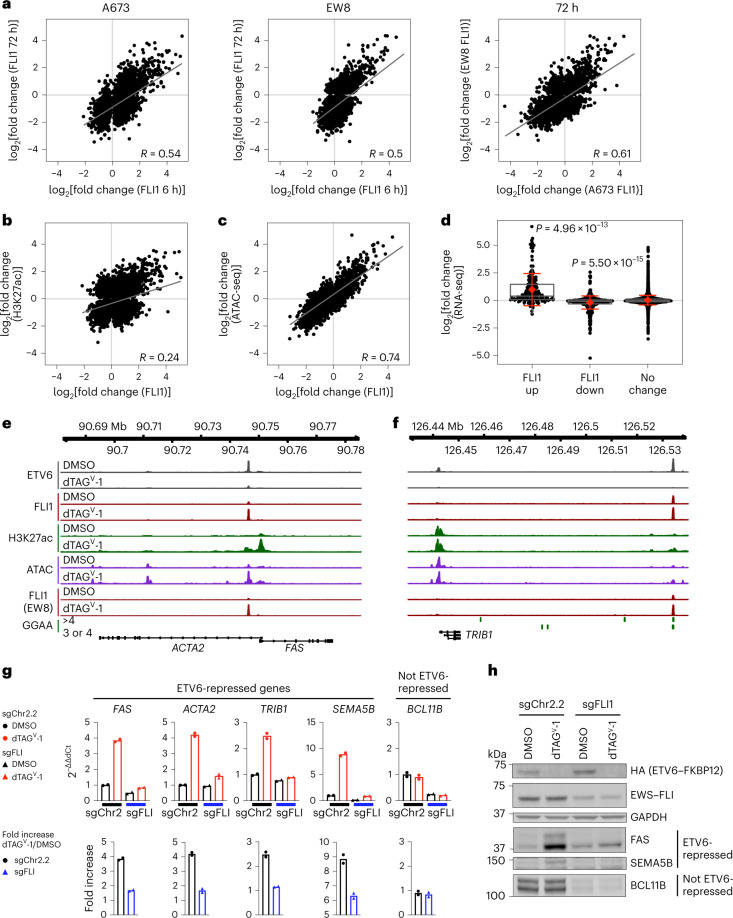
ETV6 constrains EWS–FLI-induced gene expression. **a**–**c**, Lines indicate Pearson correlation; Pearson correlation value (*R*) is shown. **a**, Left and middle: scatter plots of log_2_(fold change) in EWS–FLI binding at 6 and 72 h following DMSO or dTAG^V^-1 treatment in A673 and EW8 ETV6-dTAG cells (*n* = 2 biological replicates). Right: scatter plot comparing models. **b**, Scatter plot comparing log_2_(fold change) in EWS–FLI binding to H3K27ac abundance detected by 6 h ChIP-seq in A673 ETV6–dTAG cells (*n* = 2 biological replicates). **c**, Scatter plot comparing log_2_(fold change) in EWS–FLI binding in assay of transposase accessible chromatin sequencing (ATAC-seq) experiments (*n* = 3 biological replicates) at 72 h in A673 ETV6–dTAG cells. **d**, Plot comparing genes mapped from altered EWS–FLI binding sites (CSAW, *n* = 2 biological replicates) to log_2_(fold change) in expression measured by RNA-seq in A673 ETV6–dTAG cells (*n* = 3 biological replicates) at 72 h. Grey boxes indicate median and first and third quartiles. Red diamond and error bars indicate mean expression ± s.d. (FLI1 up, *n* = 148, mean = 0.98; FLI1 down, *n* = 542, mean = –0.19; no change, *n* = 4,585, mean = 0.028). *P* values calculated using paired *t*-test, Benjamini–Hochberg corrections. **e**,**f**, Gviz-generated views of the *FAS–ACTA2* (**e**) and *TRIB1* (**f**) loci. ETV6 tracks show CUT&Tag of ETV6–FKBP12^F36V^–HA in A673 ETV6–dTAG cells at 24 h. FLI1 tracks show ChIP-seq for EWS–FLI performed at 6 h, H3K27ac tracks show ChIP-seq for H3K27ac at 6 h and ATAC tracks show ATAC-seq at 72 h. FLI1 (EW8) tracks show ChIP-seq for EWS–FLI at 6 h in EW8 ETV6–dTAG cells. GGAA tracks indicate locations of tandem GGAA motif repeats. **g**, Top: bar plots showing qPCR in A673 ETV6–dTAG cells transduced with CRISPR–Cas9 constructs targeting control (sgChr2.2) or *EWS–FLI* (sgFLI) and treated for 24 h with DMSO (black) or dTAG^V^-1 (red). Bars indicate mean 2^−∆∆Ct^ of *n* = 2 biological duplicates, each representing the mean of technical triplicates. **h**, Western blot A673 ETV6–dTAG cells shown in **g** treated with DMSO or dTAG^V^-1 for 96 h. Source data

**Extended Data Fig. 5:**
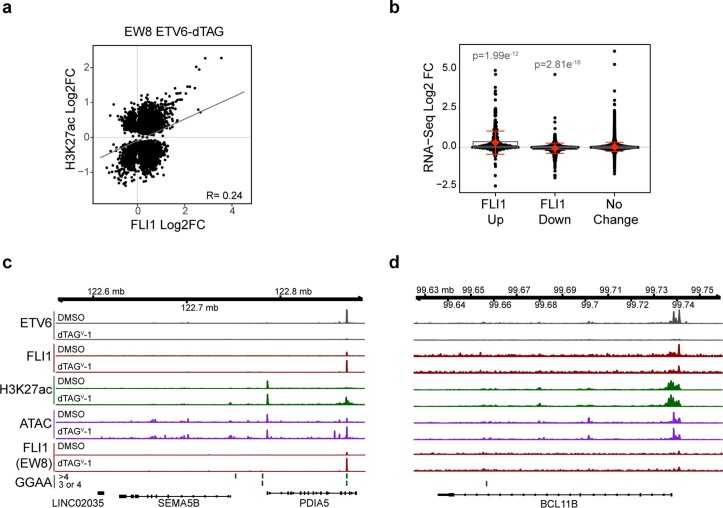
ETV6 constrains EWS/FLI-induced gene expression. **a**. Scatter plot comparing log2 fold-change in EWS/FLI binding to log2 fold-change in H3K27ac abundance detected by ChIP-seq at 6 hours in EW8 ETV6-dTAG cells (n = 2 biological replicates). Pearson correlation value (R) is shown. **b**. Plot comparing genes mapped from significantly altered EWS/FLI binding sites (n = 2 biological replicates) to Log2 Fold-change in expression measured by RNA-seq in EW8 ETV6-dTAG cells (n = 3 biological replicates) at 72 hours. Gray boxes indicate median and first and third quartiles. Red diamond and error bars indicate mean expression ±standard deviation (FLI1 Up n = 404 mean=0.3; FLI1 Down n = 1,042 mean = -0.062; No Change n = 8,252 mean=0.033). FLI1 Up vs. No Change p = 1.99e-12, FLI1 Down vs. No Change p = 2.81e-18, paired t-test, Benjamini-Hochberg corrections. **c and d**. Gviz-generated views of the *SEMA5B* (**c**) and *BCL11B* (**d**) loci. ETV6 tracks show CUT&Tag of ETV6-FKBP12^F36V^-HA in A673 ETV6-dTAG cells at 24 hours. FLI1 tracks show ChIP-seq for EWS/FLI performed at 6 hours, H3K27ac tracks show ChIP-seq for H3K27ac at 6 hours, and ATAC tracks show ATAC-seq at 72 hours. FLI1 (EW8) tracks show ChIP-seq for EWS/FLI at 6 hours in EW8 ETV6-dTAG cells. GGAA tracks indicate locations of tandem GGAA motif repeats.

We next sought to associate locus-specific alterations in chromatin with differential gene expression after ETV6 degradation. Alterations in EWS–FLI binding at individual loci at 6 h were sustained at 72 h, and these alterations correlated between dTAG models (Fig. 4a). Consistently, the greatest degree of differential EWS–FLI binding was exhibited by loci that gained EWS–FLI binding; loci that lost EWS–FLI binding exhibited smaller changes in magnitude (Fig. 4a). This pattern was paralleled by alterations in H3K27ac abundance at EWS–FLI-binding sites (Fig. 4b and Extended Data Fig. 5a) and by alterations in chromatin accessibility (Fig. 4c). We assigned EWS–FLI-binding sites to nearby genes and examined their expression following ETV6 loss (Fig. 4d). Genes that gained or lost EWS–FLI binding exhibited significantly increased or decreased expression, respectively (*P* < 1 × 10^−10^), with genes in the former category exhibiting the greatest degree of change on average (Fig. 4d and Extended Data Fig. 5b). Thus, the most profound consequences of ETV6 loss are increased EWS–FLI binding, opening of chromatin and increased gene expression.

### Increased EWS–FLI occupancy upregulates gene expression

We knocked out *EWS–FLI* in A673 ETV6–dTAG cells to evaluate whether loss of EWS–FLI rescues gene expression changes with ETV6 loss. *FAS*, *ACTA2*, *TRIB1* and *SEMA5B* were identified as ETV6-repressed genes that exhibit increased EWS–FLI binding, H3K27ac and chromatin accessibility at ETV6-vacated sites, some of which occurred at GGAA repeats (Fig. 4e,f and Extended Data Fig. 5c). We compared these genes to *BCL11B* because it is activated by EWS–FLI but is not repressed by ETV6 and does not exhibit altered EWS–FLI binding acutely following ETV6 loss (Extended Data Fig. 5d). Quantitative PCR (qPCR) demonstrated that degradation of ETV6 led to upregulation of ETV6-repressed genes but not *BCL11B* (Fig. 4g, top plots). *EWS–FLI* knockout significantly reduced the upregulation of ETV6-repressed genes (Fig. 4g, bottom plots). Immunoblotting validated that the attenuation of mRNA upregulation also affected protein levels (Fig. 4h). Thus, ETV6 and EWS–FLI antagonistically regulate *FAS*, *ACTA2*, *TRIB1* and *SEMA5B* expression.

### ETV6 functions similarly in clinically relevant Ewing sarcoma models

**Fig. 5:**
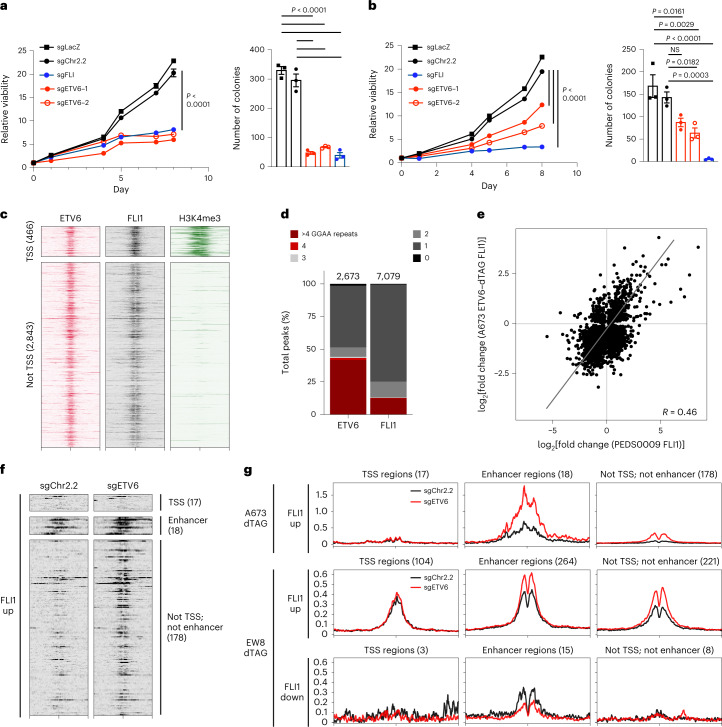
ETV6 competes with EWS–FLI for binding in clinically relevant Ewing sarcoma models. **a**,**b**, Cell growth in the newly derived Ewing sarcoma cell lines PEDS0009 (**a**) and PEDS0010 (**b**) following CRISPR–Cas9 knockout of *ETV6* (red) or *EWS–FLI* (blue) compared to sgChr2.2 and sgLacZ controls (black). Line graphs show mean cell viability ± s.e.m. (*n* = 6 biological replicates); knockout of *ETV6* and *EWS–FLI* reduced viability in both lines compared to sgChr2.2 (two-way ANOVA, Tukey’s multiple comparisons, *P* adjusted < 0.0001). Bar plots show mean cell colony number ± s.e.m. (*n* = 3 biological replicates) in methylcellulose; *ETV6* and *EWS–FLI* knockout reduced colony number in PEDS0009 cells (one-way ANOVA, Tukey’s multiple comparisons, *P* adjusted < 0.0001 for all comparisons indicated) and PEDS0010 cells (sgLacZ versus sgETV6-1, *P* adjusted = 0.0161, sgETV6-2, *P* adjusted = 0.0029, sgFLI, *P* < 0.0001; sgChr2.2 versus sgETV6-1, NS *P* adjusted = 0.1120, sgETV6-2, *P* adjusted = 0.0182, sgFLI, *P* adjusted = 0.0003). **c**, Heatmaps showing 3-kb windows centred at 3,309 consensus ETV6 binding sites, subplotted by overlap within 2.5 kb of TSSs. PEDS0009 cells were transduced with sgChr2.2 control CRISPR–Cas9 constructs and profiled by CUT&Tag to detect endogenous ETV6 (left) and CUT and release using nuclease (CUT&RUN) to detect EWS–FLI (middle) and the histone mark H3K4me3 (right). **d**, Stacked column plot showing varying lengths of tandem 5′-GGAA-3′ motif repeats occurring at ETV6 (left) and EWS–FLI (right) binding sites in PEDS0009 cells. **e**, Scatter plots of log_2_(fold change) in EWS–FLI binding in A673 ETV6–dTAG cells following 72 h of ETV6 degradation (*y* axis) compared to CRISPR–Cas9-transduced PEDS0009 cells with knockout of *ETV6* (*x* axis). Line indicates Pearson correlation; Pearson correlation value (*R*) is shown. **f**, Heatmaps of FLI1 CUT&RUN performed in control or *ETV6* knockout PEDS0009 cells. Loci shown are defined in Fig. 2h as regions that exhibited increased EWS–FLI binding following ETV6 loss in A673 ETV6–dTAG cells. **g**, Metaplots of FLI1 binding in control or *ETV6* knockout PEDS0009 cells at genomic regions shown in **f** (top). Metaplots of FLI1 binding at loci defined in Fig. 2g as regions that exhibited increased EWS–FLI binding 72 h following ETV6 degradation in EW8 ETV6–dTAG cells. Source data

**Extended Data Fig. 6:**
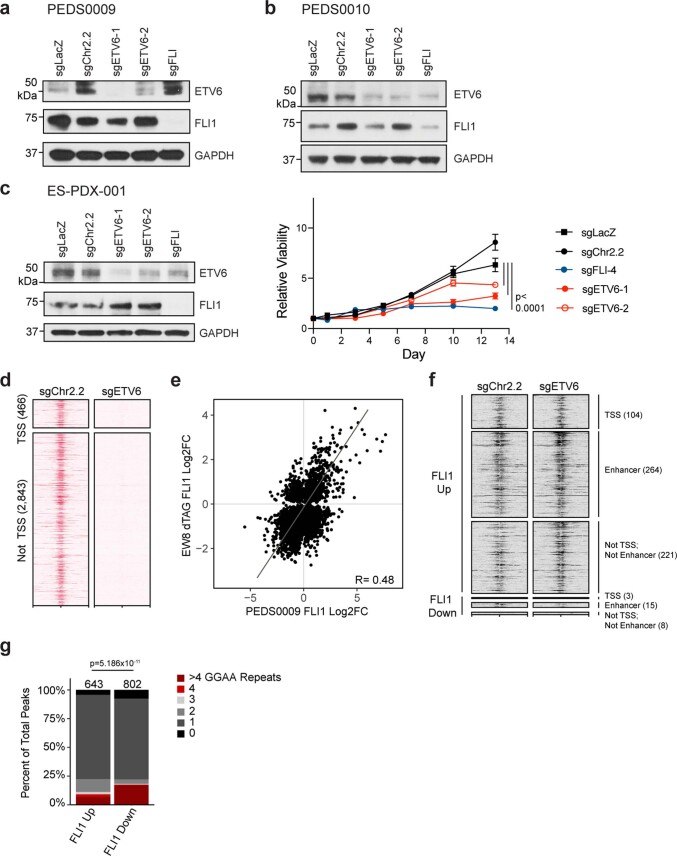
ETV6 competes with EWS/FLI for binding in clinically relevant Ewing sarcoma models. **a and b**. Western blot of cells from the newly derived Ewing sarcoma cell lines, PEDS0009 (**a**) and PEDS0010 (**b**), transduced with CRISPR/Cas9 constructs. Represents one independent experiment. **c**. (Left) Western blot of cells from the Ewing sarcoma patient-derived xenograft, ES-PDX-001, transduced with CRISPR/Cas9 constructs. (Right) Line graphs depicting mean cell viability ±SEM *in vitro* (n = 6 biological replicates). Knockout of *ETV6* and *EWS/FLI* reduced cell growth compared to sgChr2.2 control (2-way ANOVA, Tukey’s multiple comparisons, p-adj = <0.0001). **d**. Heatmaps showing 3-kilobase (kb) windows centered at 3,309 consensus ETV6 binding sites, sub-plotted by overlap within 2.5 kb of transcription start sites (TSS). Shown are ETV6 peaks detected in sgChr2.2 control and *ETV6*-knockout PEDS0009 cells profiled by CUT&RUN. **e**. Scatter plots of log2 fold-change in EWS/FLI binding in EW8 ETV6-dTAG cells following 72 hours of treatment with DMSO or dTAG^V^-1 (y-axis) and in control and *ETV6-*knockout PEDS0009 cells profiled by FLI1 CUT&RUN (x-axis). Pearson correlation value (R) is shown. **f**. Heatmaps of FLI1 CUT&RUN performed in control and *ETV6-*knockout PEDS0009 cells. Loci shown were defined in Fig. 2g as regions that exhibited increased EWS/FLI binding upon ETV6 loss in EW8 ETV6-dTAG cells. **g**. Stacked column plot showing varying lengths of tandem 5′-GGAA-3′ motif repeats occurring at genomic regions exhibiting significantly (CSAW; p < 0.05) increased (FLI1 Up) or decreased (FLI1 Down) EWS/FLI occupancy in PEDS0009 cells upon ETV6 knockout. FLI1 Up regions were more likely than FLI1 Down regions to contain GGAA repeats of 2, 3, or 4 (Fisher Exact test, p = 5.186e-11). Source data

Well-established cancer cell lines may use distinct biological mechanisms to that of primary tumour cells. We therefore tested the relevance of our findings from cell lines in two newly derived Ewing sarcoma cell lines: CCLF_PEDS_0009_T (PEDS0009) and CCLF_PEDS_0010_T (PEDS0010)^64^. *ETV6* knockout impaired cell growth in vitro and colony formation in methylcellulose (Fig. 5a,b and Extended Data Fig. 6a,b). Additionally, we tested cells from a minimally passaged Ewing sarcoma patient-derived xenograft (PDX): ES-PDX-001 (refs. 65, 66). Again, knockout of *ETV6* impaired cell growth in vitro (Extended Data Fig. 6c). In PEDS0009 cells, we observed ETV6 and EWS–FLI binding at previously defined EWS–FLI consensus binding sites (Fig. 5c). Concordant with our cell line data, ETV6 bound to GGAA microsatellites (Fig. 5d), and ETV6 loss resulted in increased EWS–FLI binding at the same loci that exhibited increased EWS–FLI occupancy in cell lines (Fig. 5e–g and Extended Data Fig. 6d,e). Genomic regions that gained EWS–FLI binding were more likely to contain shorter GGAA repeats of 2, 3 or 4 compared with regions that lost EWS–FLI (Extended Data Fig. 6f) (*P* = 5.186 × 10^−11^). These observations in minimally passaged cells were concordant with the data from well-established cell lines.

### ETV6 and EWS–FLI antagonism at *SOX11* is functional

**Extended Data Fig. 7:**
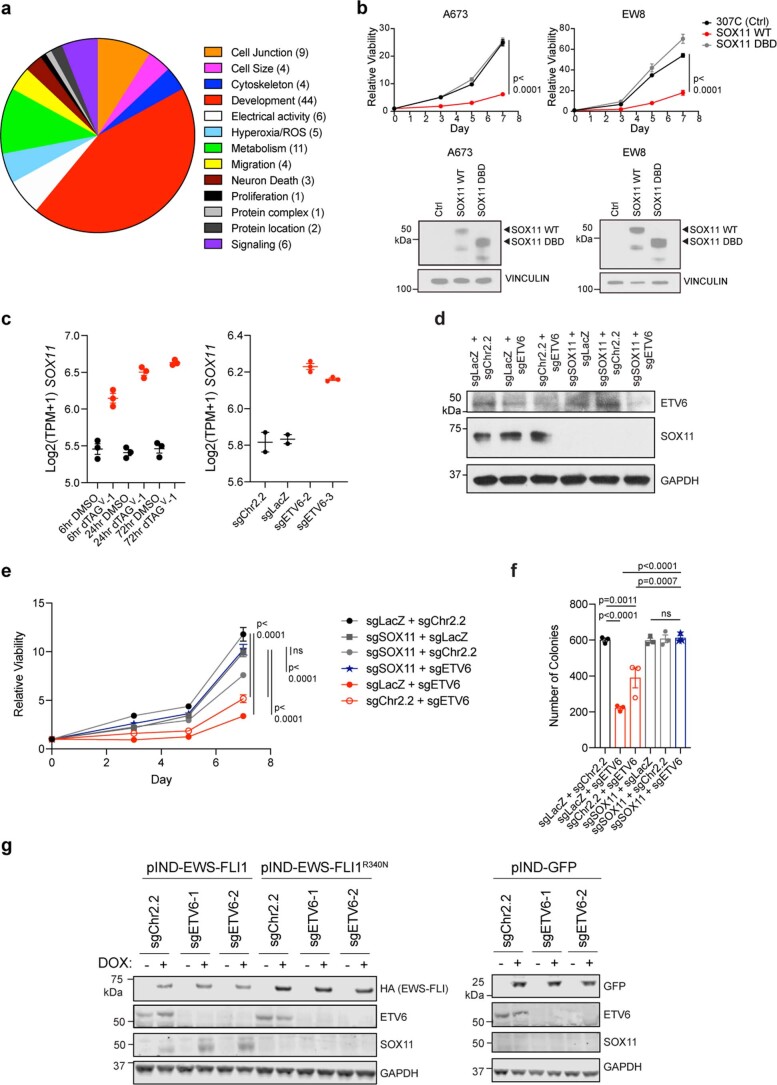
Knock-out of the ETV6-repressed gene, *SOX11*, rescues the phenotype of ETV6 loss. **a**. Top 100 significantly enriched MSigDB c5 Gene Ontology gene set categories in ETV6-repressed genes (24-hour RNA-seq, A673 ETV6-dTAG, parentheses show gene set number; full list in Supplementary Table 19). **b**. Line graphs depicting mean viability ±SEM (n = 8 biological replicates) in A673 (left) and EW8 (right) cells exogenously expressing wild-type *SOX11* (SOX11 WT, red), DBD-deleted mutant *SOX11* (SOX11 DBD, gray), or empty pLX_TRC307 vector control (307 C, black). Wild-type *SOX11* expression reduced viability compared to control (2-way ANOVA, Dunnett’s multiple comparisons, p-adj<0.0001). Western blots show SOX11 and Vinculin loading control. **c**. RNA-seq log2(TPM + 1) ±SEM of *SOX11* expression in A673 ETV6-dTAG cells (left) (n = 3 biological replicates) and in CRISPR/Cas9-perturbed parental A673 cells (right) (controls n = 2 biological replicates; *ETV6-*knockout n = 3 biological replicates). **d**. Western blot of CRISPR/Cas9-perturbed A673 cells. **e**. Mean viability ±SEM (n = 6 biological replicates) of A673 cells shown in **d** *in vitro*. *ETV6* knockout (red open and closed circles) reduced viability compared to control (black circles) (2-way ANOVA, Tukey’s multiple comparisons, p-adj<0.0001). Simultaneous *ETV6* and *SOX11* knockout (blue star) did not reduce viability compared to *SOX11* knockout alone (gray square and circle) (sgSOX11 + sgETV6 vs. sgSOX11+sgLacZ, not significant ‘ns’ p-adj=0.7343) and exhibited greater viability than *ETV6* knockout alone (p-adj<0.0001). **f**. Mean methylcellulose colony number ±SEM (n = 3 biological replicates) formed by A673 cells shown in **d** and **e**. *ETV6* knockout reduced colony number compared to control (2-way ANOVA, Tukey’s multiple comparisons, sgLacZ+sgETV6 vs. sgLacZ+sgChr2.2 p-adj<0.0001, sgChr2.2 + sgETV6 vs. sgLacZ+sgChr2.2 p-adj=0.0011). Simultaneous *ETV6* and *SOX11* knockout did not reduce colony number compared to *SOX11* knockout alone (sgSOX11 + sgLacZ vs. sgSOX11 + sgETV6, ns, p-adj=0.9984) and increased colonies compared to *ETV6* knock-out alone (sgSOX11 + sgETV6 vs. sgLacZ+sgETV6 p-adj<0.0001; vs. sgChr2.2 + sgETV6 p-adj=0.0007). **g**. Western blot of rhabdomyosarcoma RD cells expressing doxycycline-inducible HA-tagged GFP, wild-type EWS/FLI, or DNA binding-incompetent R340N mutant EWS/FLI, in combination with CRISPR/Cas9 perturbation. One independent experiment. Source data

We next asked whether the antagonistic relationship between EWS–FLI and ETV6 is responsible for the dependency of Ewing sarcoma cells on ETV6. Almost half of the gene sets enriched in ETV6-repressed genes were related to developmental pathways (Extended Data Fig. 7a and Supplementary Table 18), and 46 of these included *SOX11* (Supplementary Table 19). *SOX11* expression exerts context-dependent effects on cancer cell survival, growth and metastasis^67,68^. *SOX11* acts as an oncogene in mantle cell lymphoma^69^ and promotes metastasis in breast cancer^70,71^. Conversely, it also reduces proliferation and metastasis in prostate cancer^72^ and induces differentiation of glioma cells^73^. In Ewing sarcoma cells, the exogenous expression of *SOX11* impaired cell growth, whereas the expression of a DBD-deleted mutant did not (Extended Data Fig. 7b). These results provide support for a tumour-suppressive role for SOX11 activity.

**Fig. 6:**
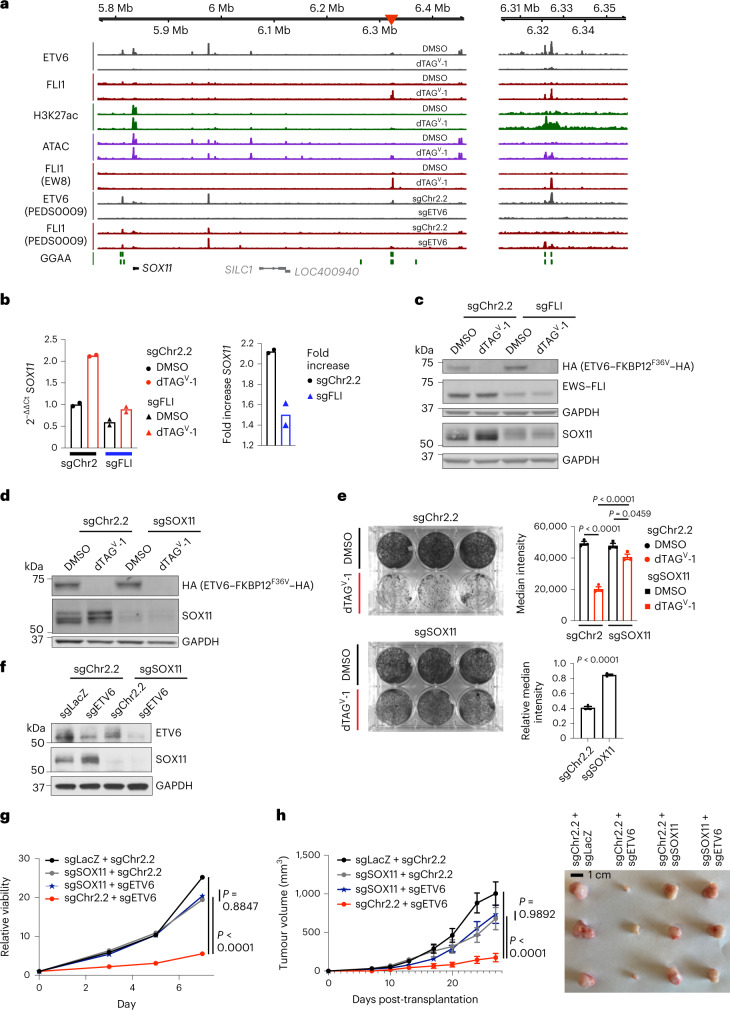
Knockout of the ETV6-repressed gene *SOX11* rescues the phenotype of ETV6 loss. **a**, Left: Gviz-generated view of the *SOX11* locus. Top four tracks show data generated in A673 ETV6–dTAG cells: ETV6, 24 h ETV6-FKBP12^F36V^–HA CUT&Tag; FLI1, 6 h EWS–FLI ChIP-seq; H3K27ac, 6 h H3K27ac ChIP-seq; ATAC, 72 h ATAC-seq. FLI1 (EW8) shows 6 h EWS–FLI ChIP-seq in EW8 ETV6–dTAG cells. ETV6 (PEDS0009) and FLI1 (PEDS0009) show CUT&Tag for ETV6 and EWS–FLI, respectively, in PEDS0009 cells. GGAA shows tandem GGAA motif repeats. The red arrowhead indicates an enhancer region assigned to *SOX11*, the nearest expressed gene; *SILC1* and *LOC400940* are not expressed and labeled in grey^74^. Right: magnified view of the enhancer. **b**, *SOX11* expression by qPCR, as described in Fig. 4g. **c**, Western blot of cells shown in **b** at 96 h. HA, EWS–FLI, GAPDH bands are also shown in Fig. 4h. **d**, Western blot of sgChr2.2-transduced or sgSOX11-transduced A673 ETV6–dTAG cells cultured in DMSO or dTAG^V^-1. Represents two independent experiments. **e**, Left: cells in **d** stained with crystal violet. Right, top: bar plots showing mean ± s.e.m. of median stain intensity per well (one-way ANOVA, *n* = 3 biological replicates, Sidak’s multiple comparisons; DMSO versus dTAG^V^-1 sgChr2.2, *P* adjusted < 0.0001, sgSOX11, *P* adjusted = 0.0459; sgChr2.2 versus sgSOX11 dTAG^V^-1, *P* adjusted < 0.0001). Right, bottom: relative median intensity comparing dTAG^V^-1-treated with DMSO-treated wells (two-tailed *t*-test, *n* = 3, *P* < 0.0001). Represents two independent experiments. **f**, Western blot of TC32 cells transduced with CRISPR–Cas9 constructs in combination. Represents one experiment. **g**, Line graph depicting mean viability in vitro (*n* = 6 biological replicates, s.e.m. bars too small to depict) of cells in **f**. *ETV6* knockout alone (red) reduced viability compared to control (black) (two-way ANOVA, Tukey’s multiple comparisons, *P* adjusted < 0.0001). Simultaneous *ETV6* and *SOX11* knockout (blue star) did not reduce viability compared to *SOX11* knockout alone (grey) (NS, *P* adjusted = 0.8847) and exhibited greater viability than *ETV6* knockout alone (red) (*P* adjusted < 0.0001). **h**, Left: Line graph depicting mean subcutaneous tumour volume (mm^3^) ± s.e.m. (*n* = 6 tumours, biological replicates) formed by cells shown in **f**. *ETV6* knockout alone reduced tumour volume (two-way ANOVA, Tukey’s multiple comparisons, *P* adjusted < 0.0001). Simultaneous *ETV6* and *SOX11* knockout did not reduce tumour growth (NS, *P* adjusted = 0.9892) and exhibited greater growth than *ETV6* knockout alone (*P* adjusted < 0.0001). Right: representative tumours from each condition. Source data

We observed differential EWS–FLI binding at a distal enhancer that mapped to *SOX11* as the nearest expressed gene (Fig. 6a, left). RNA-seq data from the Cancer Cell Line Encyclopedia^74^ show that the neighbouring genes, *SILC1* and *LOC400940*, are not expressed in Ewing sarcoma. This enhancer occurred at tandem GGAA repeats and exhibited increased EWS–FLI binding, H3K27ac abundance and chromatin accessibility following ETV6 loss (Fig. 6a, right). RNA-seq confirmed that *SOX11* is repressed by ETV6-FKBP12^F36V^ in dTAG cells and by endogenous ETV6 in parental A673 cells (Extended Data Fig. 7c). EWS–FLI was required for *SOX11* upregulation after ETV6 loss (Fig. 6b,c). Knockout of *SOX11* in A673 ETV6–dTAG cells (Fig. 6d) rescued the effects of ETV6 degradation (Fig. 6e). Additionally, knockout of *SOX11* in A673 and TC32 cells (Fig. 6f and Extended Data Fig. 7d) rescued *ETV6* knockout (Fig. 6g and Extended Data Fig. 7e,f). In vivo, we observed rescue in TC32 cells grown as subcutaneous tumours in mice (Fig. 6h). These findings support the hypothesis that ETV6 dependency is specific to Ewing sarcoma cells because ETV6 constrains EWS–FLI activation of *SOX11* expression.

Finally, we asked whether co-regulation at *SOX11* by ETV6 and EWS–FLI could be recapitulated with ectopic expression of EWS–FLI. In rhabdomyosarcoma RD cells, we exogenously expressed wild-type EWS–FLI or the R340N DNA-binding mutant of EWS–FLI, which cannot bind to DNA^75^. SOX11 protein expression was induced by wild-type EWS–FLI but not the mutant (Extended Data Fig. 7g). Knockout of *ETV6* further upregulated SOX11 abundance in the setting of wild-type EWS–FLI but not in the context of mutant EWS–FLI expression (Extended Data Fig. 7g). These findings demonstrate that the DBD of EWS–FLI is required for its activation of *SOX11* expression, an activity that is repressed by ETV6.

## Discussion

In this study, we discovered an oncogenic mechanism underlying the paediatric cancer Ewing sarcoma. We demonstrated that the ETS TF ETV6 is a selective dependency in Ewing sarcoma because it antagonizes the transcriptional activity of EWS–FLI at ETS motifs. To our knowledge, this report constitutes the first description of transcriptional constraint of a fusion TF on chromatin as a crucial driver of tumour growth.

Although previous studies have described specific TFs as dependencies that reinforce the EWS–FLI transcriptional programme in Ewing sarcoma^32–35,37^, including in CRCs^40^, these targets were not identified in DepMap screening as selective gene dependencies. Instead, our discovery that ETV6 constrains EWS–FLI activity highlights a distinct, but equally central, epigenetic mechanism that drives tumour growth and reveals an unexpected contrast between Ewing sarcoma and other paediatric tumours in which CRCs are functionally dominant.

Cancer cells frequently co-opt mechanisms that underlie normal development^76^. The competition between EWS–FLI and ETV6 in Ewing sarcoma bears resemblance to a mechanism of ETS TF competition governing cell-fate decisions in developing *Drosophila*. Pointed, the activating orthologue of human Ets-1, competes for binding at ETS motifs within specific enhancers with Yan, the repressive orthologue of ETV6, to regulate the expression of key differentiation genes in distinct tissues^77–81^. Here we described a similar mechanism that has been co-opted in cancer to regulate the transcriptional output of a fusion TF.

The epigenetic activity of ETS TFs other than EWS–FLI may contribute to the phenotype of ETV6 loss. Notably, *ETV7*, the homologue of *ETV6*, is not expressed in Ewing sarcoma cells (Supplementary Table 20), and we did not observe strong changes in the expression of other ETS TFs with ETV6 loss. The maximum change exhibited by one gene was roughly threefold, and only five genes displayed a significant alteration in expression across the models evaluated. Moreover, none of the genes that displayed a change in expression were scored as dependencies or tumour suppressors in DepMap in Ewing sarcoma.

Although most human TF families contain paralogues that are co-expressed within distinct cell types^82–84^, an understanding of their interactions at shared motifs is lacking. We began to unravel key *cis* regulatory principles that distinguish the specific functions of ETV6 and EWS–FLI, the antagonism of which on chromatin frequently occurred at shorter 5′-GGAA-3′ repeats. As the pathogenesis of EWS–FLI is typically associated with its activity at longer repeats or true microsatellites, we highlight a previously undescribed *cis* regulatory role for shorter GGAA repeats in this disease, which facilitates ETV6 fine-tuning of EWS–FLI. Even though the reconstitution of EWS–FLI for biochemical assays has been a challenge for the field, future work is needed to delineate the precise GGAA repeat code that determines the activities of each TF. Similarly, ETV6 and wild-type FLI1 proteins can engage in an inhibitory heterodimer^85^, an interaction mediated by the amino-terminal Pointed (PNT) domain of ETV6, and further studies are needed to determine whether ETV6 and EWS–FLI engage in a protein–protein interaction. Notably, however, our experiments using an ETS DBD-deleted mutant of ETV6, with an intact PNT domain, demonstrated that the DNA-binding activity of ETV6 is crucial to its function in Ewing sarcoma.

ETV6 is a master TF in normal development and is recurrently mutated in cancer. *ETV6* mutations include deletions and chromosomal translocations involving 30 distinct gene partners^53^. Germline and somatic loss-of-function mutations frequently occur in pre-malignant disorders and leukaemias. For example, in B cell acute lymphoblastic leukaemia, *ETV6* deletions frequently co-occur with *ETV6*–*RUNX1* rearrangements, which result in biallelic loss of the ETV6 protein^86,87^. Chromosomal translocations also fuse the N terminus of ETV6 with the tyrosine kinase domain from a number of receptor tyrosine kinases, which facilitate constitutive autophosphorylation and growth signalling^53^. *ETV6*, however, has not been reported as recurrently mutated in Ewing sarcoma^42–44^. Furthermore, *ETV6* is not regulated by EWS–FLI (Supplementary Table 4) and does not exhibit a marked pattern of expression specific to this cancer type (Extended Data Fig. 1d). Nonetheless, we discovered its role as a crucial tumour-type-selective dependency in regulating EWS–FLI activity. As such, this report reaffirms the importance of performing unbiased functional screens at scale to reveal oncogenic mechanisms sustained by wild-type proteins.

Our findings suggest that a hallmark of Ewing sarcoma biology may involve the reliance on mechanisms constraining EWS–FLI activity to promote tumour growth. Indeed, we previously described mechanisms mediated by an E3 ligase (TRIM8) and cohesin that restrain EWS–FLI activity^48,66,88^. Here we discovered a distinct mechanism in support of an EWS–FLI Goldilocks phenomenon^66^ that is operative on chromatin. Future translational efforts could ultimately seek to modulate the activity of this pharmacologically challenging protein, either by decreasing or paradoxically increasing its activity.

In conclusion, we discovered the oncogenic role of TF competition on chromatin between a mutant TF and a wild-type paralogue. Our work contributes to an understanding of the dysregulated epigenetic mechanisms that can promote cancer, raising the possibility that similar mechanisms are relevant in other disease contexts.

## Methods

Our research complied with all ethical guidelines determined by the Dana-Farber Cancer Institute Institutional Animal Care and Use Committee under Animal Welfare Assurance number D16-00010 (A3023-01). No human studies were performed. In Extended Data Fig. 6c, a minimally passaged cell line previously derived in our laboratory^66^ from a previously characterized Ewing sarcoma PDX^65^ (HSJD-ES-PDX-001) was studied. As such, this experiment was performed in vitro and did not involve the use of animals. As previously described^65^, this PDX originated from a biopsy in a 21.7-year-old patient whose sex was not reported^65^. It was collected with informed consent without compensation under an Institutional Review Board-approved protocol at Sant Joan de Déu Hospital (HSJD, Barcelona, Spain), protocol number HSJD 135/11 (ref. 65).

### CRISPR–Cas9 screen dependency analysis

All genome-scale dependency data are available at the DepMap portal website: https://depmap.org. DepMap AVANA 21Q1 dependency data were used (18,333 genes in 808 cell lines, https://figshare.com/articles/dataset/public_21q1/13681534). Twelve cell lines were not included in the analyses: four cell lines are classified as engineered lines; the origin of one cell line, CHLA57, is unknown as it is incorrectly identified as Ewing sarcoma; seven cell lines are listed as commonly misidentified cell lines in the ICLAC Register of Misidentified Cell Lines (https://iclac.org/databases/cross-contaminations/). Therefore, dependency data for 796 cell lines were examined. CERES gene effect scores were calculated as previously described^41,90^. A lower CERES gene effect score indicates an increased likelihood that a specific gene is required for viability in that cell line. A CERES score of 0 indicates that gene deletion exhibited no effect on growth, whereas a score of −1 is comparable with the median of all commonly essential genes, that is, genes that were essential for growth in nearly every cell line across the entire screen. Tumour-type-enriched ‘selective’ dependencies were determined by performing a two-class comparison between the gene effect scores for cell lines of each tumour type (in-group) and the remainder of all other cell lines in the screen (out-group) for a specific gene as previously described^41^. In brief, effect size was calculated as the difference in the mean gene effect dependency score in the in-group compared with that in the out-group. In addition to two-sided *P* values, one-sided *P* values were generated to test whether the in-group exhibited, on average, greater or lesser dependency on a specific gene than the out-group. All *P* values were corrected for multiple hypothesis testing using the Benjamini–Hochberg correction and reported as *q* values. Tumour-type-enriched dependencies were identified in each tumour type as those with a *q* value of <0.05 and with a negative effect size (the mean of dependency gene effect score was more negative in the in-group than in the out-group). The same analyses were performed on the genome-scale CRISPR–Cas9 screens using the Broad Institute GeCKO library (18,478 genes in 43 cell lines, https://figshare.com/articles/dataset/DepMap_GeCKO_19Q1/7668407) as well as the Sanger library (17,799 genes in 318 cell lines, https://figshare.com/articles/dataset/Project_SCORE_processed_with_CERES/9116732/1).

### Cancer cell line and primary tumour gene expression

RNA-seq gene expression data from the Cancer Cell Line Encyclopedia^74^ were downloaded (19,177 genes in 1,376 cell lines) from the 21Q1 DepMap portal website (https://depmap.org). Tumour-type-enriched expression for each gene was calculated by performing a two-class comparison between the log_2_(transcripts per million (TPM) + 1) gene expression for cell lines of each tumour type (in-group) and the remainder of all other cell lines profiled (out-group). All *P* values were corrected for multiple hypothesis testing using the Benjamini–Hochberg correction and reported as *q* values. RNA-seq gene expression data for primary tumours were downloaded from the Treehouse Childhood Cancer Initiative^45^ (UCSC Genomics Institute, https://treehousegenomics.soe.ucsc.edu/public-data).

### Cell samples and culture

All cell lines were genotyped by short tandem repeat analysis and tested for *Mycoplasma*. Whole-exome sequencing and RNA-seq were performed to validate cell line identity^43^. The A673 cell line was purchased from the American Type Culture Collection (ATCC, CRL-1598). EW8 (originally derived by P. Houghton^91^) and TC32 (originally derived by T. Triche^92^) cell lines were obtained from the Golub Lab. A673 and EW8 cells were grown in Dulbecco’s modified Eagle’s medium (DMEM) (Thermo Fisher Scientific, MT10013CM), supplemented with 10% FBS (Sigma-Aldrich, F2442) and 1% penicillin–streptomycin (Life Technologies, 15140163). TC32 cells were grown in Roswell Park Memorial Institute (RPMI)-1640 medium (Thermo Fisher Scientific, MT10040CM), supplemented with 10% FBS and 1% penicillin–streptomycin. The PEDS0009 and PEDS0010 cell lines were obtained from the Cancer Cell Line Factory (Broad Institute) and were derived as previously described^64^. They were cultured in RPMI-1640 supplemented with 10% FBS and 1% penicillin–streptomycin. The Ewing sarcoma PDX (HSJD-ES-PDX-001) was provided by J. Mora (HSJD)^65^. To generate the minimally passaged cell line (ES-PDX-001), PDX tumours were processed as previously described^66^. The RD cell line (ATCC, CRL-7731) was a gift from the DepMap group at the Broad Institute. RD cells were cultured in RPMI-1640 supplemented with 10% FBS.

### CRISPR–Cas9 genome editing of Ewing sarcoma cell samples

The lentiCRISPRv2 plasmid backbone^93^ (Addgene, 52961) encoding the Cas9 nuclease was digested with the restriction endonuclease BsmbI (Esp3I) (Thermo Fisher Scientific, FERFD0454) and gel extracted (Qiagen, 28704). Synthetic oligonucleotides containing gene-targeting single guide RNA (sgRNA) sequences and adapter sequences (provided below) were ligated into the restriction digest site. Oligonucleotides were purchased from Integrated DNA Technologies (IDT), annealed and end-phosphorylated using T4 polynucleotide kinase (New England Biolabs, M0201S). Ligated vectors were transformed into One Shot Stbl3 *Escherichia* *coli* (Life Technologies, C737303), shaken at 37 °C for 1 h and grown overnight on 100 µg ml^–1^ ampicillin Luria broth plates (Teknova, L1004). Picked colonies were grown for 8–16 h in 100 µg ml^–1^ carbenicillin Luria broth starter cultures. Plasmids were DNA-extracted (Qiagen, 27104) and submitted for Sanger sequencing validation by Genewiz. Validated clones were cultured overnight, and plasmids were extracted (Qiagen, 12963).

CRISPR–Cas9 constructs were packaged into lentiviral particles. HEK293T cells were seeded at 70–80% confluence in 10 cm plates and co-transfected with 9 µg of lentiCRISPRv2-sgRNA construct plasmid, 0.9 µg pVSVg plasmid (Addgene 8454) and 9 µg pPAX2 plasmid (Addgene 19319) using Lipofectamine 3000 (Life Technologies, L3000015). Sixteen hours after transfection, medium was supplemented with 30% FBS. The following day, virus-containing medium was collected, 0.45 µm sterile-filtered and stored at −80 °C.

One million Ewing sarcoma cells were seeded per well of a 6-well plate and spin-infected using 2 ml of virus and 8 µg ml^–1^ polybrene (Santa Cruz Biotechnology, SC-134220) at 37 °C at 1,190 r.c.f. for 30 min. The following day, fresh medium containing 1 µg ml^–1^ puromycin (InvivoGen, ant-pr-1) was added. Cells were selected for at least 48 h. In experiments requiring knockout of two genes, the cells were co-transduced with constructs encoding two distinct sgRNAs, each conferring resistance to either puromycin or blasticidin. Cells were selected with 1 µg ml^–1^ puromycin and 5 µg ml^–1^ blasticidin (Life Technologies, A1113903) for at least 5 days. Separate samples of non-infected cells treated with drug were used to confirm cell death.

All sgRNA sequences used in the Broad Institute AVANA CRISPR–Cas9 screen are available for download at the DepMap Portal (https://depmap.org). The following sequences were used: sgETV6-1: 5′-GCAGCCAATTTACTGGAGCA-3′, sgETV6-2: 5′-GCAGGGATGACGTAGCCCAG-3′, sgETV6-3: 5′-GTGTGTGTATAGAGTTTCCA-3′, sgETV6-4: 5′-GTTATGGTGCACATTATCCA-3′, sgSOX11: 5′-CACCGGAAGATCCCGTTCATCCGGG-3′, sgFLI1: 5′-CACCGTGTCGGAGAGCAGCTCCAGG-3′. As previously described^94,95^, sgChr2.2 served as a cutting control and targets a gene desert on chromosome 2, 5′-GGTGTGCGTATGAAGCAGTG-3′; sgLacZ served as a non-targeting control and targets a non-human gene, 5′-AACGGCGGATTGACCGTAAT-3′. For ligation into the lentiCRISPRv2 plasmid, the additional bases 5′-CACCG-3′ were added to the 5′ end of the forward sequence. 5′-AAAC-3′ and 5′-C-3′ were added at the 5′ and 3′ ends of the reverse sequence, respectively.

### Generation, culture and application of polyclonal dTAG cell lines

Four sgRNAs targeting exon–intron junctions of the endogenous *ETV6* locus were cloned, as described above, into the lentiCRISPRv2 plasmid backbone conferring blasticidin resistance (Addgene, 83480). The following sgRNA sequences were used: (1) sgETV6-endo-1, 5′-TCCTGCTCAGTGTAGCATTA-3′, (2) sgETV6-endo-2, 5′-GAACACTCACGCAGGTGCGC-3′, (3) sgETV6-endo-3, 5′-TCCAGACTCTCACCTGAATG-3′, and (4) sgETV6-endo-4, 5′-AGTTCATAGAGCACATCACC-3′. A codon-optimized gBlock encoding coding sequences of *ETV6* was cloned into the pLEX_305 vector backbone (Addgene, 91798) to C-terminally tag the ETV6 protein with the FKBP12^F36V^ protein domain and a HA epitope tag. A673 and EW8 parental Ewing sarcoma cells were spin-infected and selected with puromycin and blasticidin as described above. Cells were cultured in DMEM supplemented with 15% FBS, 1% penicillin–streptomycin and 0.5 µg ml^–1^ puromycin and 2.5 µg ml^–1^ blasticidin to maintain selection. Cells were split at a ratio of 1:5 every other day.

The dTAG^V^-1 molecule was provided by the Gray Laboratory (Dana-Farber Cancer Institute, Boston, MA) and used at a stock concentration of 10 mM suspended in DMSO. For 6, 24 and 72 h RNA-seq time points, A673 ETV6–dTAG cells were seeded at 1 million cells per 6 cm dish, 0.5 million cells per 6 cm dish and 0.5 million cells per 10 cm dish, respectively. EW8 ETV6–dTAG cells were seeded at 0.75 million cells per 6 cm dish, 0.5 million cells per 10 cm dish and 0.2 million cells per 10 cm dish, respectively. For each time point, three separate dishes were seeded and treated per DMSO or dTAG^V^-1 condition. Cells were collected for total RNA extraction and western blot validation (described below). For 6 and 72 h ChIP-seq time points, A673 ETV6–dTAG cells were seeded at 5 million cells per 15 cm dish and 1 million cells per 15 cm dish, respectively. EW8 ETV6–dTAG cells were seeded at 7.7 million cells per 15 cm dish and 1 million cells per 15 cm dish, respectively. Twenty-four hours after seeding, existing medium was exchanged for DMSO or dTAG^V^-1-containing medium. For all experiments, dTAG^V^-1 was used at a final concentration of 1 µM. Equivalent volumes of DMSO were used as control.

A673 ETV6–dTAG cells were further perturbed to knockout *EWS–FLI* or *SOX11*. sgRNAs targeting *FLI1* (5′-TGTCGGAGAGCAGCTCCAGG-3′) or *SOX11* (5′-GAAGATCCCGTTCATCCGGG-3′) from the Broad AVANA screen were cloned into lentiCRISPRv2 as described above. In total, 250,000 cells per well of a 6-well plate were spin-infected with 2 ml virus. Knockout was validated by western blotting.

### Relative viability studies

Cells transduced with lentivirally packaged CRISPR–Cas9 constructs were seeded in 384-well plates at densities of 3,500 (TC32), 2,000 (A673), 250 (EW8) and 1,000 (PEDS0009, PEDS0010 and ES-PDX-001) cells per well suspended in 40–50 µl of medium per well containing 0.5–1 µg ml^–1^ puromycin. Cells from each condition were grown separately in 6–8 wells per plate across 4 plates, which corresponded to day 0, 3, 5 and 7 time points. Wells at plate edges were filled with 50 µl of PBS to maintain humidity. To measure cell viability, 10 µl of CellTiter-Glo reagent (Promega, G7573) was added to each well, luminescing at an intensity proportional to ATP abundance, and plates were shaken at room temperature for 15 min. Luminescence was measured using a FLUOstar Omega microplate reader (BMG LabTech). Relative viability was calculated by dividing the luminescence measurement of each well on day 7 by luminescence at day 0 using Microsoft Excel 16.50. In parallel, whole cell lysate was collected on day 7 for western blotting to confirm *ETV6* knockout. Statistics shown compare mean relative viability between conditions at day 7, analysed using GraphPad Prism 9.0.0.

### Anchorage-independent growth

A 16-gauge blunt-end needle was used to transfer 12 ml of semi-solid methylcellulose-based medium (Stemcell Technologies, 03814) to a 50 ml conical tube and 3 ml of cell suspension containing 15,000 (A673 and /TC32), 5,000 (EW8) or 20,000 (PEDS0009, PEDS0010 and ES-PDX-001) cells. The mixture was vortexed and left at room temperature for 10–15 min until bubbles dissolved. A blunt-end needle was used to transfer 3 ml of the mixture to separate 6 cm dishes, which were placed inside a 15 cm plate containing a PBS-filled 6 cm dish used to maintain humidity. Colonies were stained 7 days later by adding 1 ml of a 1:1 mixture of PBS and MTT dye (Roche Diagnostics, 11465007001) per dish and incubating for 30–45 min at 37 °C. Colonies in each dish were imaged using an ImageQuant LAS 4000 imager (GE Healthcare) and quantified using ImageQuant TL 8.2 software (Cytiva). In parallel, whole cell lysate was collected from cultured cells for western blotting.

### Flow cytometry and cell cycle analysis

Cell cycle analysis was performed using Click-iT Plus EdU Alexa Fluor 647 Flow Cytometry Assay kits (Life Technologies, C10424) per kit instructions with minor modifications. Cells were seeded and cultured separately before being pulsed with 10 µM of the modified nucleotide analogue 5-ethynyl-2′-deoxyuridine (EdU) for 90 min at 37 °C. Around 1–2 million cells per sample were trypsinized, washed, fixed, permeabilized and then treated with a reaction cocktail containing Alexa Fluor-647-conjugated picolyl azide to label incorporated EdU. Cells were stained with a RNAse-containing propidium iodide solution (Cell Signaling, 4087S) for 45 min at 37 °C. Cells were analysed by flow cytometry at 5,000–10,000 cells per sample on a BD FacsCelesta instrument. Live cells were gated using FSC-A and SSC-A. The data were analysed using FlowJo v.10.6.1 software. Cells were collected from each sample for western blotting.

### Mouse studies

All mouse studies were approved by the Dana-Farber Cancer Institute Institutional Animal Care and Use Committee (Animal Welfare Assurance number: D16-00010 (A3023-01)) and were performed in accordance with NIH guidelines for the humane care and use of animals. The intramuscular mouse xenograft experiment (Fig. 1h,i) studied immunodeficient NOD.Cg-Prkdcscid Il2rgtm1Wjl/SzJ (NSG) mice ordered from Jackson Laboratory in a semi-orthotopic manner as previously described^48^. A673 cells were lentivirally transduced to express luciferase and CRISPR–Cas9 constructs targeting *ETV6*. These cells were intramuscularly implanted in the hindlimbs of 7-week-old female mice. On the day of implantation, cells were suspended in a 1:1 mixture of PBS and Matrigel (Thermo Fisher Scientific, CB40230C) and injected directly into the hindlimb cranial thigh muscle, away from the sciatic nerve, at a concentration of 50,000 cells per mouse in 50 µl. Five mice per condition (sgChr2.2, sgLacZ, sgETV6-1 and sgETV6-2) were implanted. Disease progression was monitored by serial bioluminescence imaging of the whole body. Bioluminescence was measured 10 min following subcutaneous injection of luciferin using a PerkinElmer IVIS Spectrum (exposure time, 0.5–180 s; binning, 2–16; luminescent, 25,000) to determine the maximum bioluminescence exhibited by each mouse. Mice in each condition were imaged at the same time. Mice were euthanized at the end point. Lung and liver tissue samples were collected following euthanasia and placed in a 6-well dish for bioluminescence imaging. Subcutaneous mouse xenograft experiments were conducted in Jackson NSG mice (Extended Data Fig. 2d) and CrTac:NCR-Foxn1<nu>(nude) mice from Taconic Biosciences (Fig. 6h). In the former study, 12-week-old males were used. In the latter study, 6–8-week-old females were used. Cells were suspended in a 7:3 mixture of culture medium and Matrigel and injected bilaterally subcutaneously into sublethally irradiated mice at 3 million cells in 100 μl. Three to four mice per condition received transplants. Tumours were measured with calipers serially twice weekly.

Animals were euthanized when tumours reached maximal 2 cm in at least one dimension or a humane end point such as ulceration or reduced mobility, in adherence to the NIH/NCI guidelines on limits of tumour size (equal to or less than 2.0 cm per tumour in any one dimension). This limit was not exceeded. Randomization was not appropriate in any study as drug treatments were not used. Mice from the same conditions were kept in different cages to minimize confounding environmental factors. Mice were housed with strictly controlled temperature and humidity and kept on 12-h light and dark cycles. No statistical methods were used to predetermine sample sizes, but sample sizes were similar to those reported in previous publications in which statistical significance was achieved^48,66^. Data distribution was assumed to be normal, but this was not formally tested, with the exception of data shown in Fig. 1i, for which the data were not normal (Shapiro–Wilk *P* < 0.05) and thus log-transformed. Data collection and analysis were not performed blind to the conditions of the experiments. No animals or data points were excluded from the analyses.

### Crystal violet staining and quantification

Cell samples were cultured separately and re-seeded at normalized cell densities (50,000 cells per well in 6-well plate) every 5 days with refreshed DMSO or 1 μM dTAG^V^-1. On day 20, each well was incubated with 1 ml of crystal violet stain, composed of 20% methanol and 1% w/v crystal violet powder (Sigma Aldrich, C6158) at room temperature for 20 min. Wells were washed with 3 ml of deionized H_2_O five times and dried at room temperature. Plates were imaged using an ImageQuant LAS 4000 imager (GE Healthcare). The median intensity of stain in each well was quantified using ImageQuant TL 8.2 image analysis software (Cytiva).

### Western blotting

Cells were lysed using cell lysis buffer (Cell Signaling Technology, 9803S), supplemented with protease inhibitor (Sigma Aldrich, 11836170001) and phosphatase inhibitor (Sigma Aldrich, 04906837001). Protein quantification of whole cell lysate was measured using a Bradford-based colorimetric assay (Bio-Rad, 5000006). Around 50–60 µg of whole cell lysate was mixed with loading buffer (Life Technologies, NP0007), reducing buffer (Life Technologies, NP0009) and water and heated to 75 °C for 10 min. Samples were loaded onto 4–12% bis-tris 10-well gels (Life Technologies, NP0335BOX) and run at 100 V for 30 min followed by 150 V for 90 min using MOPS buffer (Life Technologies, NP0001). Gels were transferred to polyvinylidene difluoride membranes (Thermo Fisher Scientific, IPVH00010) at 100 V for 90 min using transfer buffer (Boston BioProducts, BP-190-1L) at 4 °C. Membranes were blocked in milk (Cell Signaling Technology, 9999S) for 60 min at room temperature. Membranes were rocked overnight at 4 °C in a solution of Tris-buffered saline and Tween-20 (TBST; Cell Signaling Technology, 9997S) containing 5% w/v BSA (Research Products International, A30075-1000.0), 0.02% sodium azide (Santa Cruz Biotechnology, SC-208393) and primary antibody. The following day, membranes were washed in TBST five times, 5 min per wash. For a subset of western blots, membranes were rocked for 1 h at room temperature in milk containing 1:5,000 dilution of HRP-conjugated secondary antibody against mouse (Cell Signaling Technology, 7076S) or rabbit (Cell Signaling Technology, 7074S). Membranes were then washed in TBST three times and immersed in a solution containing chemiluminescent substrate (Life Technologies, 34076), allowed to develop for 1 min, then imaged using film (Thermo Fisher Scientific, PI34091). Other western blots were imaged using a LI-COR system. Membranes were rocked in a TBST solution containing a 1:10,000 dilution of secondary antibody against mouse (LI-COR Biosciences, 926-32210) and rabbit (LI-COR Biosciences, 926-68071) and 1:10,000 dilution of 10% SDS solution (Life Technologies, 15553027) for 1 h at room temperature. Membranes were washed in TBST three times and then briefly rinsed in PBS and imaged on an Odyssey CLx machine at medium resolution (ImageStudioLite 5.2.5).

The following primary antibodies were used at the following dilutions: anti-GAPDH at 1:10,000 (2118S, rabbit, monoclonal, Cell Signaling Technology); anti-ETV6 primary at 1:500 (WH0002120M1-100UG, mouse, monoclonal, Sigma Aldrich or SC-166835, mouse, monoclonal, Santa Cruz Biotechnology); anti-HA at 1:2,000 (3724S, rabbit, monoclonal, Cell Signaling Technology); anti-PARP at 1:1,000 (9542S, rabbit, polyclonal, Cell Signaling Technology); anti-cleaved caspase-3 at 1:1,000 (9664S, rabbit, monoclonal, Cell Signaling Technology); anti-FLI1 primary antibody at 1:1,000 (ab15289, rabbit, polyclonal, Abcam); anti-FAS at 1:500 (SC-8009, mouse, monoclonal, Santa Cruz Biotechnology); anti-SEMA5B at 1:500 (PA5113369, rabbit, polyclonal, Thermo Fisher Scientific); anti-BCL11B at 1:500 (12120S, rabbit, monoclonal, Cell Signaling Technology); and anti-SOX11 at 1:1,000 (58207S, rabbit, monoclonal, Cell Signaling Technology).

### *SOX11* overexpression

Complementary DNAs of wild-type *SOX11* and *SOX11* mutants harbouring a deletion of the DBD (H48-R119) were synthesized as gBlocks fragments (IDT), and then cloned into a pLX_TRC307 lentiviral expression vector co-expressing a puromycin resistance gene (obtained from the Genetic Perturbation Platform at the Broad Institute) using a Gibson Assembly Cloning kit (New England Biolabs E5510S). Constructs were lentivirally delivered to cells as described above.

### Inducible *EWS*–*FLI* overexpression

The previously described vector pINDUCER20-EWS/FLI-HA^66^, which encodes HA epitope-tagged EWS–FLI, was mutated to create the R340N mutation^96^ by site-directed mutagenesis using a NEB Q5 Quick Change Site Directed Mutagenesis kit (E0554) using the following primers: forward 5′-CCGGGCCCTCAATTATTACTATGATAAAAAC-3′; reverse 5′ CTCAGCTTGTCGTAATTC-3′. The correct mutation was confirmed by Sanger sequencing with both a forward and reverse sequencing primer (forward: 5′-TCCCACACCGACCAGTCCTCAC-3′; reverse: 5′-AGACTGCCTTGGGAAAAGCGCC-3′). pINDUCER20-GFP-HA vector was used as a control. RD cells stably expressing these three vectors were generated. For knockout experiments, RD cells stably expressing the inducible vectors were transduced with sgRNAs targeting Chr2.2 (cutting control) or *ETV6*. Expression of EWS–FLI was induced using 1 µg ml^–1^ doxycycline replenished every 48 h.

### Rescue of *ETV6* knockout with wild-type and ETS-deleted *ETV6* overexpression

DNA fragments encoding codon-optimized *ETV6* wild-type (*ETV6-WT*) and mutant *ETV6* harbouring deletion of the ETS domain (*ETV6-ΔETS*) were purchased from gBlock (IDT) and cloned into pDONR-221 via BP gateway cloning. Constructs were further cloned into pINDUCER20 (Addgene, 44012) by LR cloning and lentivirally packaged as described above. A673 and EW8 cells were transduced with lentivirus encoding either *ETV6-WT* or *ETV6-ΔETS* and incubated with 100 ng ml^–1^ of doxycycline or vehicle for 24 h. Subcellular fractionation was performed according to the manufacturer’s protocol (Thermo Fisher, PI78840). Western blotting and cell viability experiments were performed as described above.

### qPCR

Total RNA was extracted from cells using an extraction kit with column-based genomic DNA removal (Qiagen, 74134). RNA was reverse transcribed to cDNA using an iScript kit (Bio-Rad Laboratories, 1708841) and diluted 1:7 with H_2_O. For sgFLI rescue experiments, A673 ETV6–dTAG cells were transduced with sgChr2.2 or sgFLI CRISPR–Cas9 constructs and treated separately with DMSO or dTAG^V^-1 in duplicate. All qPCR reactions were performed using a TaqMan system (Thermo Fisher Scientific) with technical triplicates. Probes were selected to span exon–exon junctions when possible. Specific probes were as follows: *GAPDH*: Hs02758991_g1; *FAS*: Hs00236330_m1; *ACTA2*: Hs00426835_g1; *TRIB1*: Hs00179769_m1; *SEMA5B*: Hs00400720_m1; *BCL11B*: Hs01102259_m1; and *SOX11*: Hs00846583_s1. In each qPCR reaction, the gene of interest was measured using FAM dye, whereas *GAPDH* control was measured using VIC dye. Samples were analysed in 384-well plate format using 5 µl TaqMan gene expression master mix (Thermo Fisher Scientific, 4369016), 0.5 µl of FAM-emitting probe, 0.5 µl of VIC-emitting *GAPDH* probe and 4 µl of diluted cDNA for a total of 10 µl per reaction. qPCR plates were analysed using a QuantStudio 6Flex Real-Time PCR machine and the accompanying QuantStudio Real-Time PCR software v.1.7 (Thermo Fisher Scientific). The delta-threshold cycle number (ΔCt) was calculated as the difference in threshold cycle number (Ct) between the gene of interest and *GAPDH*. The ΔΔCt was calculated as the difference between the ΔCt of a particular sample and the average ΔCt of the DMSO-treated, sgChr2.2 control samples. Fold increase in gene expression (after the loss of ETV6) was calculated as the ratio of 2^−ΔΔCt^ in dTAG^V^-1-treated cells to the average 2^−ΔΔCt^ in DMSO-treated cells, in either the sgChr2.2 or the sgFLI conditions.

### RNA-seq

All RNA-seq experiments were performed using total RNA extracted using a column-based kit (Qiagen, 74104) and treated with DNAse digestion. The Life Technologies external RNA control consortium (ERCC) RNA spike-in samples were added to each sample for normalization per kit instructions (Thermo Fisher Scientific, 4456740). For all RNA-seq experiments, except the A673 sgETV6 CRISPR–Cas9 experiments, RNA-seq library preparation and sequencing were performed by Novogene (https://en.novogene.com) at a depth of roughly 20 million reads per sample. Per Novogene correspondence, the quality control for the RNA samples was performed using Qubit fluorometric quantitation (Thermo Fisher Scientific) and a Bioanalyzer instrument (Agilent). Libraries were then prepared using a New England Biolabs NEBNext Ultra II non-directional RNA Library Prep kit. Library quality and concentrations were assessed using Labchip (Perkin Elmer) and qPCR. Libraries were sequenced in 150-bp paired-end fashion on a Novaseq6000 instrument (Illumina). For the A673 sgETV6 CRISPR–Cas9 experiments, polyA-tailed mRNA was isolated from 1 μg total RNA using a magnetic bead-based kit per kit instructions (New England Biolabs, E7490S). RNA-seq library preparation was performed using a NEBNext Ultra II Directional RNA Library Prep Kit for Illumina (New England Biolabs, E7760S). Libraries were quantified using a Qubit dsDNA high sensitivity assay (Q32851). The distribution of DNA fragment sizes was measured using a High Sensitivity D1000 assay (Agilent, ScreenTape, 5067-5584; reagents, 5067-5585). The molarity of each library was calculated and normalized to 4 nM. Libraries were pooled and sequenced on a Nextseq 500 instrument (Illumina) (single-end; 75 cycles at a depth of roughly 40 million reads per sample) using a Nextseq 500 sequencing kit (Illumina, 20024906).

### CUT&Tag

CUT&Tag was performed as previously described^50^ with slight modifications by the Lessnick Laboratory (Nationwide Children’s Hospital, Columbus, OH). About 250,000 cells per CUT&Tag condition were bound to BioMag Plus Concanavalin A-coated magnetic beads (Bangs Laboratories, BP531) and incubated with primary antibodies (ETV6 rabbit, Bethyl A303-674, 1:50; ETV6 mouse, Sigma WH0002120M1, 1:50; rabbit anti-mouse, Abcam, ab46540, 1:50) overnight at 4 °C, and secondary antibodies (guinea pig anti-rabbit IgG, Antibodies-Online ABIN101961, 1:100; rabbit anti-mouse, Abcam ab46540, 1:100) for 1 h at room temperature. Adapter-loaded protein A–Tn5 fusion protein was added at a dilution of 1:250 and incubated for 1 h at room temperature. To activate Tn5, tagmentation buffer containing MgCl_2_ was added and samples were incubated for 1 h at 37 °C. Reactions were stopped by addition of EDTA, and DNA was solubilized with SDS and proteinase K for 1 h at 50 °C. Total DNA was purified using phenol–chloroform extraction followed by ethanol precipitation. CUT&Tag libraries were prepared using NEBNext HiFi 2× PCR master mix (NEB, M0541S) and indexed primers^97^ using a combined annealing–extension step at 63 °C for 10 s and 15 cycles followed by a 1.1× post-amplification AMPure XP (Beckman Coulter, A63880) bead clean-up. Libraries were pooled and sequenced (2 × 150 bp paired end) on an Illumina HiSeq4000 platform (Nationwide Children’s Hospital Institute for Genomic Medicine). Two independent replicates of each CUT&Tag sample were prepared.

### CUT&RUN

CUT and release using nuclease (CUT&RUN) was performed as previously described^98–100^ with slight modifications. In brief, 500,000 cells per condition were bound to activated ConA beads (EpiCypher 21-1401). Next, the ConA bead–cell mixture was resuspended in a cold antibody buffer and FLI-1-(ab133485; 1 μg per sample) antibody or 0.5 μg H3K4me3 (EpiCyper, 13-0041) as positive and 0.5 μg IgG (EpiCypher, 13-0042) as negative control were per sample added overnight. pAG-MNase (EpiCypher, 15-1016) was then added to each reaction to allow binding to the antibody-labelled chromatin. *E.* *coli* spike-in DNA (EpiCypher, 18-1401) was added following MNase activation. Subsequently, targeted chromatin was digested and released by the addition of CaCl_2_. The fragmented chromatin was purified using a CUTANA DNA Purification kit (EpiCypher, 14-0050). Quantification, library preparation and sequencing were performed by the genomics core at Dana-Farber Cancer Institute.

### ChIP-seq

Antibodies were conjugated to magnetic beads. For each immunoprecipitation (IP), 100 µl of protein A Dynabeads (Thermo Fisher Scientific, 10002D) were washed three times in 1 ml BSA blocking solution (0.5% w/v sterile-filtered BSA in H_2_O) and resuspended in 250 µl. Beads were then rotated overnight at 4 °C with antibody, using 5 µg to target H3K27ac (Abcam, 4729) or 10 µg to target TFs (anti-HA, Abcam, ab9110; anti-FLI1, Abcam, ab15289). For comparative studies (that is, comparing the relative binding of EWS–FLI), 2 µg of spike-in antibody recognizing a *Drosophila*-specific histone variant was added (Active Motif, 61686). The following morning, the antibody-conjugated beads were washed four times in 1 ml BSA blocking solution and then resuspended in 100 µl of the solution and stored at 4 °C.

To prepare sheared chromatin, Ewing sarcoma cells (20 million cells per ChIP reaction) were collected in a 1.5 ml tube and washed twice in 1 ml PBS. Cells were then crosslinked by resuspension in 1 ml PBS containing 1% methanol-free formaldehyde (Thermo Fisher Scientific, 28906) and rotated for 10 min at room temperature at 12 r.p.m. The reaction was quenched with 100 µl of 1.25 M glycine (Sigma Aldrich, G7126) and 100 µl 1 M Tris-HCl pH 8.0 (Thermo Fisher Scientific, 15568025). Cell pellets were washed twice with 1 ml PBS at room temperature and resuspended in 1 ml of SDS lysis buffer (0.5% SDS, 5 mM EDTA, 50 mM Tris-HCl pH 8.0) supplemented with protease inhibitor cocktail (Thermo Fisher Scientific, PI78429) and incubated at room temperature for 2 min with gentle agitation. Lysates were centrifuged at 15,000*g* for 10 min at 4 °C. The nuclear pellet was re-suspended in 950 µl of ChIP IP buffer (2 parts SDS lysis buffer and 1 part Triton dilution buffer, which was composed of 100 mM Tris-HCl pH 8.0, 100 mM NaCl, 5 mM EDTA, 0.2% NaN_3_ and 5% Triton X-100) supplemented with protease inhibitor and transferred to a milliTUBE (Covaris, 520130). Sonication was performed on an E220 Focus Ultra sonicator (Covaris) at 5% duty cycle, 140 W peak power, 200 cycles per burst, at 4 °C for 30 min per milliTUBE. Sheared chromatin was transferred to a 1.5 ml tube and centrifuged at 15,000*g* for 10 min at 4 °C. The supernatant of sheared chromatin was transferred to a new reaction tube. To prepare the ChIP DNA input sample, 5 µl of sheared chromatin was transferred to a PCR strip-tube and mixed with 40 µl de-crosslinking buffer (100 mM NaHCO_3_ and 1% SDS buffer), 1 µl RNAse A (Thermo Fisher Scientific, 12091021) and 1 µl proteinase K (Thermo Fisher Scientific, AM2546). The tube was incubated for 2 h at 65 °C in a thermal cycler to de-crosslink DNA–protein covalent bonds. DNA was isolated using Agencourt AMPure XP bead-based purification at a 1.2× ratio (Beckman Coulter, A63881), eluted in 50 µl H_2_O and stored at −20 °C. The remaining sheared chromatin was divided or pooled according to the target of interest; at least 5 million cells were used for IP of histone marks and 40 million cells for TFs. Each IP reaction was brought up to a total volume of at least 1 ml with ChIP IP buffer. Pooled reactions were conducted in 2 ml or 5 ml reaction tubes. 50 ng or 20 ng of *Drosophila* spike-in chromatin was added for each H3K27ac or TF ChIP reaction, respectively. The 100 µl conjugated bead–antibody solution was then added to the sheared chromatin. IP reactions were rotated overnight at 4 °C.

ChIP reactions were washed twice in 1 ml low-salt buffer (0.1% SDS, 1% Triton X-100, 2 mM EDTA, 20 mM Tris-HCl pH 8.0, and 150 mM NaCl), high-salt buffer (0.1% SDS, 1% Triton X-100, 2 mM EDTA, 20 mM Tris-HCl pH 8.0, and 500 mM NaCl), lithium chloride buffer (0.25 M LiCl, 1% IGEPAL-CH 630, 1% sodium deoxycholate, 10 mM Tris-HCl pH 8.0, and 1 mM EDTA) and then once in 700 µl Tris-EDTA buffer (Sigma Aldrich, 93283). Chromatin was eluted using 100 µl fresh ChIP elution buffer (1% SDS and 0.1 M NaHCO_3_) and rotated at room temperature for 15 min. Eluate was transferred to PCR tubes and mixed with 8 µl 2.5 M NaCl, 1 µl RNAse A and 1 µl proteinase K. Samples were de-crosslinked for 12–16 h at 65 °C on a thermal cycler. ChIP DNA was extracted from the de-crosslinked samples using AMPure XP beads at a 1.2× ratio and eluted in 20 µl of H_2_O. DNA was quantified using a Qubit dsDNA high sensitivity assay (Q32851). DNA fragment sizes were measured with a Tapestation instrument using a High Sensitivity D1000 assay (Agilent, ScreenTape, 5067-5584; reagents, 5067-5585).

ChIP-seq libraries were prepared using a SMARTer ThruPLEX single-index DNA-Seq kit (Takara Bio, R400674, R400695). H3K27ac and TF samples were PCR-amplified 4 and 10 cycles, respectively. Libraries were prepared as described above and sequenced in 37-bp paired-end fashion for 75 cycles (Illumina, 20024906) at a depth of roughly 30 million reads per sample on the NextSeq 500.

### ATAC-seq

A673 ETV6–dTAG cells were seeded and treated separately with DMSO or 1 µM dTAG^V^-1 for 72 h. ATAC-seq was performed as previously described^97^ on samples of 100,000 cells using a publicly available protocol (available at https://www.med.upenn.edu/kaestnerlab/assets/user-content/documents/ATAC-seq-Protocol-(Omni)-Kaestner-Lab.pdf) without modifications. The molarity of each library was calculated using a Qubit dsDNA Broad Range Assay kit (Thermo Fisher Scientific, Q32850) and a Tapestation D5000 dsDNA assay (Agilent; ScreenTape, 5067–5588; Ladder, 5067–5590; reagents, 5067–5589). Libraries were pooled and sequenced in 37-bp paired-end fashion for 75 cycles on an Illumina NextSeq 500 instrument.

### ChIP-seq data analysis

The raw Illumina sequencer output was converted to fastq format using the program bcl2fastq (v.2.17). Sequencing read quality was examined using FastQC (http://www.bioinformatics.babraham.ac.uk) (v.0.11.9). Trimming of low-quality reads and clipping of sequencing adapters was done using the program Trimmomatic (v.0.36)^101^, and all reads shorter than 40 bp after trimming were discarded. Reads were aligned to the human genome (hg19) using Bowtie2 (v.2.3.5)^102,103^ using the ‘—very_sensitive’ preset collection of parameters. File conversion of .bam to .sam was done using SamTools (v.1.9q)^104^, and duplicate reads were removed using Picard-tools (v.2.19.0) (http://picard.sourceforge.net). ChIP-seq peaks were called using MACS2 (ref. 105) with a false discovery rate (FDR) *q* < 0.01 unless otherwise stated. The MACS2 algorithm utilizes a dynamic Poisson distribution to capture local biases in the genomic sequence, which allows for a sensitive and robust prediction of peaks. Unless otherwise noted, peaks were assigned to the closest gene within ±400 kb using the ChIPseeker package in R^106^. Visualizations of the ChIP-seq data tracks were produced with the R Bioconductor Gviz package^107^.

### CUT&Tag data analysis

Quality control on raw sequencing reads were performed using FastQC (http://www.bioinformatics.babraham.ac.uk) (v.0.11.4). Adapter sequences and/or low-quality reads were trimmed using trim_galore (http://www.bioinformatics.babraham.ac.uk) (0.4.4_dev). Reads were aligned to human (hg19) and spike-in *E.* *coli* (Escherichia_coli_K_12_DH10B NCBI 2008-03-17) genomes using Bowtie2 (v.2.3.4.3)^102,103^ with the following options: --no-unal --no-mixed --no-discordant --dovetail --phred33 -q -I 10 -X 700. The option --very-sensitive was added when aligning to the spike-in genome. SamTools (v.1.9)^104^ was used to convert .sam to .bam with the ‘-bq 10’ option. Counts of mapped reads were spike-in normalized by calculating a scale factor using the ‘median ratio method’ from DESeq2. Spike-in normalization in conjunction with the median ratio method provide a robust normalization method to appropriately account for global changes of ETV6 occupancies^108,109^. Peaks in each biological replicate were called using MACS2 (v.2.2.7.1)^105^ with the spike-in normalization. All duplicate reads were kept in the analysis. To ensure reproducibility and consistency of peaks called across multiple biological replicates, we calculated irreproducibility discovery rate^109^ values and combined the replicates with rabbit anti-mouse as controls using DiffBind (v.2.14.0)^110^ and DESEq2 (v.1.26.0)^108^. To ensure high-quality peaks that are most likely to represent biological signals, the final peak lists were generated with following thresholds: irreproducibility discovery rate < 0.005, FDR < 0.05, log_2_(fold change) > 3 and mean normalized counts of ETV6 > 80 (Bethyl antibody) and >300 (Sigma antibody).

### CUT&RUN data analysis

CUT&RUN FLI1 data for the PEDS0009 sample used a pipeline based on the bulk-level method outlined in CUT&RUNTools 2.0 (ref. 111) that is largely the same as the ChIP-seq pipeline. The changes to the ChIP-seq pipeline are an extra adapter trimming step run after Trimmomatic using kseq from CUT&RUN Tools and the addition of the ‘—dovetail’ flag to the Bowtie2 command. CUT&RUN samples also included *E.* *coli* spike-in for sample normalization and it was aligned to the *E.* *coli* (Escherichia_coli_K_12_DH10B NCBI 2008-03-17) genome.

### Differential ChIP-seq binding

Differential binding analysis in ETV6–dTAG ChIP-seq samples was performed with the R Bioconductor package CSAW^89^. CSAW uses a sliding window approach to count reads across the genome from sorted and indexed .bam files, for which each window is tested for significant differences between libraries using statistical methods from the edgeR package. Differential CSAW analysis was performed on A673 and EW8 ETV6–dTAG at 6 and 72 h in FLI1 and H3K27ac. The differential analysis performed here normalized samples based on *Drosophila* spike-in values, the reads of which were aligned to the dm6 version of the *Drosophila* genome. The differential ChIP-seq analysis procedure generally followed the approach outlined in the CSAW introductory usage tutorial as follows. The .bam files were read in allowing a maximum fragment length of 800, a minimum *q* = 20 and discarding any reads that fell in the hg19 or dm6 ENCODE blacklist files. A window size of 150 bases was used for analysis and tiled across the genome in 50 base steps. The ChIP-seq input control samples were used to help filter out regions containing just background reads by binning input control reads into 10,000 base blocks with a threshold of minimum prior counts of 2. The binned input reads were then compared with the ChIP-seq binding across all regions, and all ChIP-seq regions with a fold change of less than 3 over input were filtered out. After filtering, adjacent and overlapping 150 base regions were merged together to reduce the number of hypotheses tested (for example, A673 6 h ETV6–dTAG FLI1 had an average merged window width of 494 bases). *Drosophila* spike-in control reads were processed similarly to the human reads except, as there was no input control for the spike-in control, the spike-in reads were filtered using a global filtering method that required regions to be threefold above background. The counts for all enriched spike-in regions were used to calculate the normalization factors by applying the trimmed mean of M-values method on these counts via the function normFactors. Differential binding is tested for significance using the quasi-likelihood framework in the edgeR package, whereby edgeR models the counts using a negative binomial distribution that accounts for over-dispersion between biological replicates. To account for multiple hypothesis testing, CSAW converts per-window statistics into a *P* value for each region and then applies the Benjamini–Hochberg method to calculate the corrected FDR.

### ChIP-seq heatmaps

ChIP-seq heatmaps were produced by functions in the following deeptools package (v.3.3.0)^112^: computeMatrix, plotProfile and plotHeatmap. All heatmaps were made using data in .bigWig files that have been generated by deeptools bamCompare that generates .bigWig files based on the comparison of a ChIP-seq sample .bam file to its corresponding input (from the same cell line and same batch) while being simultaneously normalized for sequencing depth. The function computeMatrix was then used to calculate scores for genome regions and to prepare an intermediate file that can be used with plotHeatmap and plotProfiles. Unless otherwise stated, the genome regions were regions defined by a BED file corresponding to ETV6 or FLI1 peaks. For Fig. 2a–c and Extended Data Fig. 3a–c, computeMatrix was used with multiple .bigWig score files and two BED region files, in which the ETV6 peaks are split into two groups depending on whether the ETV6 peak overlapped with a region defined by gene TSSs ± 2.5 kb according to UCSC hg19 refGene transcript definitions. Figure 2g,h used regions defined by differential FLI1 regions from *P* < 0.05 CSAW, whereby regions not intersecting with a TSS were further divided into two groups according to whether the region intersects with a H3K27ac ChIP-seq peak from MACS2 with *q* < 0.01 in the parental A673 or EW8 cell line.

### GGAA repeat frequency at peak locations

Stacked bar plots were created in R using frequencies of overlap from the function summarizePatternInPeaks from R Bioconductor package ChIPpeakAnno (v.3.9)^113^. The function summarizePatternInPeaks was used to calculate the frequency of overlap of regions of the standard hg19 reference genome with GGAA repeats (from a single GGAA up to five consecutive GGAA sequences without any gaps) with peaks in FLI1 and ETV6 as called by MACS2. The ENCODE datasets analysed were from the Gene Expression Omnibus: GSE96274 (B lymphocyte) and GSE95877 (K-562).

### Differential ATAC-seq regions

Processing of ATAC-seq data (that is, Fig. 4c) used the same pipeline as the ChIP-seq data, although an extra step was added after Bowtie2 alignment that used samtools to remove mitochondrial reads (ChrM). CSAW was used for the differential analysis of ATAC-seq data in the same manner as CSAW was used with ChIP-seq data, except that there was no input control for filtering or spike-in control for sample normalization. In the absence of a matching input control, CSAW region filtering was performed by requiring regions to be threefold above the local background, whereby local background was defined by using wider windowing function of 2,000 bases and requiring regions to be threefold above the neighbouring regions. Within CSAW, ATAC-seq samples were normalized to the background using 10,000 base windows to calculate compositional biases of samples.

### RNA-seq data analysis

Gene expression values were derived from paired-end RNA-seq data, except for the A673 sgETV6 CRISPR–Cas9 RNA-seq experiment, which was sequenced in single-end fashion. The RNA-seq processing pipeline was roughly modelled on the GTEx pipeline (https://github.com/broadinstitute/gtex-pipeline/)^114^. FastQC was used to evaluate read quality on raw RNA-seq reads. Reads were aligned to the human genome (hg19) using STAR^115^. Transcript-level quantifications were calculated using RSEM (v.1.3.1)^116^. Gene counts from STAR were then used to quantify differentially expressed genes between the experimental and control conditions using the R Bioconductor package DESeq2 (ref. 108) using the approximate posterior estimation for GLM coefficients (apeglm) method for effect size. Normalized expression values for individual samples were obtained from RSEM log_2_(TPM) values with the RSEM log_2_(TPM + 1) values used for GSEA and producing RNA-seq heatmap plots.

The RNA-seq samples included the ERCC spike-in control mix^117^. Sequences for the ERCC transcripts were added to the hg19 reference for the STAR transcript alignment, and the fold changes of ERCC probes were examined in the DESeq2 output. Fold changes for ERCC probes were typically very small between the conditions in the ETV6–dTAG sample sets (for example, average fold change for 24 h A673 ETV6–dTAG of 0.995 between conditions). As such, ERCC spike-ins were not used to perform sample normalization.

### Gene set pathway enrichment analysis

Gene set pathway enrichment analysis was performed with signatures from v.6.0 of the Broad Institute’s molecular signature database (MSigDB) (http://www.broadinstitute.org/gsea/msigdb/index.jsp) using the c2 curated gene sets from various sources such as online pathway databases, the biomedical literature and knowledge of domain experts. These pathway enrichment results are shown in Fig. 3f and Extended Data Fig. 4g. Pathway enrichment analysis was performed in R using the clusterProfiler package that provides the enricher function for a hypergeometric test for a test of over-representation of pathway genes in a set of user-defined genes. Figure 3f shows a combined enrichment plot of the top MSigDB c2 pathways enriched in the ETV6-repressed genes at 6, 24 and 72 h common to both A673 and EW8 (genes up in ETV6 dTAG^V^-1 treatment RNA-seq). The plot shows a selected subset of the top enriched c2 gene sets, and the complete set of enriched sets is shown Supplementary Tables 7–11. The dot size corresponds to the number of genes in the gene set out of the total number of significantly ETV6-repressed genes at 6, 24, and 72 h (85, 251 and 832 genes, respectively). The colour corresponds to the gene set grouping. Missing points at times along the *x* axis represent times at which the enrichment was not significant with *P* < 0.05. The pathways are ordered first by the gene group and then by the average gene ratio (count of repressed genes in a pathway/number of repressed genes) across the three time points. Extended Data Fig. 4g shows a combined enrichment plot of the top MSigDB c2 pathways enriched in the ETV6-activated genes at 6, 24 and 72 h common to both A673 and EW8 (genes down in ETV6 dTAG^V^-1 treatment RNA-seq). The plot shows a selected subset of the top enriched c2 gene sets, and the complete set of enriched sets is shown Supplementary Tables 12–17. The dot size corresponds to the number of genes in the gene set out of the total number of significantly ETV6-activated genes at 6, 24 and 72 h (33, 130 and 543 genes, respectively). The colour corresponds to the gene set grouping. Missing points at times along the *x* axis represent times at which the enrichment was not significant. The pathways are ordered first by the gene group and then by the average gene ratio (count of repressed genes in a pathway/number of repressed genes) across the three time points. Extended Data Fig. 7a shows a pie chart of the top 100 enriched c5 gene sets, ranked by significance, in A673 ETV6–dTAG cells at 24 h. Each c5 gene signature was assigned to one of the categories listed; a complete list is shown in Supplementary Table 19.

### GSEA

The GSEA algorithm^118,119^ was used to evaluate the association of gene sets with ETV6 regulation. GSEA was run with 2,500 permutations of the phenotype using signal-to-noise to rank genes. This GSEA algorithm was used for Fig. 3e to test enrichment and generate enrichment plots of ETV6-bound genes in ETV6-regulated genes. The A673 ETV6 peak locations are defined by the peaks that overlap in all three A673 ETV6 samples (two A673 ETV6 CUT&Tag samples from two ETV6 antibodies and one untreated A673 ETV6 dTAG HA sample) and the EW8 ETV6 peak locations are defined by peaks in the EW8 ETV6 HA sample. ETV6-bound genes were identified by mapping the peaks to their nearest genes using the R package ChIPseeker.

### Statistics and reproducibility

Further information is available in the Nature Portfolio Reporting Summary linked to this article. Figure panels displaying data from experiments with *n* = 1 include Figs. 2a–e, 4h, 5c–g and 6c,f,h and Extended Data Figs. 2c,e, 3a–d, 6d–g and 7g. Figure panels displaying data from experiments with *n* = 2 include Figs. 2f–i, 4a–c,e–g and 6a,b and Extended Data Figs. 3f,g and 5a,c,d. All other figure panels display data from experiments with at least *n* = 3.

### Reporting summary

Further information on research design is available in the Nature Portfolio Reporting Summary linked to this article.

## Online content

Any methods, additional references, Nature Portfolio reporting summaries, source data, extended data, supplementary information, acknowledgements, peer review information; details of author contributions and competing interests; and statements of data and code availability are available at 10.1038/s41556-022-01059-8.

## Supplementary information

Supplementary Fig. 1 Example of the flow gating strategy.

Reporting Summary

Supplementary Tables 1–20 File includes all Supplementary Tables referenced in the text. **Supplementary Tables 1–3**. **Tumour-type-specific dependencies and gene expression**. List of genes interrogated in the Pediatric Cancer DepMap CRISPR–Cas9 screen in Ewing sarcoma (Table 1), neuroblastoma (Table 2) and rhabdomyosarcoma (Table 3) and their expression in the Cancer Cell Line Encyclopedia (CCLE). The columns include gene name, effect size measuring the degree of dependency in the screen, *q* value of the comparison of dependency in each tumour type against all other tumour types in the screen and *q* value of the comparison of expression in each tumour type against all other tumour types in CCLE. **Supplementary Table 4**. **EWS–FLI-regulated genes do not consistently include** ***ETV6***. The columns list, from left to right, (1) the names of MSigDB c2 gene sets characterizing EWS–FLI-regulated genes, (2) the total number of genes in each gene set and (3) whether each gene set included the gene *ETV6*. **Supplementary Table 5**. **Expression of** ***ETV6***, ***BCL11B*** **and** ***ZEB2*** **in primary tumour samples**. List of primary tumour samples from the Treehouse Childhood Cancer Initiative and each sample’s expression of the genes *ETV6*, *BCL11B* and *ZEB2*. Samples are marked as being either Ewing sarcoma samples or other. **Supplementary Table 6.** **ETV6-repressed genes defined by dTAG at 6** **h**. List of genes that were significantly upregulated in RNA-seq at 6 h following treatment with 1 μM dTAG^V^-1, compared to DMSO, in both A673 ETV6–dTAG cells and EW8 ETV6–dTAG cells (DESeq2 Wald test Benjamini–Hochberg (BH) adjusted *P* value; *P* adjust < 0.05). The columns list the gene symbols and their DESeq2 base mean (mean normalized counts of all samples), DESeq2 log_2_(fold change) (shrunken apeglm method), DESeq2 standard error, DESeq2 Wald test *P* value and DESeq2 BH FDR-adjusted *P* value in both models. Genes are ranked by log_2_(fold change) in the A673 data. **Supplementary Table 7.** **ETV6-repressed genes defined by dTAG at 24** **h**. List of genes that were significantly upregulated in RNA-seq at 24 h following treatment with 1 μM dTAG^V^-1, compared to DMSO, in both A673 ETV6-dTAG cells and EW8 ETV6–dTAG cells (DESeq2 Wald test BH-adjusted *P* value; *P* adjust < 0.05). The columns list the gene symbols and their DESeq2 base mean (mean normalized counts of all samples), DESeq2 log_2_(fold change) (shrunken apeglm method), DESeq2 standard error, DESeq2 Wald test *P* value and DESeq2 BH FDR-adjusted *P* value in both models. Genes are ranked by log_2_(fold change) in the A673 data. **Supplementary Table 8.** **ETV6-repressed genes defined by dTAG at 72** **h**. List of genes that were significantly upregulated in RNA-seq at 72 h following treatment with 1 μM dTAG^V^-1, compared to DMSO, in both A673 ETV6–dTAG cells and EW8 ETV6–dTAG cells (DESeq2 Wald test BH-adjusted *P* value; *P* adjust < 0.05). The columns list the gene symbols and their DESeq2 base mean (mean normalized counts of all samples), DESeq2 log_2_(fold change) (shrunken apeglm method), DESeq2 standard error, DESeq2 Wald test *P* value and DESeq2 BH FDR-adjusted *P* value in both models. Genes are ranked by log_2_(fold change) in the A673 data. **Supplementary Table 9.** **MSigDB c2 curated gene sets enriched in 6** **h ETV6-repressed genes**. List of MSigDB c2 curated gene sets enriched in 6 h ETV6-repressed genes shown in Supplementary Table 6. Enrichment is determined by an over-representation analysis with *P* values based on the hypergeometric distribution (one-sided version of Fisher’s exact test). The columns list the Gene Set ID, gene ratio (fraction of repressed genes in set), bg ratio (background gene ratio), one-sided Fisher’s exact test *P* value, BH FDR-adjusted *P* value, *q* value and the number of genes scoring in each gene set. Gene sets are ranked by *q* value. **Supplementary Table 10.** **MSigDB c2 curated gene sets enriched in 24** **h ETV6-repressed genes**. List of MSigDB c2 curated gene sets enriched in 24 h ETV6-repressed genes shown in Supplementary Table 7. Enrichment is determined by an over-representation analysis with *P* values based on the hypergeometric distribution (one-sided version of Fisher’s exact test). The columns list the Gene Set ID, gene ratio (fraction of repressed genes in set), bg ratio (background gene ratio), one-sided Fisher’s exact test *P* value, BH FDR-adjusted *P* value, *q* value and the number of genes scoring in each gene set. Gene sets are ranked by *q* value. **Supplementary Table 11.** **MSigDB c2 curated gene sets enriched in 72** **h ETV6-repressed genes**. List of MSigDB c2 curated gene sets enriched in 72 h ETV6-repressed genes shown in Supplementary Table 8. Enrichment is determined by an over-representation analysis with *P* values based on the hypergeometric distribution (one-sided version of Fisher’s exact test). The columns list the Gene Set ID, gene ratio (fraction of repressed genes in set), bg ratio (background gene ratio), one-sided Fisher’s exact test *P* value, BH FDR-adjusted *P* value, *q* value and the number of genes scoring in each gene set. Gene sets are ranked by *q* value. **Supplementary Table 12.** **ETV6-activated genes defined by dTAG at 6** **h**. List of genes that were significantly downregulated in RNA-seq at 6 h following treatment with 1 μM dTAG^V^-1, compared to DMSO, in both A673 ETV6-dTAG cells and EW8 ETV6–dTAG cells (DESeq2 Wald test BH-adjusted *P* value; *P* adjust < 0.05). The columns list the gene symbols and their DESeq2 base mean (mean normalized counts of all samples), DESeq2 log_2_(fold change) (shrunken apeglm method), DESeq2 standard error, DESeq2 Wald test *P* value and DESeq2 BH FDR-adjusted *P* value in both models. Genes are ranked by log_2_(fold change) in the A673 data. **Supplementary Table 13.** **ETV6-activated genes defined by dTAG at 24** **h**. List of genes that were significantly downregulated in RNA-seq at 24 h following treatment with 1 μM dTAG^V^-1, compared to DMSO, in both A673 ETV6–dTAG cells and EW8 ETV6–dTAG cells (DESeq2 Wald test BH-adjusted *P* value; *P* adjust < 0.05). The columns list the gene symbols and their DESeq2 base mean (mean normalized counts of all samples), DESeq2 log_2_(fold change) (shrunken apeglm method), DESeq2 standard error, DESeq2 Wald test *P* value and DESeq2 BH FDR-adjusted *P* value in both models. Genes are ranked by log_2_(fold change) in the A673 data. **Supplementary Table 14.** **ETV6-activated genes defined by dTAG at 72** **h**. List of genes that were significantly downregulated in RNA-seq at 72 h following treatment with 1 μM dTAG^V^-1, compared to DMSO, in both A673 ETV6-dTAG cells and EW8 ETV6–dTAG cells (DESeq2 Wald test BH-adjusted *P* value; *P* adjust < 0.05). The columns list the gene symbols and their DESeq2 base mean (mean normalized counts of all samples), DESeq2 log_2_(fold change) (shrunken apeglm method), DESeq2 standard error, DESeq2 Wald test *P* value and DESeq2 BH FDR-adjusted *P* value in both models. Genes are ranked by log_2_(fold change) in the A673 data. **Supplementary Table 15.** **MSigDB c2 curated gene sets enriched in 6** **h ETV6-activated genes**. List of MSigDB c2 curated gene sets enriched in 6 h ETV6-activated genes shown in Supplementary Table 12. Enrichment is determined by an over-representation analysis with *P* values based on the hypergeometric distribution (one-sided version of Fisher’s exact test). The columns list the Gene Set ID, gene ratio (fraction of repressed genes in set), bg ratio (background gene ratio), one-sided Fisher’s exact test *P* value, BH FDR-adjusted *P* value, *q* value and the number of genes scoring in each gene set. Gene sets are ranked by *q* value. **Supplementary Table 16.** **MSigDB c2 curated gene sets enriched in 24** **h ETV6-activated genes**. List of MSigDB c2 curated gene sets enriched in 24 h ETV6-activated genes shown in Supplementary Table 13. Enrichment is determined by an over-representation analysis with *P* values based on the hypergeometric distribution (one-sided version of Fisher’s exact test). The columns list the Gene Set ID, gene ratio (fraction of repressed genes in set), bg ratio (background gene ratio), one-sided Fisher’s exact test *P* value, BH FDR-adjusted *P* value, *q* value and the number of genes scoring in each gene set. Gene sets are ranked by *q* value. **Supplementary Table 17.** **MSigDB c2 curated gene sets enriched in 72** **h ETV6-activated genes**. List of MSigDB c2 curated gene sets enriched in 72 h ETV6-activated genes shown in Supplementary Table 14. Enrichment is determined by an over-representation analysis with *P* values based on the hypergeometric distribution (one-sided version of Fisher’s exact test). The columns list the Gene Set ID, gene ratio (fraction of repressed genes in set), bg ratio (background gene ratio), one-sided Fisher’s exact test *P* value, BH FDR-adjusted *P* value, *q* value and the number of genes scoring in each gene set. Gene sets are ranked by *q* value. **Supplementary Table 18.** **MSigDB c5 gene ontology (GO) gene sets enriched in 24** **h ETV6-repressed genes**. List of MSigDB c5 curated gene sets enriched in 24 h ETV6-repressed genes shown in Supplementary Table 7. Enrichment is determined by an over-representation analysis with *P* values based on the hypergeometric distribution (one-sided version of Fisher’s exact test). The gene sets are ranked by *q* value. The columns list the rank, Gene Set ID, the assigned biological category (displayed in Extended Data Fig. 7a), the gene ratio (fraction of repressed genes in set), bg ratio (background gene ratio), one-sided Fisher’s exact test *P* value, BH FDR-adjusted *P* value, *q* value and the number of genes scoring in each dataset. **Supplementary Table 19.** **MSigDB c5 gene GO gene sets containing** ***SOX11*** **in ETV6-repressed genes**. The first, second and third columns list MSigDB c5 GO gene sets that contained the gene *SOX11* and were significantly enriched (one-sided Fisher’s exact test; *P* < 0.05) in 6 h, 24 h and 72 h ETV6-repressed genes, respectively. **Supplementary Table 20.** **Expression of ETS family TF genes after ETV6 loss**. RNA-seq expression and log_2_(fold change) of ETS family TF genes identified in Lambert et al., 2018 in A673 ETV6–dTAG and EW8 ETV6–dTAG cells at 6, 24 and 72 h following DMSO or 1 μM dTAG^V^-1 treatment. The columns list the gene symbols and their DESeq2 base mean (mean of normalized counts of all samples), DESeq2 log_2_(fold change) (shrunken apeglm method), DESeq2 Wald test *P* value and DESeq2 BH FDR-adjusted *P* value.

## Data Availability

CRISPR–Cas9 screen data and the genomic characterization of cancer cell lines (RNA-seq) used in this study are publicly available at https://depmap.org. Gene expression data from the Treehouse Childhood Cancer Initiative characterizing primary tumours is publicly available at https://treehousegenomics.soe.ucsc.edu/public-data/. The Broad Institute’s MSigDB is publicly available at http://www.broadinstitute.org/gsea/msigdb/index.jsp. Genomics data shown in this study have been deposited in the Gene Expression Omnibus under accession code GSE181554. Source data are provided with this paper.

## Source data

Source Data for Fig. 1 Statistical source data analysed in Fig. 1.
Source Data for Fig. 4 Statistical source data analysed in Fig. 4.
Source Data for Fig. 5 Statistical source data analysed in Fig. 5.
Source Data for Fig. 6 Statistical source data analysed in Fig. 6.
Extended Data Fig. 1 Statistical source data analysed in Extended Data Fig. 1.
Extended Data Fig. 2 Statistical source data analysed in Extended Data Fig. 2.
Extended Data Fig. 6 Statistical source data analysed in Extended Data Fig. 6.
Extended Data Fig. 7 Statistical source data analysed in Extended Data Fig. 7.
Fig. 1 raw western blots Unprocessed westerns shown in Fig. 1.
Fig. 4 raw western blots Unprocessed westerns shown in Fig. 4.
Fig. 6 raw western blots Unprocessed westerns shown in Fig. 6.
Extended Data Fig. 1 Unprocessed westerns shown in Extended Data Fig. 1.
Extended Data Fig. 2 Unprocessed westerns shown in Extended Data Fig. 2.
Extended Data Fig. 4 Unprocessed westerns shown in Extended Data Fig. 4.
Extended Data Fig. 6 Unprocessed westerns shown in Extended Data Fig. 6.
Extended Data Fig. 7 Unprocessed westerns shown in Extended Data Fig. 7.

## Code availability

No custom code was used in this study. Analysis was done with publicly available pipelines using approaches described in the Methods.

## References

[1] Tsherniak A (2017). Defining a cancer dependency map. Cell.

[2] Filbin M, Monje M (2019). Developmental origins and emerging therapeutic opportunities for childhood cancer. Nat. Med..

[3] Panditharatna E, Filbin MG (2020). The growing role of epigenetics in childhood cancers. Curr. Opin. Pediatr..

[4] Lawrence MS (2013). Mutational heterogeneity in cancer and the search for new cancer-associated genes. Nature.

[5] Gröbner SN (2018). The landscape of genomic alterations across childhood cancers. Nature.

[6] Jahangiri L (2020). Core regulatory circuitries in defining cancer cell identity across the malignant spectrum. Open Biol..

[7] Saint-André V (2016). Models of human core transcriptional regulatory circuitries. Genome Res..

[8] Ott CJ (2018). Enhancer architecture and essential core regulatory circuitry of chronic lymphocytic leukemia. Cancer Cell.

[9] Riddick G (2017). A core regulatory circuit in glioblastoma stem cells links MAPK activation to a transcriptional program of neural stem cell identity. Sci. Rep..

[10] Sanda T (2012). Core transcriptional regulatory circuit controlled by the TAL1 complex in human T cell acute lymphoblastic leukemia. Cancer Cell.

[11] Kron KJ (2017). TMPRSS2–ERG fusion co-opts master transcription factors and activates NOTCH signaling in primary prostate cancer. Nat. Genet..

[12] Bradner JE, Hnisz D, Young RA (2017). Transcriptional addiction in cancer. Cell.

[13] Chapuy B (2013). Discovery and characterization of super-enhancer-associated dependencies in diffuse large B cell lymphoma. Cancer Cell.

[14] Sengupta S, George RE (2017). Super-enhancer-driven transcriptional dependencies in cancer. Trends Cancer.

[15] Boeva V (2017). Heterogeneity of neuroblastoma cell identity defined by transcriptional circuitries. Nat. Genet..

[16] Durbin AD (2018). Selective gene dependencies in MYCN-amplified neuroblastoma include the core transcriptional regulatory circuitry. Nat. Genet..

[17] Gryder BE (2019). Histone hyperacetylation disrupts core gene regulatory architecture in rhabdomyosarcoma. Nat. Genet..

[18] Gryder BE (2017). PAX3–FOXO1 establishes myogenic super enhancers and confers BET bromodomain vulnerability. Cancer Discov..

[19] van Groningen T (2017). Neuroblastoma is composed of two super-enhancer-associated differentiation states. Nat. Genet..

[20] Grünewald TGP (2018). Ewing sarcoma. Nat. Rev. Dis. Prim..

[21] Delattre O (1992). Gene fusion with an ETS DNA-binding domain caused by chromosome translocation in human tumours. Nature.

[22] Toomey EC, Schiffman JD, Lessnick SL (2010). Recent advances in the molecular pathogenesis of Ewing’s sarcoma. Oncogene.

[23] Gangwal K, Close D, Enriquez CA, Hill CP, Lessnick SL (2010). Emergent properties of EWS/FLI regulation via GGAA microsatellites in Ewing’s sarcoma. Genes Cancer.

[24] Gangwal K (2008). Microsatellites as EWS/FLI response elements in Ewing’s sarcoma. Proc. Natl Acad. Sci. USA.

[25] Guillon N (2009). The oncogenic EWS–FLI1 protein binds in vivo GGAA microsatellite sequences with potential transcriptional activation function. PLoS ONE.

[26] Riggi N (2014). EWS–FLI1 utilizes divergent chromatin remodeling mechanisms to directly activate or repress enhancer elements in Ewing sarcoma. Cancer Cell.

[27] Boulay G (2017). Cancer-specific retargeting of BAF complexes by a prion-like domain. Cell.

[28] Boulay G (2018). Epigenome editing of microsatellite repeats defines tumor-specific enhancer functions and dependencies. Genes Dev..

[29] Riggi N, Suva ML, Stamenkovic I (2009). Ewing’s sarcoma origin: from duel to duality. Expert Rev. Anticancer Ther..

[30] Tanaka M (2014). Ewing’s sarcoma precursors are highly enriched in embryonic osteochondrogenic progenitors. J. Clin. Invest..

[31] von Levetzow C (2011). Modeling initiation of Ewing sarcoma in human neural crest cells. PLoS ONE.

[32] Cidre-Aranaz F, Alonso J (2015). EWS/FLI1 target genes and therapeutic opportunities in Ewing sarcoma. Front. Oncol..

[33] Fadul J (2015). EWS/FLI utilizes NKX2-2 to repress mesenchymal features of Ewing sarcoma. Genes Cancer.

[34] García-Aragoncillo E (2008). DAX1, a direct target of EWS/FLI1 oncoprotein, is a principal regulator of cell-cycle progression in Ewing’s tumor cells. Oncogene.

[35] Kinsey M, Smith R, Iyer AK, McCabe ERB, Lessnick SL (2009). EWS/FLI and its downstream target NR0B1 interact directly to modulate transcription and oncogenesis in Ewing’s sarcoma. Cancer Res..

[36] Wiles ET, Bell R, Thomas D, Beckerle M, Lessnick SL (2013). ZEB2 represses the epithelial phenotype and facilitates metastasis in Ewing sarcoma. Genes Cancer.

[37] Wiles ET, Lui-Sargent B, Bell R, Lessnick SL (2013). BCL11B is up-regulated by EWS/FLI and contributes to the transformed phenotype in Ewing sarcoma. PLoS ONE.

[38] Charville GW (2017). EWSR1 fusion proteins mediate PAX7 expression in Ewing sarcoma. Mod. Pathol..

[39] Smith R (2006). Expression profiling of EWS/FLI identifies NKX2.2 as a critical target gene in Ewing’s sarcoma. Cancer Cell.

[40] Shi X (2020). EWS–FLI1 regulates and cooperates with core regulatory circuitry in Ewing sarcoma. Nucleic Acids Res..

[41] Dharia NV (2021). A first-generation pediatric cancer dependency map. Nat. Genet..

[42] Brohl AS (2014). The genomic landscape of the Ewing sarcoma family of tumors reveals recurrent *STAG2* mutation. PLoS Genet..

[43] Crompton BD (2014). The genomic landscape of pediatric Ewing sarcoma. Cancer Discov..

[44] Tirode F (2014). Genomic landscape of Ewing sarcoma defines an aggressive subtype with co-association of *STAG2* and *TP53* mutations. Cancer Discov..

[45] Vaske OM (2019). Comparative tumor RNA sequencing analysis for difficult-to-treat pediatric and young adult patients with cancer. JAMA Netw. Open.

[46] Nabet B (2020). Rapid and direct control of target protein levels with VHL-recruiting dTAG molecules. Nat. Commun..

[47] Nabet B (2018). The dTAG system for immediate and target-specific protein degradation. Nat. Chem. Biol..

[48] Adane B (2021). STAG2 loss rewires oncogenic and developmental programs to promote metastasis in Ewing sarcoma. Cancer Cell.

[49] Park H, Seo Y, Kim JI, Kim W-J, Choe SY (2006). Identification of the nuclear localization motif in the ETV6 (TEL) protein. Cancer Genet. Cytogenet..

[50] Kaya-Okur HS (2019). CUT&Tag for efficient epigenomic profiling of small samples and single cells. Nat. Commun..

[51] Venkataraman A (2018). A toolbox of immunoprecipitation-grade monoclonal antibodies to human transcription factors. Nat. Methods.

[52] ENCODE Project Consortium. (2004). The ENCODE (ENCyclopedia Of DNA Elements) Project. Science.

[53] Rasighaemi P, Ward AC (2017). ETV6 and ETV7: siblings in hematopoiesis and its disruption in disease. Crit. Rev. Oncol. Hematol..

[54] Chakrabarti SR, Nucifora G (1999). The leukemia-associated gene *TEL* encodes a transcription repressor which associates with SMRT and mSin3A. Biochem. Biophys. Res. Commun..

[55] Irvin BJ (2003). TEL, a putative tumor suppressor, induces apoptosis and represses transcription of Bcl-XL. J. Biol. Chem..

[56] Kim CA (2001). Polymerization of the SAM domain of TEL in leukemogenesis and transcriptional repression. EMBO J..

[57] Lopez RG (1999). TEL is a sequence-specific transcriptional repressor. J. Biol. Chem..

[58] Wang LC (1997). Yolk sac angiogenic defect and intra-embryonic apoptosis in mice lacking the Ets-related factor TEL. EMBO J..

[59] Wang LC (1998). The *TEL/ETV6* gene is required specifically for hematopoiesis in the bone marrow. Genes Dev..

[60] Wang L, Hiebert SW (2001). TEL contacts multiple co-repressors and specifically associates with histone deacetylase-3. Oncogene.

[61] Guidez F (2000). Recruitment of the nuclear receptor corepressor N-CoR by the TEL moiety of the childhood leukemia-associated TEL-AML1 oncoprotein. Blood.

[62] Fenrick R (2000). TEL, a putative tumor suppressor, modulates cell growth and cell morphology of ras-transformed cells while repressing the transcription of stromelysin-1. Mol. Cell. Biol..

[63] Fisher MH (2020). *ETV6* germline mutations cause HDAC3/NCOR2 mislocalization and upregulation of interferon response genes. JCI Insight.

[64] Guenther LM (2019). A combination CDK4/6 and IGF1R inhibitor strategy for Ewing sarcoma. Clin. Cancer Res..

[65] García-Domínguez DJ (2018). The combination of epigenetic drugs SAHA and HCI-2509 synergistically inhibits EWS-FLI1 and tumor growth in Ewing sarcoma. Oncotarget.

[66] Seong BKA (2021). TRIM8 modulates the EWS/FLI oncoprotein to promote survival in Ewing sarcoma. Cancer Cell.

[67] Tsang SM, Oliemuller E, Howard BA (2020). Regulatory roles for SOX11 in development, stem cells and cancer. Semin. Cancer Biol..

[68] Yang Z (2019). SOX11: friend or foe in tumor prevention and carcinogenesis?. Ther. Adv. Med. Oncol..

[69] Beekman R, Amador V, Campo E (2018). SOX11, a key oncogenic factor in mantle cell lymphoma. Curr. Opin. Hematol..

[70] Shepherd JH (2016). The SOX11 transcription factor is a critical regulator of basal-like breast cancer growth, invasion, and basal-like gene expression. Oncotarget.

[71] Zvelebil M (2013). Embryonic mammary signature subsets are activated in *Brca1*^–/–^ and basal-like breast cancers. Breast Cancer Res..

[72] Yao Z (2015). The role of tumor suppressor gene SOX11 in prostate cancer. Tumour Biol..

[73] Hide T (2009). Sox11 prevents tumorigenesis of glioma-initiating cells by inducing neuronal differentiation. Cancer Res..

[74] Barretina J (2012). The Cancer Cell Line Encyclopedia enables predictive modelling of anticancer drug sensitivity. Nature.

[75] Liang H (1994). Solution structure of the *ets* domain of Fli-1 when bound to DNA. Nat. Struct. Biol..

[76] Boyer LA (2005). Core transcriptional regulatory circuitry in human embryonic stem cells. Cell.

[77] Boisclair Lachance JF, Webber JL, Hong L, Dinner AR, Rebay I (2018). Cooperative recruitment of Yan via a high-affinity ETS supersite organizes repression to confer specificity and robustness to cardiac cell fate specification. Genes Dev..

[78] Webber, J. L., Zhang, J., Massey, A., Sanchez-Luege, N. & Rebay, I. Collaborative repressive action of the antagonistic ETS transcription factors Pointed and Yan fine-tunes gene expression to confer robustness in *Drosophila*. *Development*10.1242/dev.165985 (2018). 10.1242/dev.165985 PMC6053666 29848501

[79] Webber JL, Zhang J, Mitchell-Dick A, Rebay I (2013). 3D chromatin interactions organize Yan chromatin occupancy and repression at the even-skipped locus. Genes Dev..

[80] Rebay I, Rubin GM (1995). Yan functions as a general inhibitor of differentiation and is negatively regulated by activation of the Ras1/MAPK pathway. Cell.

[81] O’Neill EM, Rebay I, Tjian R, Rubin GM (1994). The activities of two Ets-related transcription factors required for *Drosophila* eye development are modulated by the Ras/MAPK pathway. Cell.

[82] Zhang Y, Ho TD, Buchler NE, Gordân R (2021). Competition for DNA binding between paralogous transcription factors determines their genomic occupancy and regulatory functions. Genome Res..

[83] Sizemore GM, Pitarresi JR, Balakrishnan S, Ostrowski MC (2017). The ETS family of oncogenic transcription factors in solid tumours. Nat. Rev. Cancer.

[84] Lambert SA (2018). The human transcription factors. Cell.

[85] Kwiatkowski BA (1998). The ets family member Tel binds to the Fli-1 oncoprotein and inhibits its transcriptional activity. J. Biol. Chem..

[86] Raynaud S (1996). The 12;21 translocation involving TEL and deletion of the other TEL allele: two frequently associated alterations found in childhood acute lymphoblastic leukemia. Blood.

[87] Golub TR (1995). Fusion of the *TEL* gene on 12p13 to the *AML1* gene on 21q22 in acute lymphoblastic leukemia. Proc. Natl Acad. Sci. USA.

[88] Surdez D (2021). *STAG2* mutations alter CTCF-anchored loop extrusion, reduce *cis*-regulatory interactions and EWSR1–FLI1 activity in Ewing sarcoma. Cancer Cell.

[89] Lun AT, Smyth GK (2016). csaw: a Bioconductor package for differential binding analysis of ChIP-seq data using sliding windows. Nucleic Acids Res..

[90] Meyers RM (2017). Computational correction of copy number effect improves specificity of CRISPR–Cas9 essentiality screens in cancer cells. Nat. Genet..

[91] Khoogar R (2022). Single-cell RNA profiling identifies diverse cellular responses to EWSR1/FLI1 downregulation in Ewing sarcoma cells. Cell Oncol..

[92] Reynolds CP (1988). Biological classification of cell lines derived from human extra-cranial neural tumors. Prog. Clin. Biol. Res..

[93] Sanjana NE, Shalem O, Zhang F (2014). Improved vectors and genome-wide libraries for CRISPR screening. Nat. Methods.

[94] Price C (2019). Genome-wide interrogation of human cancers identifies EGLN1 dependency in clear cell ovarian cancers. Cancer Res..

[95] Malone CF (2021). Selective modulation of a pan-essential protein as a therapeutic strategy in cancer. Cancer Discov..

[96] Welford SM, Hebert SP, Deneen B, Arvand A, Denny CT (2001). DNA binding domain-independent pathways are involved in EWS/FLI1-mediated oncogenesis. J. Biol. Chem..

[97] Buenrostro JD, Wu B, Chang HY, Greenleaf WJ (2015). ATAC-seq: a method for assaying chromatin accessibility genome-wide. Curr. Protoc. Mol. Biol..

[98] Skene PJ, Henikoff JG, Henikoff S (2018). Targeted in situ genome-wide profiling with high efficiency for low cell numbers. Nat. Protoc..

[99] Skene PJ, Henikoff S (2017). An efficient targeted nuclease strategy for high-resolution mapping of DNA binding sites. eLife.

[100] Schmid M, Durussel T, Laemmli UK (2004). ChIC and ChEC; genomic mapping of chromatin proteins. Mol. Cell.

[101] Bolger AM, Lohse M, Usadel B (2014). Trimmomatic: a flexible trimmer for Illumina sequence data. Bioinformatics.

[102] Langmead B, Salzberg SL (2012). Fast gapped-read alignment with Bowtie 2. Nat. Methods.

[103] Langmead B, Wilks C, Antonescu V, Charles R (2019). Scaling read aligners to hundreds of threads on general-purpose processors. Bioinformatics.

[104] Li H (2009). The Sequence Alignment/Map format and SAMtools. Bioinformatics.

[105] Zhang Y (2008). Model-based analysis of ChIP-Seq (MACS). Genome Biol..

[106] Yu G, Wang LG, He QY (2015). ChIPseeker: an R/Bioconductor package for ChIP peak annotation, comparison and visualization. Bioinformatics.

[107] Hahne F, Ivanek R (2016). Visualizing genomic data using Gviz and Bioconductor. Methods Mol. Biol..

[108] Anders S, Huber W (2010). Differential expression analysis for sequence count data. Genome Biol..

[109] Chen K (2015). The overlooked fact: fundamental need for spike-in control for virtually all genome-wide analyses. Mol. Cell. Biol..

[110] Stark, R. & Brown, G. *DiffBind: Differential Binding Analysis of ChIP-Seq Peak Data* (Bioconductor, 2021).

[111] Yu F, Sankaran VG, Yuan G-C (2021). CUT&RUNTools 2.0: a pipeline for single-cell and bulk-level CUT&RUN and CUT&Tag data analysis. Bioinformatics.

[112] Ramírez F (2016). deepTools2: a next generation web server for deep-sequencing data analysis. Nucleic Acids Res..

[113] Zhu LJ (2010). ChIPpeakAnno: a Bioconductor package to annotate ChIP-seq and ChIP-chip data. BMC Bioinformatics.

[114] Battle A, Brown CD, Engelhardt BE, Montgomery SB (2017). Genetic effects on gene expression across human tissues. Nature.

[115] Dobin A (2013). STAR: ultrafast universal RNA-seq aligner. Bioinformatics.

[116] Li B, Dewey CN (2011). RSEM: accurate transcript quantification from RNA-Seq data with or without a reference genome. BMC Bioinformatics.

[117] Jiang L (2011). Synthetic spike-in standards for RNA-seq experiments. Genome Res..

[118] Mootha VK (2003). PGC-1α-responsive genes involved in oxidative phosphorylation are coordinately downregulated in human diabetes. Nat. Genet..

[119] Subramanian A (2005). Gene set enrichment analysis: a knowledge-based approach for interpreting genome-wide expression profiles. Proc. Natl Acad. Sci. USA.

