# Supplementary material for: The ETS transcription factor ETV6 constrains the transcriptional activity of EWS–FLI to promote Ewing sarcoma
Source: Nat Cell Biol. 2023 Jan 19;25(2):285–97. doi: 10.1038/s41556-022-01059-8 (PMC9928584; doi:10.1038/s41556-022-01059-8)

nature cell biology

Article

https://doi.org/10.1038/s41556-022-01059-8

The ETS transcription factor ETV6 constrains the transcriptional activity of EWS–FLI to promote Ewing sarcoma

In the format provided by the authors and unedited

Supplementary Figure 1

Sample of Flow Gating Strategy

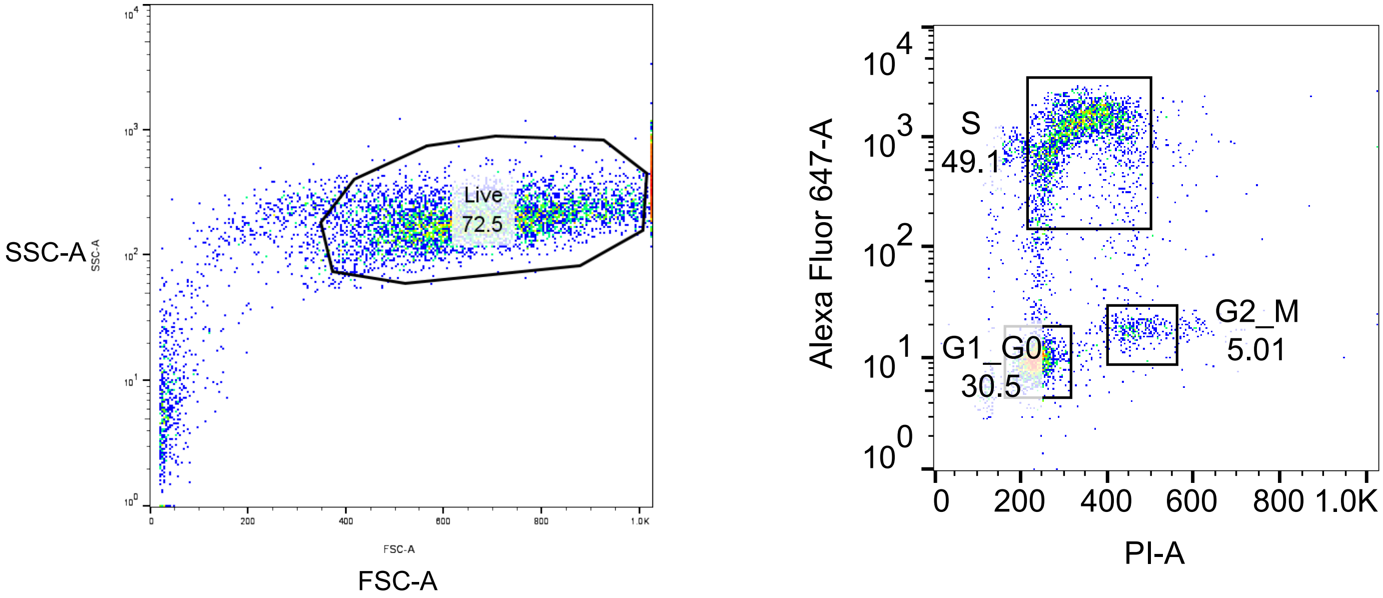

Supplement: Supplementary file 1 — Example of the flow gating strategy. [file 41556_2022_1059_MOESM1_ESM.pdf]
