# Supplementary material for: The ETS transcription factor ETV6 constrains the transcriptional activity of EWS–FLI to promote Ewing sarcoma
Source: Nat Cell Biol. 2023 Jan 19;25(2):285–97. doi: 10.1038/s41556-022-01059-8 (PMC9928584; doi:10.1038/s41556-022-01059-8)
Supplement: Supplementary file 2 — Reporting Summary [file 41556_2022_1059_MOESM2_ESM.pdf]

nature portfolio

Corresponding author(s): Kimberly Stegmaier, MD

Last updated by author(s): Nov 19, 2022

Reporting Summary

Nature Portfolio wishes to improve the reproducibility of the work that we publish. This form provides structure for consistency and transparency in reporting. For further information on Nature Portfolio policies, see our Editorial Policies and the Editorial Policy Checklist.

Statistics

For all statistical analyses, confirm that the following items are present in the figure legend, table legend, main text, or Methods section.

n/a	Confirmed	
☐	☒	The exact sample size (*n*) for each experimental group/condition, given as a discrete number and unit of measurement
☐	☒	A statement on whether measurements were taken from distinct samples or whether the same sample was measured repeatedly
☐	☒	The statistical test(s) used AND whether they are one- or two-sided<br>*Only common tests should be described solely by name; describe more complex techniques in the Methods section.*
☐	☒	A description of all covariates tested
☐	☒	A description of any assumptions or corrections, such as tests of normality and adjustment for multiple comparisons
☐	☒	A full description of the statistical parameters including central tendency (e.g. means) or other basic estimates (e.g. regression coefficient) AND variation (e.g. standard deviation) or associated estimates of uncertainty (e.g. confidence intervals)
☐	☒	For null hypothesis testing, the test statistic (e.g. *F*, *t*, *r*) with confidence intervals, effect sizes, degrees of freedom and *P* value noted<br>*Give P values as exact values whenever suitable.*
☒	☐	For Bayesian analysis, information on the choice of priors and Markov chain Monte Carlo settings
☒	☐	For hierarchical and complex designs, identification of the appropriate level for tests and full reporting of outcomes
☐	☒	Estimates of effect sizes (e.g. Cohen's *d*, Pearson's *r*), indicating how they were calculated

Our web collection on statistics for biologists contains articles on many of the points above.

Software and code

Policy information about availability of computer code

Data collection

No software was used for data collection.

Data analysis

Microsoft Excel Version 16.50
GraphPad Prism Version 9.0.0
FlowJo_v10.6.1
ImageQuant TL 8.2 (Cytiva)
ImageStudioLite 5.2.5
QuantStudio Real-Time PCR Software v1.7

ChIP-seq analysis: bcl2fastq (v2.17), FastQC (http://www.bioinformatics.babraham.ac.uk) (v0.11.9), Trimmomatic (v0.36), Bowtie2 (v2.3.5 and v2.3.4.3), SamTools (v1.9q), Picard-tools (v2.19.0), MACS2 (version 2.1.4), deeptools (v3.3.0)

RNA-seq analysis: FastQC (http://www.bioinformatics.babraham.ac.uk) (v0.11.9), STAR (v2.7.2b), RSEM (v1.3.1). The RNA-seq processing pipeline was roughly modeled on the GTEx pipeline (https://github.com/broadinstitute/gtex-pipeline/).

RStudio Version 1.2.5033
R version 4.0.4 (2021-02-15)
R packages: annotate_1.68.0, AnnotationDbi_1.52.0, askpass_1.1, assertthat_0.2.1, beeswarm_0.3.1, Biobase_2.50.0, BiocFileCache_1.14.0, BiocGenerics_0.36.0, BiocParallel_1.24.1, biomaRt_2.46.3, Biostrings_2.58.0, bit_4.0.4, bit64_4.0.5, bitops_1.0-6, blob_1.2.1, cachem_1.0.4, caTools_1.18.1, colorspace_2.0-0, compiler_4.0.4, cowplot_1.1.1, crayon_1.4.1, curl_4.3, data.table_1.14.0, DBI_1.1.1, dbplyr_2.1.0, DelayedArray_0.16.2, DESeq2_1.30.1, digest_0.6.27, DO.db_2.9, DOSE_3.16.0, dplyr_1.0.5, ellipsis_0.3.1, fansi_0.4.2, farver_2.1.0, fastmap_1.1.0, fastmatch_1.1-0, fgsea_1.16.0, genefilter_1.72.1, geneplotter_1.68.0, generics_0.1.0, GenomeInfoDb_1.26.4, GenomeInfoDbData_1.2.4, GenomicAlignments_1.26.0, GenomicFeatures_1.42.2, GenomicRanges_1.42.0, ggbeeswarm_0.6.0, ggplot2_3.3.3,

ggrepel_0.9.1, glue_1.4.2, GO.db_3.12.1, GOSemSim_2.16.1, gplots_3.1.1, graph_1.68.0, grid_4.0.4, gridExtra_2.3, GSEABase_1.52.1, gtable_0.3.0, gtools_3.8.2, hms_1.0.0, httr_1.4.2, IRanges_2.24.1, KernSmooth_2.23-18, lattice_0.20-41, lifecycle_1.0.0, locfit_1.5-9.4, magrittr_2.0.1, Matrix_1.3-2, MatrixGenerics_1.2.1, matrixStats_0.58.0, memoise_2.0.0, munsell_0.5.0, openssl_1.4.3, pheatmap_1.0.12, pillar_1.5.1, pkgconfig_2.0.3, plyr_1.8.6, prettyunits_1.1.1, progress_1.2.2, purrr_0.3.4, qvalue_2.22.0, R6_2.5.0, rappdirs_0.3.3, RColorBrewer_1.1-2, Rcpp_1.0.6, RCurl_1.98-1.3, reshape2_1.4.4, rlang_0.4.10, Rsamtools_2.6.0, RSQLite_2.2.4, rstudioapi_0.13, rtracklayer_1.50.0, S4Vectors_0.28.1scales_1.1.1, splines_4.0.4, stringi_1.5.3, stringr_1.4.0, SummarizedExperiment_1.20.0, survival_3.2-9, tibble_3.1.0, tidyselect_1.1.0, tools_4.0.4, utf8_1.2.1, vctrs_0.3.6, vipor_0.4.5, XML_3.99-0.6, xml2_1.3.2, xtable_1.8-4, XVector_0.30.0, zlibbioc_1.36.0

For manuscripts utilizing custom algorithms or software that are central to the research but not yet described in published literature, software must be made available to editors and reviewers. We strongly encourage code deposition in a community repository (e.g. GitHub). See the Nature Portfolio guidelines for submitting code & software for further information.

Data

Policy information about availability of data

All manuscripts must include a data availability statement. This statement should provide the following information, where applicable:

- Accession codes, unique identifiers, or web links for publicly available datasets
- A description of any restrictions on data availability
- For clinical datasets or third party data, please ensure that the statement adheres to our policy

CRISPR/Cas9 screen data and the genomic characterization of cancer cell lines (RNA-seq) used in this study are publicly available at depmap.org. Gene expression data from the Treehouse Childhood Cancer Initiative characterizing primary tumors is publicly available at https://treehousegenomics.soe.ucsc.edu/public-data/. Gene set pathway enrichment analysis was performed with signatures from version 6.0 of the Broad Institute's molecular signature database (MSigDB) (http://www.broadinstitute.org/gsea/msigdb/index.jsp). All genomics data is uploaded to Gene Expression Omnibus (GSE181554).

Field-specific reporting

Please select the one below that is the best fit for your research. If you are not sure, read the appropriate sections before making your selection.

☒ Life sciences ☐ Behavioural & social sciences ☐ Ecological, evolutionary & environmental sciences

For a reference copy of the document with all sections, see nature.com/documents/nr-reporting-summary-flat.pdf

Life sciences study design

All studies must disclose on these points even when the disclosure is negative.

Sample size	For CRISPR-Cas9 screening of cancer cell lines, no sample size was predetermined as the goal was to screen all possible human cancer cell lines. At this time, data for 796 human cancer cell lines were used. For other experiments, no statistical methods were used to pre-determine sample sizes, but our sample sizes are similar to those reported in previous publications. For all low-throughput validation experiments, studies were performed with a minimum of triplicate replicates. For the animal experiments, three to five animals were analyzed per condition based on lab experience. qPCR analysis was performed on two separate biological replicate cell samples, each analyzed in technical triplicate. Sample sizes are indicated in figure legends.
Data exclusions	From the DepMap analysis, 12 cell lines were not included in the subsequent analyses for the following reasons. Four cell lines are classified as engineered lines. The origin of one cell line, CHLA57, is unknown as it is incorrectly identified as an Ewing sarcoma line. Seven cell lines, including the Ewing sarcoma cell line, SKNMC, were removed because they were listed as commonly misidentified cell lines in the ICLAC Register of Misidentified Cell Lines (https://iclac.org/databases/cross-contaminations/). Therefore, dependency data for 796 cell lines were examined.
Replication	For CRISPR-Cas9 screening of cancer cell lines, replicate information is available at depmap.org. Figure legends indicate the nature of replicates shown as well as independently repeated experiments.
Randomization	Not applicable; animal studies did not involve drug treatment.
Blinding	Not applicable; animal studies did not involve drug treatment.

Reporting for specific materials, systems and methods

We require information from authors about some types of materials, experimental systems and methods used in many studies. Here, indicate whether each material, system or method listed is relevant to your study. If you are not sure if a list item applies to your research, read the appropriate section before selecting a response.

Materials & experimental systems

n/a	Involved in the study
☐	☒ Antibodies
☐	☒ Eukaryotic cell lines
☒	☐ Palaeontology and archaeology
☐	☒ Animals and other organisms
☒	☐ Human research participants
☒	☐ Clinical data
☒	☐ Dual use research of concern

Methods

n/a	Involved in the study
☐	☒ ChIP-seq
☐	☒ Flow cytometry
☒	☐ MRI-based neuroimaging

Antibodies

Antibodies used

Anti-GAPDH (#2118S, rabbit, monoclonal, clone 14C10, Cell Signaling Technology)
Anti-ETV6 (#WH0002120M1-100UG, mouse, monoclonal, clone 3B10, Sigma Aldrich; #SC-166835, mouse, monoclonal, clone E-1, Santa Cruz Biotechnology; #A303-674, rabbit, polyclonal, Bethyl)
Anti-H3K27ac (#ab4729, rabbit, polyclonal, Abcam)
Anti-HA (#3724S, rabbit, monoclonal, clone C29F4, Cell Signaling Technology)
Anti-PARP (#9542S, rabbit, polyclonal, Cell Signaling Technology)
Anti-cleaved Caspase-3 (#9664S, rabbit, monoclonal, clone 5A1E, Cell Signaling Technology)
Anti-FLI1 (#ab15289, rabbit, polyclonal, Abcam)
Anti-FAS (#SC-8009, mouse, monoclonal, clone B-10, Santa Cruz Biotechnology)
Anti-SEMA5B (#PA5113369, rabbit, polyclonal, Thermo Fisher Scientific)
Anti-BCL11B (#12120S, rabbit, monoclonal, clone D6F1, Cell Signaling Technology)
Anti-SOX11 (# 58207S, rabbit, monoclonal, clone E7G9N, Cell Signaling Technology)
Horseradish peroxidase anti-mouse (Cell Signaling Technology, #7076S)
Horseradish peroxidase anti-rabbit (Cell Signaling Technology, #7074S)
IRDye anti-mouse (LICOR Biosciences, #926-32210)
IRDye anti-rabbit (LICOR Biosciences, #926-68071)

Validation

All antibodies were reportedly validated by the manufacturer. Anti-PARP and anti-cleaved Caspase-3 were validated by the manufacturer by treating cells with DNA-damaging agents. Similarly, in this study, cisplatin-treated cells were used as a positive control validating these antibodies (ED Fig 2c). Antibodies validated by target knock-out and western blot with data shown in this paper include anti-FLI1 (Fig 4h; ED Fig 6a/b/c), anti-SOX11 (Fig 6d; ED Fig 7d). Anti-ETV6 was validated in the same manner (Fig 1b/e/h; ED Fig 1f; ED Fig 2b; ED Fig 4a/d; ED Fig 6a/b/c; ED Fig 7g) as well as by CUT&Run (ED Fig 6d). Anti-HA was validated by western blot and ChIP-seq comparing DMSO vs. dTAG-treated cells in ETV6-dTAG cells (eg, Fig 1e, ED Fig 1h, ED Fig 3b). Additionally, we validated antibodies for the following proteins by knock-out and western blot, though this data is not shown in this paper: BCL11B, SEMA5B, FAS.

Eukaryotic cell lines

Policy information about cell lines

Cell line source(s)

All cell lines used for the genome-scale CRISPR-Cas9 screen are detailed in Meyers RM et al., Nature Genetics 2017, and on depmap.org. A673 cells were purchased from ATCC (CRL-1598). EW8 and TC32 were a kind gift from the Golub Lab (Broad Institute). EW8 cells were originally established in the Houghton Lab (Greehey Children's Cancer Research Institute, San Antonio, TX). TC-32 cells were originally obtained from Children's Oncology Group (COG) Childhood Cancer Repository. The PEDS0009 and PEDS0010 cell lines were obtained from the Cancer Cell Line Factory (Broad Institute, Cambridge, MA). NO human studies were performed. In Extended Data Fig. 6c, a minimally passaged Ewing sarcoma cell line previously derived in our laboratory (Seong et al., 2021) from a previously characterized patient-derived xenograft (PDX) (Garcia-Dominguez et al., 2018) (HSJD-ES-PDX-001) was studied. (As such, this experiment was performed in vitro and did not involve the use of animals.) As previously described (Garcia-Dominguez et al., 2018), this PDX originated from a scapular biopsy in a 21.7 year-old patient whose sex was not reported. It was collected with informed consent without compensation under an Institutional Review Board-approved protocol at Sant Joan de Déu Hospital (HSJD, Barcelona, Spain), animal protocol number HSJD 135/1165, and provided to our laboratory by Dr. Jaume Mora. The Rhabdomyosarcoma RD cell line was a gift from the DepMap group at the Broad Institute and was originally obtained from ATCC (catalog no. CRL-7731) .

Authentication

All cell lines used were STR tested for identity at either the Dana-Farber Cancer Institute molecular diagnostics core facility or The Broad Institute of MIT and Harvard.

Mycoplasma contamination

All cells were tested for mycoplasma and confirmed negative.

Commonly misidentified lines
(See ICLAC register)

All ICLAC lines were removed from the analysis of the DepMap CRISPR/Cas9 dependency data, including the Ewing sarcoma cell line, SKNMC.

Animals and other organisms

Policy information about studies involving animals; ARRIVE guidelines recommended for reporting animal research

Laboratory animals	The study shown in Fig. h/i used 7 week-old female NOD.Cg-Prkdcscid Il2rgtm1Wjl/SzJ (NSG) mice ordered from Jackson Laboratory. The study shown in Extended Data Fig. 2d used 12 week-old male NOD.Cg-Prkdcscid Il2rgtm1Wjl/SzJ (NSG) mice ordered from Jackson Laboratory.<br>The study shown in Fig. 6h used 6-8 week-old female CrTac:NCR-Foxn1<nu> (nude) mice from Taconic Biosciences.<br>Mice were housed at Dana-Farber Cancer Institute with regulated temperature and humidity and standard 12 hour light/dark cycles.
Wild animals	No wild animals were used.
Field-collected samples	No field-collected samples were used.
Ethics oversight	All of our mouse studies were approved by the DFCI IACUC committee and were performed in accordance with NIH guidelines for the humane care and use of animals. Animal Welfare Assurance Number: D16-00010 (A3023-01).

Note that full information on the approval of the study protocol must also be provided in the manuscript.

ChIP-seq

Data deposition

☒ Confirm that both raw and final processed data have been deposited in a public database such as GEO.

☒ Confirm that you have deposited or provided access to graph files (e.g. BED files) for the called peaks.

Data access links<br>*May remain private before publication.*	We have deposited all new genomics data to GEO (GSE181554) for publication.
Files in database submission	GSM5505948<br>GSM5505949<br>GSM5505950<br>GSM5505951<br>GSM5505952<br>GSM5505953<br>GSM5505954<br>GSM5505955<br>GSM5505956<br>GSM5505957<br>GSM5505958<br>GSM5505959<br>GSM5505960<br>GSM5505961<br>GSM5505962<br>GSM5505963<br>GSM5505964<br>GSM5505965<br>GSM5505966<br>GSM5505967<br>GSM5505968<br>GSM5505969<br>GSM5505970<br>GSM5505971<br>GSM5505972<br>GSM5505973<br>GSM5505974<br>GSM5505975<br>GSM5505976<br>GSM5505977<br>GSM5505978<br>GSM5505979<br>GSM5505980<br>GSM5505981<br>GSM5505982<br>GSM5505983<br>GSM5505984<br>GSM5505985<br>GSM5505986<br>GSM5505987<br>GSM5505988<br>GSM5505989<br>GSM5505990<br>GSM5505991

GSM5505992
GSM5505993
GSM5505994
GSM5505995
GSM5505996
GSM5505997
GSM5505998
GSM5505999
GSM5506000
GSM5506001
GSM5506002
GSM5506003
GSM5506004
GSM5506005
GSM5506006
GSM5506007
GSM5506008
GSM5506009
GSM5506010
GSM5506011
GSM5506012
GSM5506013
GSM5506014
GSM5506015
GSM5506016
GSM5506017
GSM5506018
GSM5506019
GSM5506020
GSM5506021
GSM5506022
GSM5506023
GSM5506024
GSM5506025
GSM5506026
GSM5506027
GSM5506028
GSM5506029
GSM5506030
GSM5506031
GSM5506032
GSM5506033
GSM5506034
GSM5506035
GSM5506036
GSM5506037
GSM5506038
GSM5506039
GSM5506040
GSM5506041
GSM5506042
GSM5506043
GSM5506044
GSM5506045
GSM5775771
GSM5775772
GSM5775773
GSM5775774
GSM5775775
GSM5775776
GSM5775777
GSM5775778
GSM5775779
GSM5775780
GSM5775781
GSM5775782
GSM5775783
GSM5775784
GSM5775785
GSM5775786
GSM5775787
GSM5775788
GSM6503406
GSM6503407
GSM6503408
GSM6503409

GSM6503410
GSM6503411
GSM6503412
GSM6503413
GSM6503414
GSM6503415
GSM6503416
GSM6503417
GSM6503418
GSM6503419

Genome browser session (e.g. UCSC)

Not applicable.

Methodology

Replicates

At least 2 replicates were used for all ChIP-seq experiments.

Sequencing depth

All sequencing was done to a minimum depth of 20 million reads per sample.

Antibodies

ChIP-seq primary antibodies:
Anti-H3K27ac (Abcam, # 4729)
Anti-HA (Abcam, # ab9110)
Anti-FLI1 (Abcam, # ab15289)
Spike-in Anti-Drosophila histone-variant (Active Motif, # 61686)

Peak calling parameters

ChIP-seq peaks were called using MACS2 with the False Discovery Rates (FDR) q < 0.01 unless otherwise stated.

Data quality

All ChIP-seq experiments were performed in duplicate to ensure rigor. The raw Illumina sequencer output was converted to FASTQ format using the program bcl2fastq (v2.17). Sequencing read quality was examined using FastQC (http://www.bioinformatics.babraham.ac.uk) (v0.11.9). Trimming of low-quality reads and clipping of sequencing adapters was done using the program Trimmomatic (v0.36)77 and all reads shorter than 40bp after trimming were dropped. Reads were aligned to the human genome (hg19) using Bowtie2 (v2.3.5)78,79 using the '—very_sensitive' preset collection of parameters. Bam to Sam file conversion was done with SamTools (v1.9q)80 and duplicate reads were removed using Picard-tools (v2.19.0) (http://picard.sourceforge.net). ChIP-seq peaks were called using MACS281 with the False Discovery Rates (FDR) q < 0.01 unless otherwise stated. The MACS2 algorithm utilizes a dynamic Poisson distribution to capture local biases in the genomic sequence, which allows for a sensitive and robust prediction of peaks. Unless otherwise noted, peaks were assigned to the closest gene within +/- 400kb using the ChIPseeker package in R82. Visualizations of the ChIP-seq data tracks were produced with the R Bioconductor Gviz package83.

Software

All ChIP-seq experiments were performed in duplicate to ensure rigor. The raw Illumina sequencer output was converted to FASTQ format using the program bcl2fastq (v2.17). Sequencing read quality was examined using FastQC (http://www.bioinformatics.babraham.ac.uk) (v0.11.9). Trimming of low-quality reads and clipping of sequencing adapters was done using the program Trimmomatic (v0.36)77 and all reads shorter than 40bp after trimming were dropped. Reads were aligned to the human genome (hg19) using Bowtie2 (v2.3.5)78,79 using the '—very_sensitive' preset collection of parameters. Bam to Sam file conversion was done with SamTools (v1.9q)80 and duplicate reads were removed using Picard-tools (v2.19.0) (http://picard.sourceforge.net). ChIP-seq peaks were called using MACS281 with the False Discovery Rates (FDR) q < 0.01 unless otherwise stated. The MACS2 algorithm utilizes a dynamic Poisson distribution to capture local biases in the genomic sequence, which allows for a sensitive and robust prediction of peaks. Unless otherwise noted, peaks were assigned to the closest gene within +/- 400kb using the ChIPseeker package in R82. Visualizations of the ChIP-seq data tracks were produced with the R Bioconductor Gviz package83.

Flow Cytometry

Plots

Confirm that:

☒ The axis labels state the marker and fluorochrome used (e.g. CD4-FITC).

☒ The axis scales are clearly visible. Include numbers along axes only for bottom left plot of group (a 'group' is an analysis of identical markers).

☒ All plots are contour plots with outliers or pseudocolor plots.

☒ A numerical value for number of cells or percentage (with statistics) is provided.

Methodology

Sample preparation

Cell cycle analysis was performed using the Click-iT™ Plus EdU Alexa Fluor™ 647 Flow Cytometry Assay Kit (Life Technologies, #C10424). Cells were analyzed per kit instructions with minor modifications. Cells were pulsed with 10 µM of the modified nucleotide analogue, EdU (5-ethynyl-2'-deoxyuridine), by spiking the molecule provided at stock concentration (10 mM) into the existing media for 90 minutes in a tissue culture incubator. According to kit instructions, 1-2 million cells per sample were trypsinized, washed, fixed, permeabilized, and then treated with a reaction cocktail containing Alexa Fluor-647-conjugated picolyl azide to label incorporated EdU. Following this step, cells were washed and then stained with an RNAse-containing propidium iodide solution (Cell Signaling, #4087S) for 45 minutes in a tissue culture incubator. Lastly, cells were analyzed by flow cytometry at 5,000-10,000 cells per sample. Live cells were gated using FSC-A and SSC-A. dTAG cell samples were seeded in triplicate and treated separately with DMSO or dTAGV-1 prior to EdU pulsing. A673 ETV6 knock-out cells were

	seeded and cultured separately prior to EdU pulsing. In all experiments, additional cells were collected from each sample for western blot validation.
Instrument	BD FacsCelesta
Software	FlowJo_v10.6.1
Cell population abundance	This experiment did not involve sorting.
Gating strategy	A figure exemplifying gating strategy has been provided separately and demonstrates the following: Live cells were gated using FSC-A and SSC-A. G1/G0 cells were gated as cells exhibiting relatively less propidium iodide and less Alexa Fluor-647 staining. G2/M cells were gated as cells exhibiting relatively high propidium iodide and less AF-647 staining. S cells were gated as cells exhibiting relatively high AF-647 and intermediate propidium iodide staining.

☒ Tick this box to confirm that a figure exemplifying the gating strategy is provided in the Supplementary Information.
