# Supplementary figures and images for: The ETS transcription factor ETV6 constrains the transcriptional activity of EWS–FLI to promote Ewing sarcoma
Source: Nat Cell Biol. 2023 Jan 19;25(2):285–97. doi: 10.1038/s41556-022-01059-8 (PMC9928584; doi:10.1038/s41556-022-01059-8)

**Figure 1b**

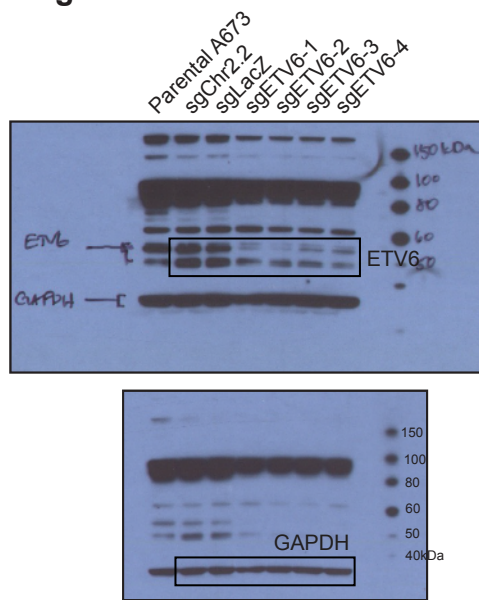

**Figure 1e**

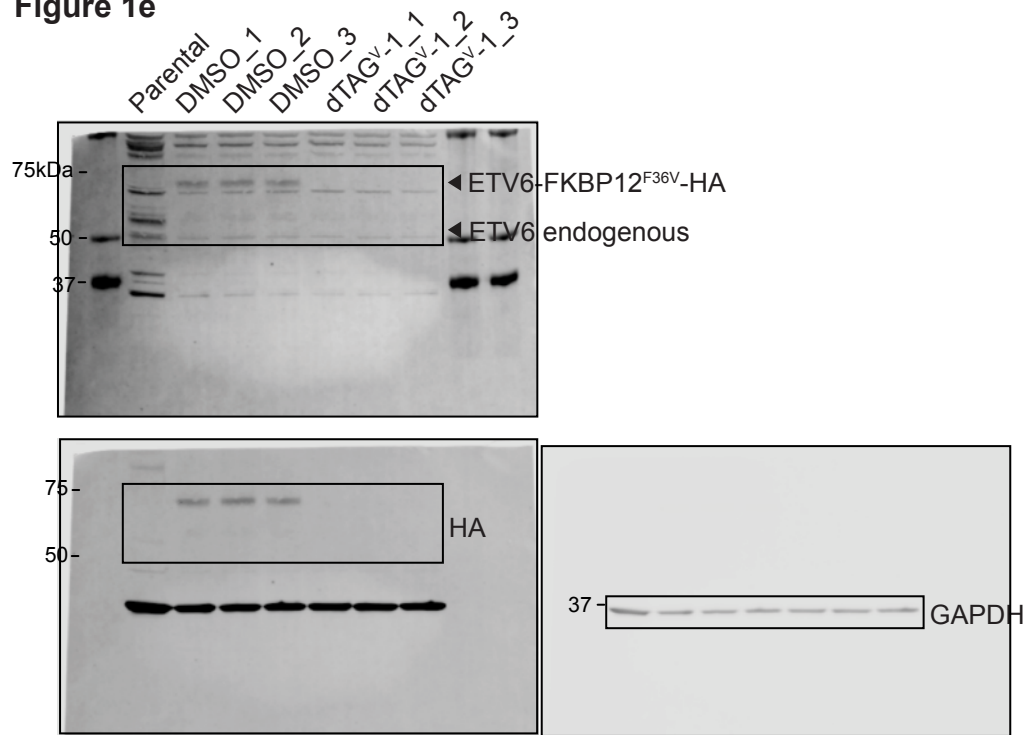

**Figure 1h**

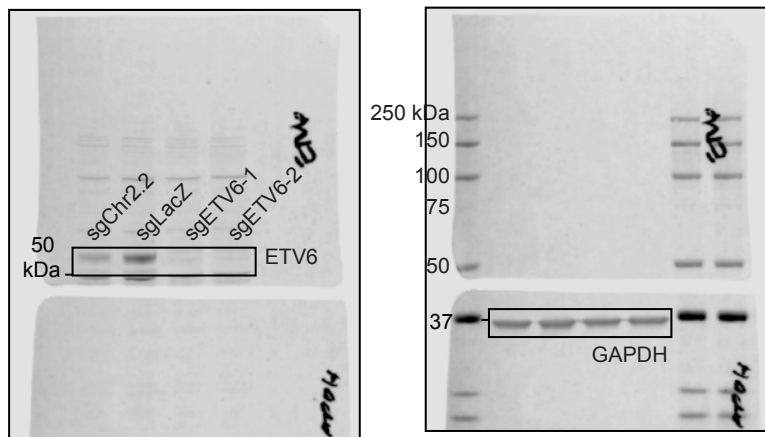

Supplement: Fig. 1 raw western blots — Unprocessed westerns shown in Fig. 1. [file 41556_2022_1059_MOESM12_ESM.pdf]

Figure 4h

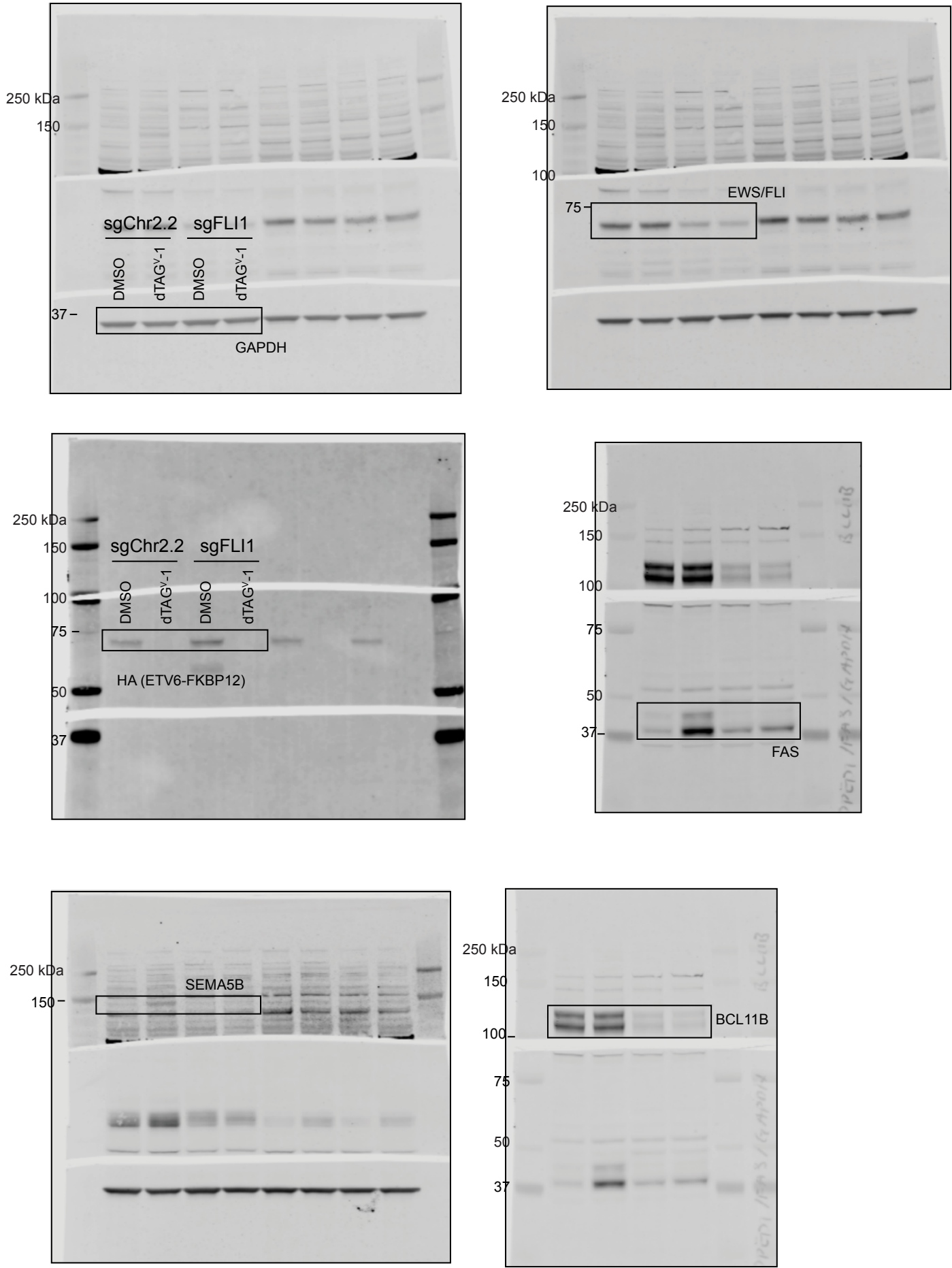

Supplement: Fig. 4 raw western blots — Unprocessed westerns shown in Fig. 4. [file 41556_2022_1059_MOESM13_ESM.pdf]

Figure 6c

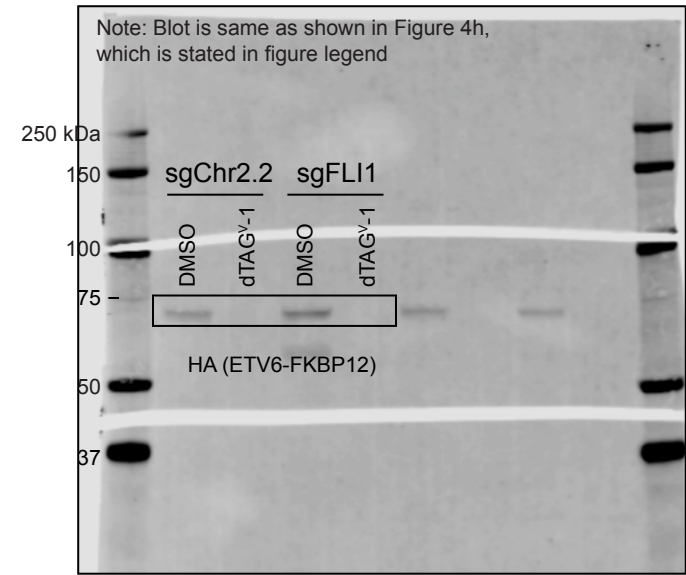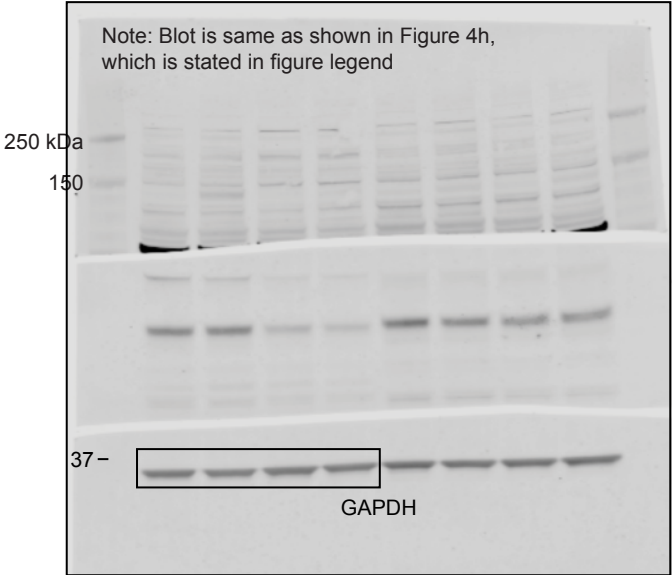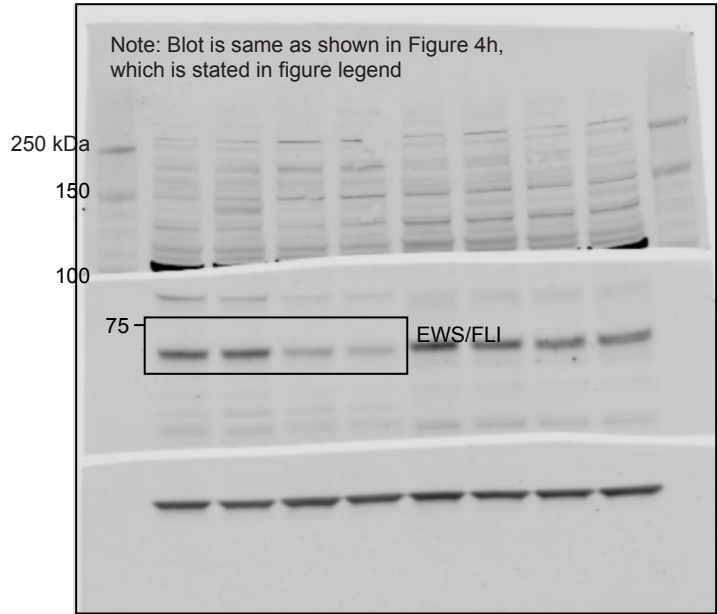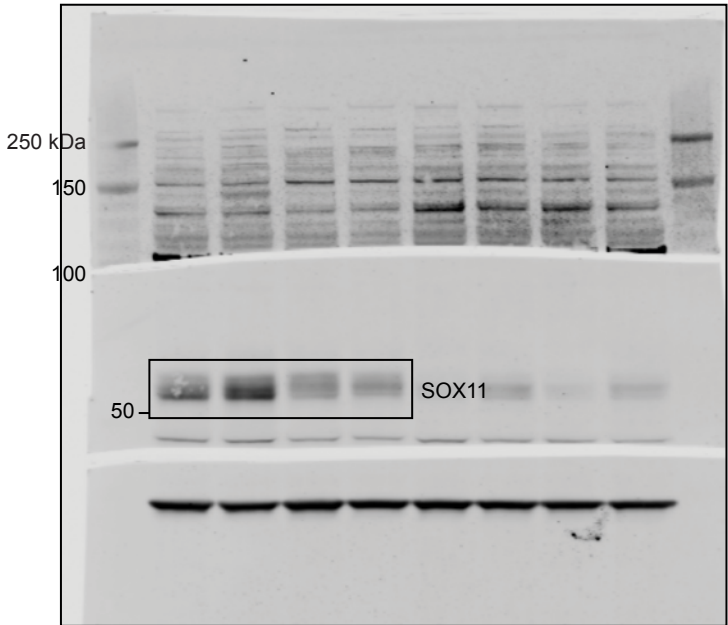

Figure 6c, continued

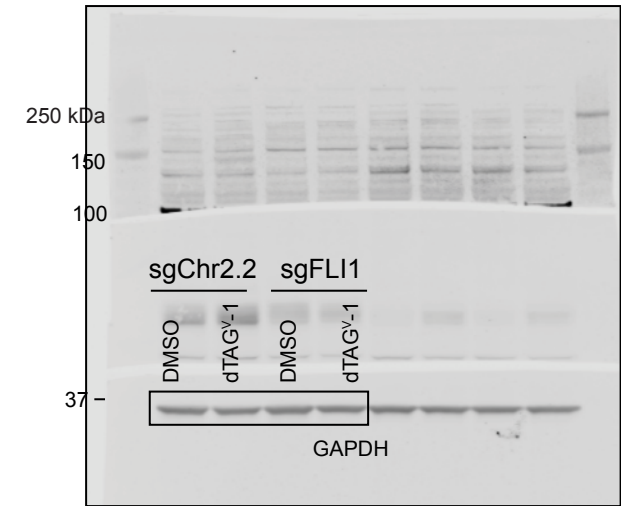

Figure 6d

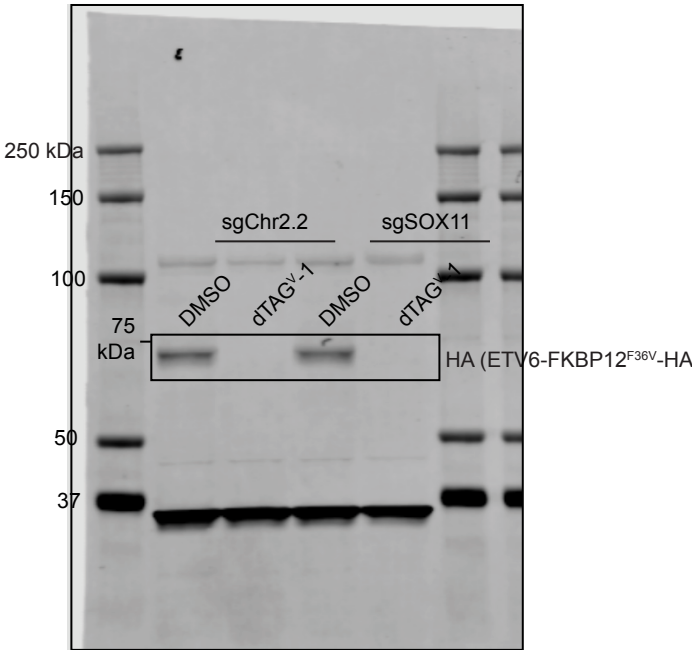

Figure 6d, continued

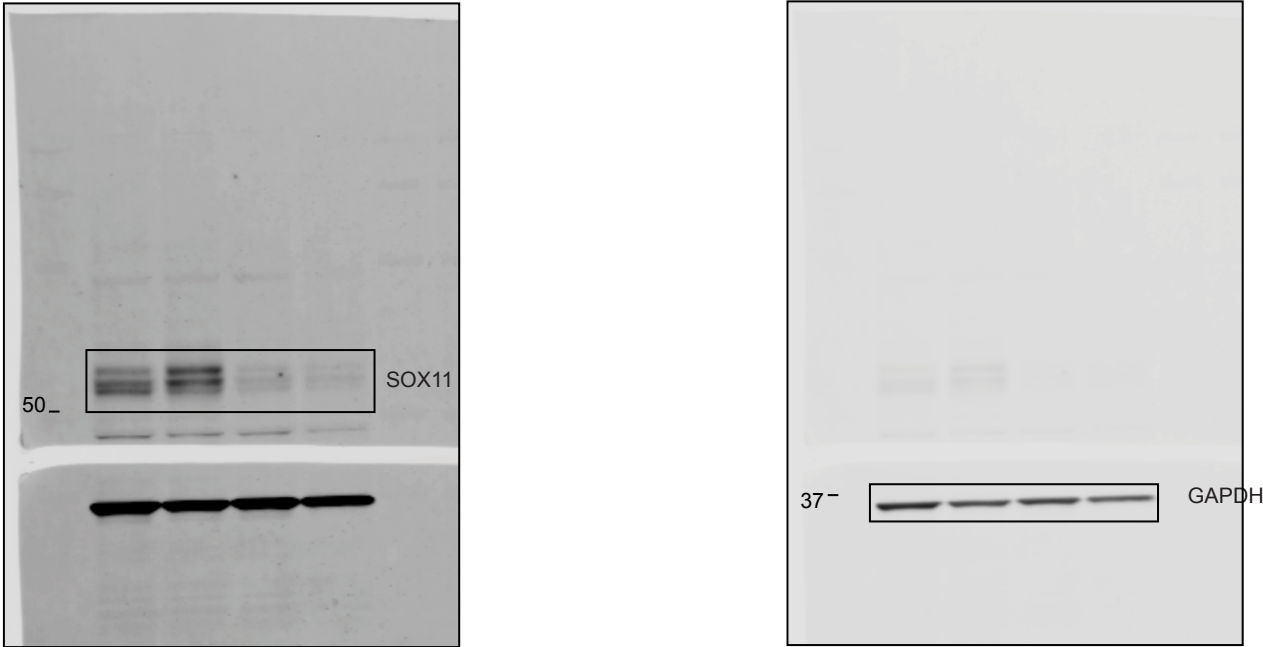

Figure 6f

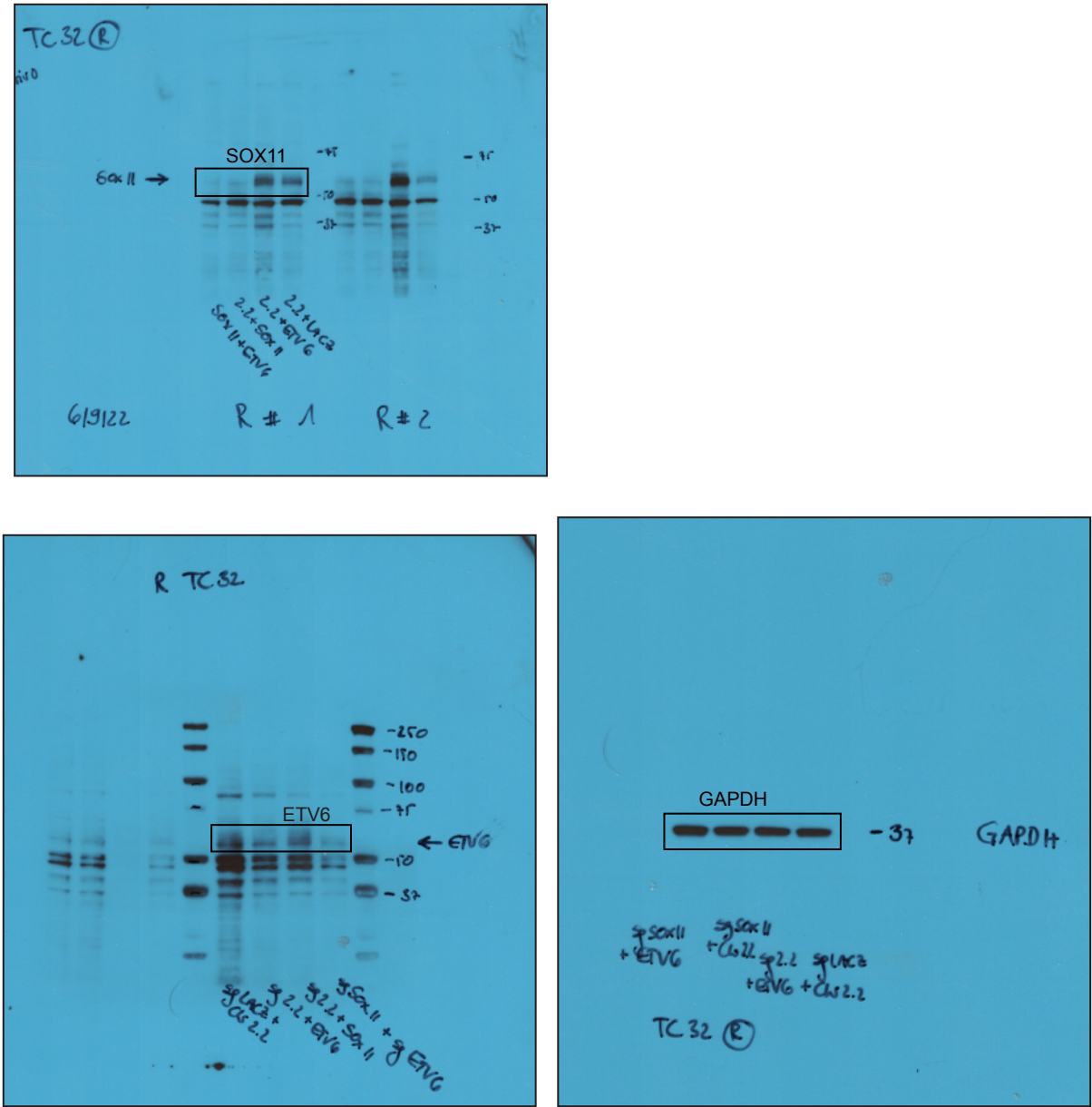

Supplement: Fig. 6 raw western blots — Unprocessed westerns shown in Fig. 6. [file 41556_2022_1059_MOESM14_ESM.pdf]

Extended Data Figure 1f

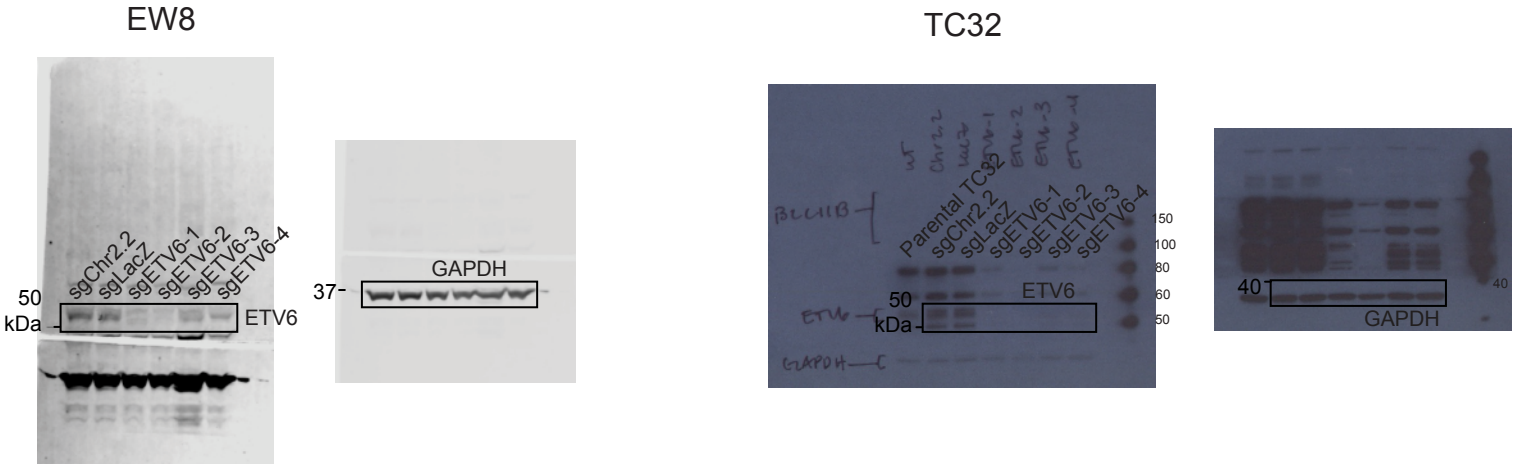

Extended Data Figure 1h

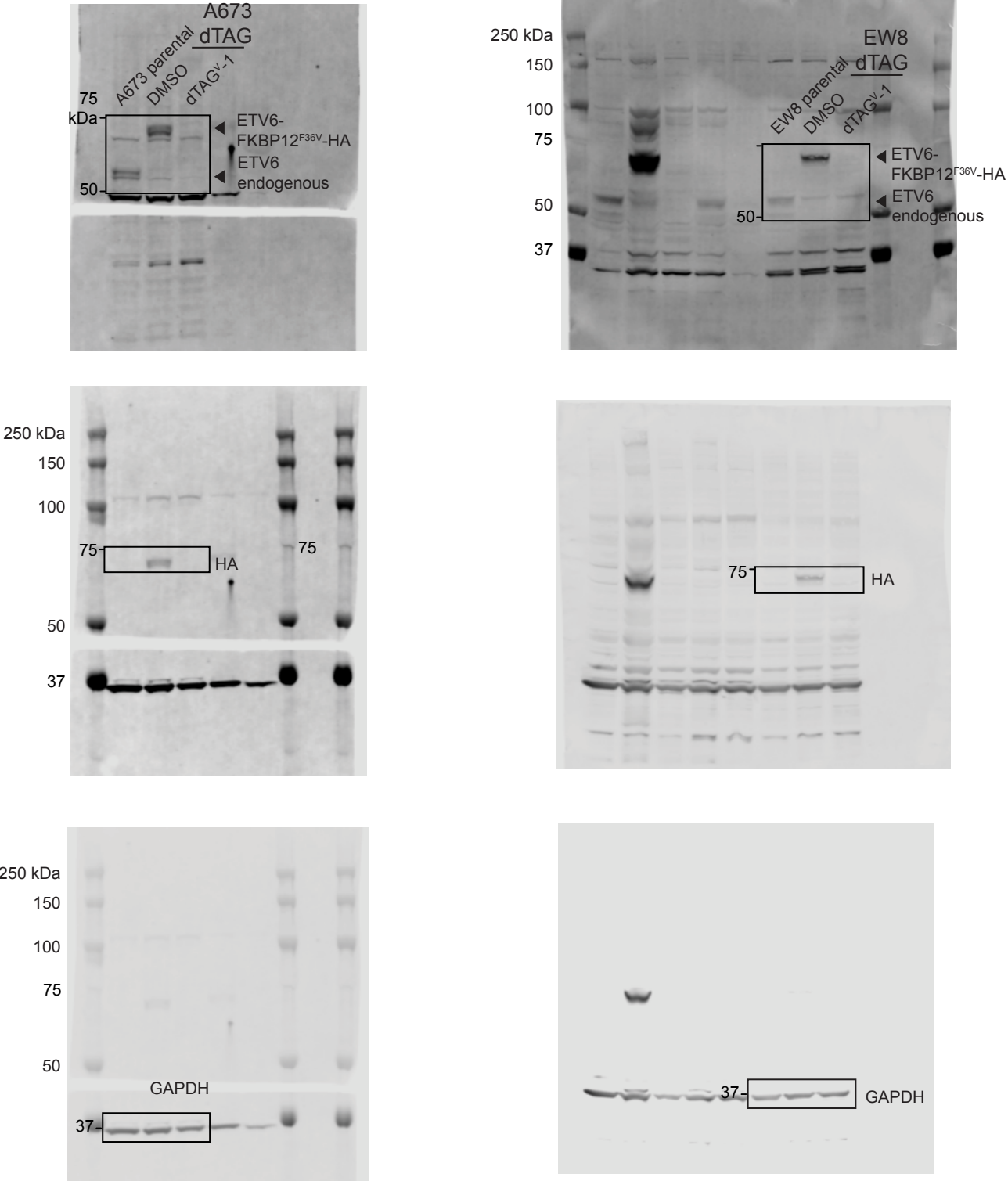

Supplement: Extended Data Fig. 1 — Unprocessed westerns shown in Extended Data Fig. 1. [file 41556_2022_1059_MOESM15_ESM.pdf]

Extended Data Figure 4a

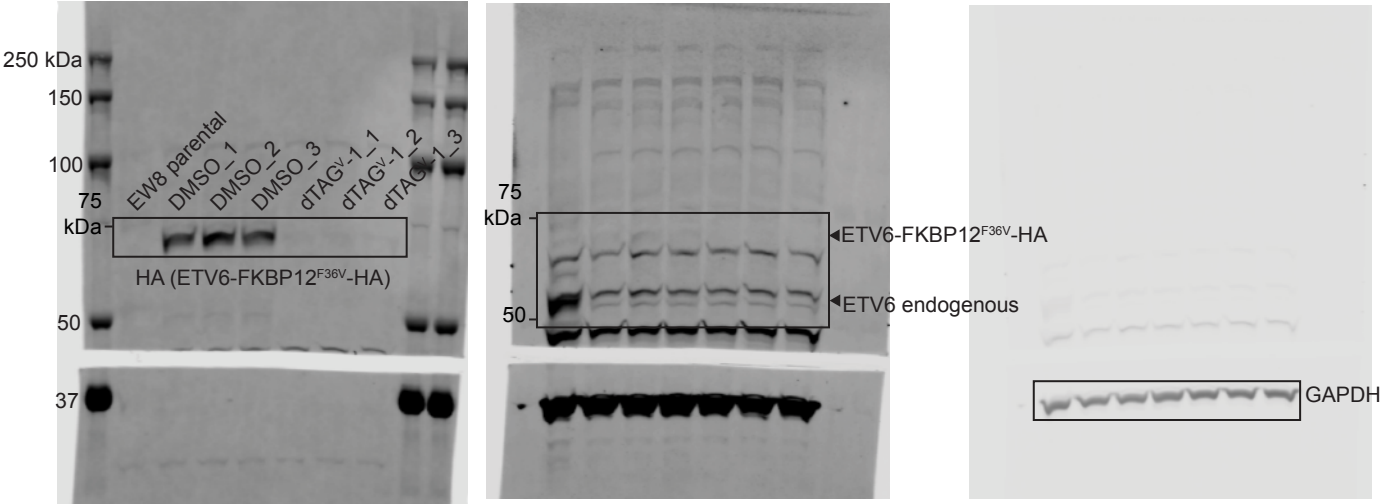

Extended Data Figure 4d

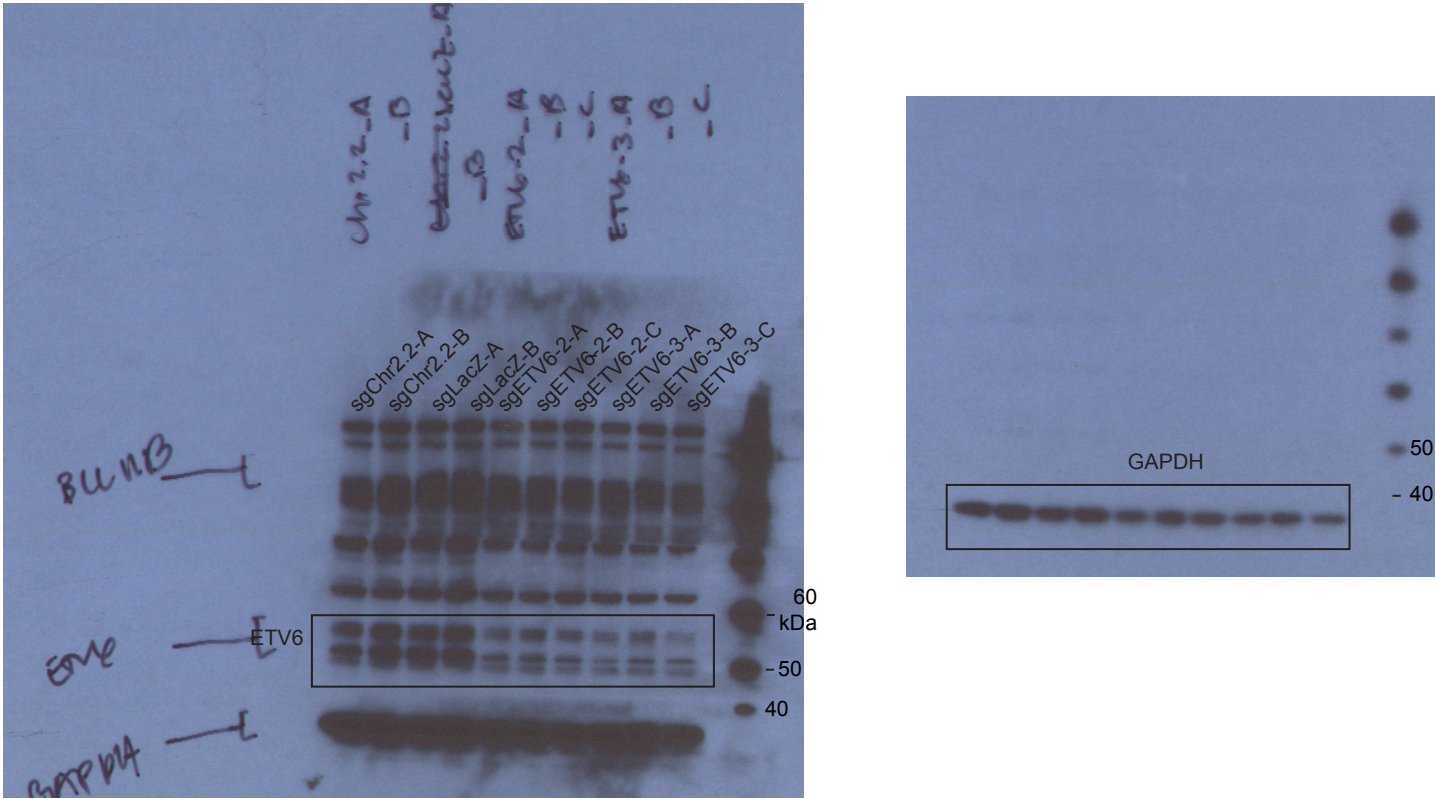

Supplement: Extended Data Fig. 4 — Unprocessed westerns shown in Extended Data Fig. 4. [file 41556_2022_1059_MOESM17_ESM.pdf]

Extended Data Figure 6a

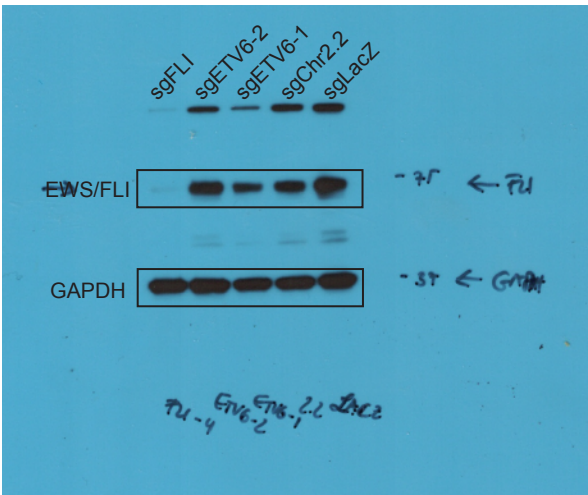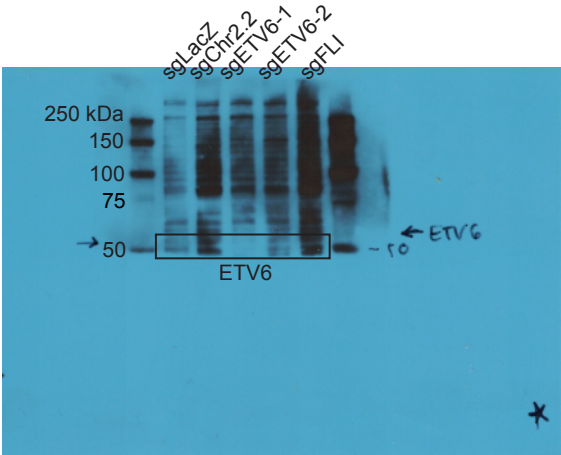

Extended Data Figure 6b

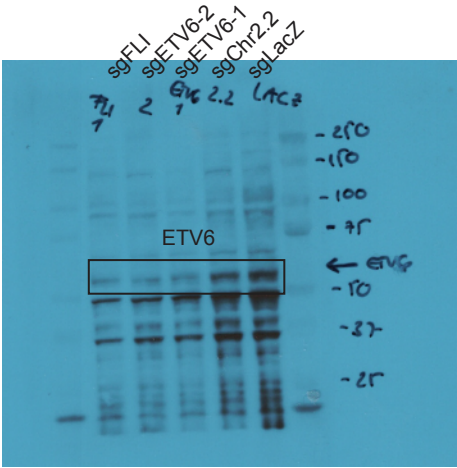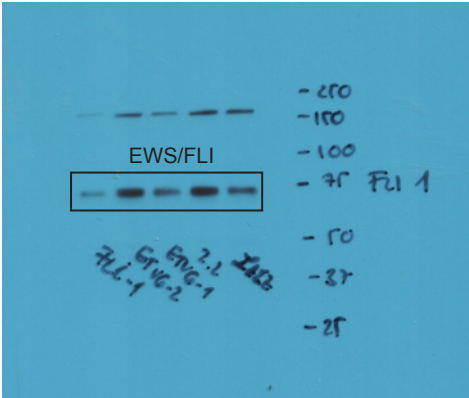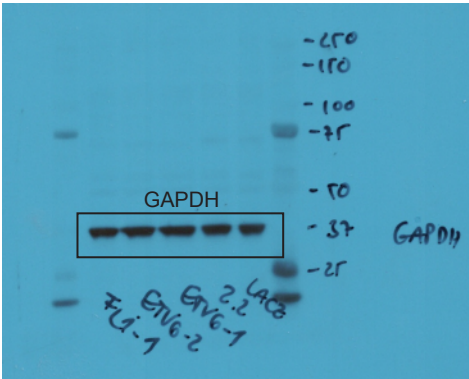

Extended Data Figure 6c

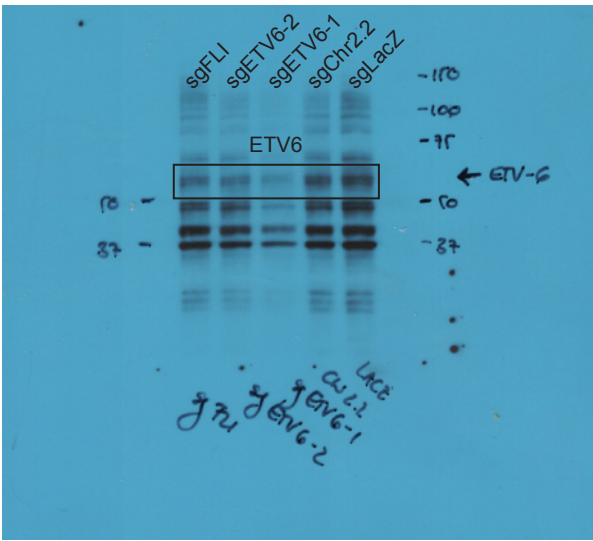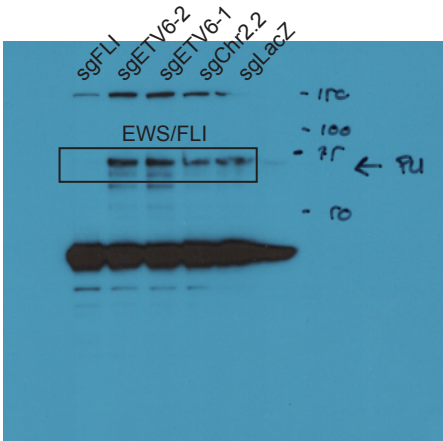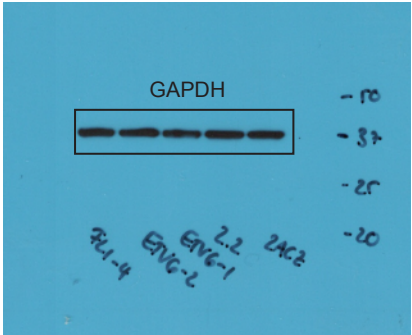

Supplement: Extended Data Fig. 6 — Unprocessed westerns shown in Extended Data Fig. 6. [file 41556_2022_1059_MOESM18_ESM.pdf]
