# Supplementary material for: The ETS transcription factor ETV6 constrains the transcriptional activity of EWS–FLI to promote Ewing sarcoma
Source: Nat Cell Biol. 2023 Jan 19;25(2):285–97. doi: 10.1038/s41556-022-01059-8 (PMC9928584; doi:10.1038/s41556-022-01059-8)

Extended Data Figure 2b

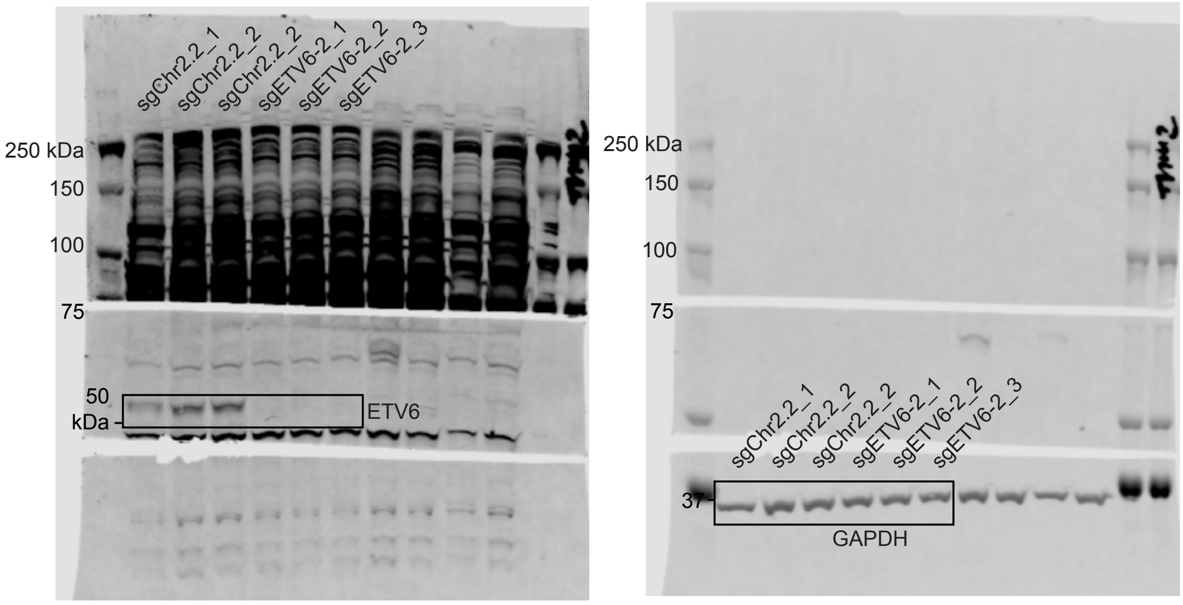

Extended Data Figure 2c

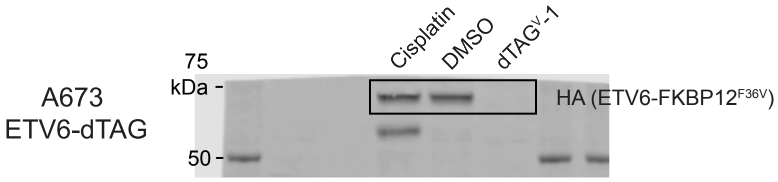

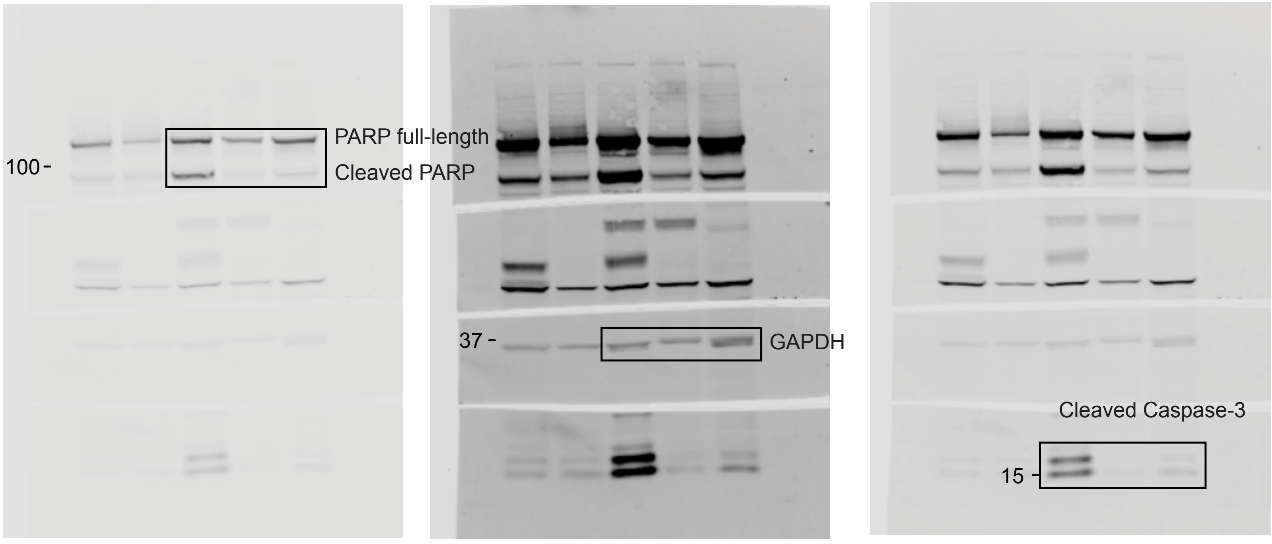

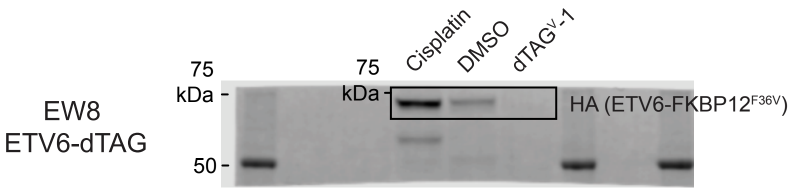

Extended Data Figure 2c, continued

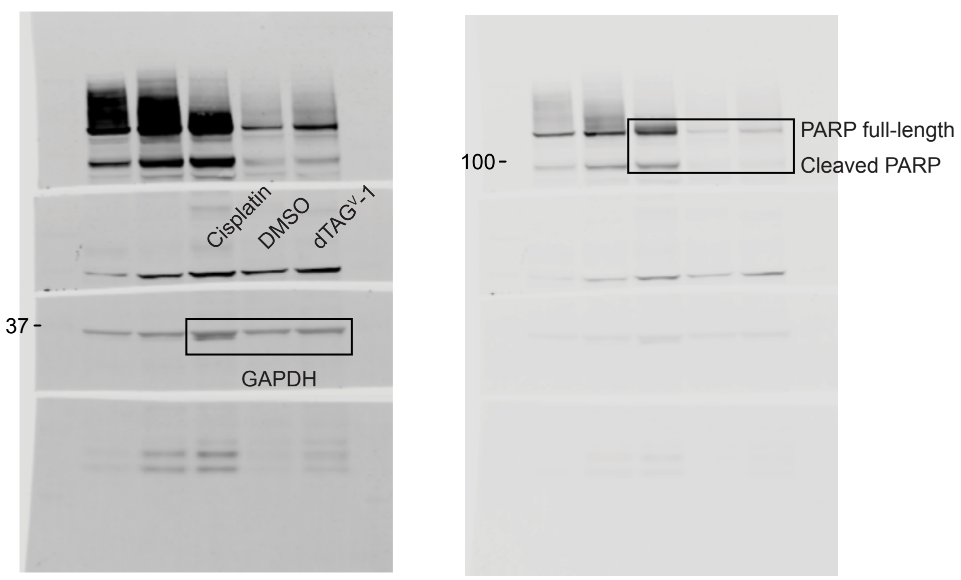

Extended Data Figure 2d

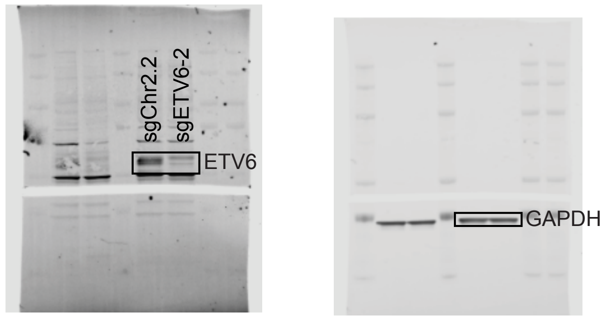

Extended Data Figure 2e

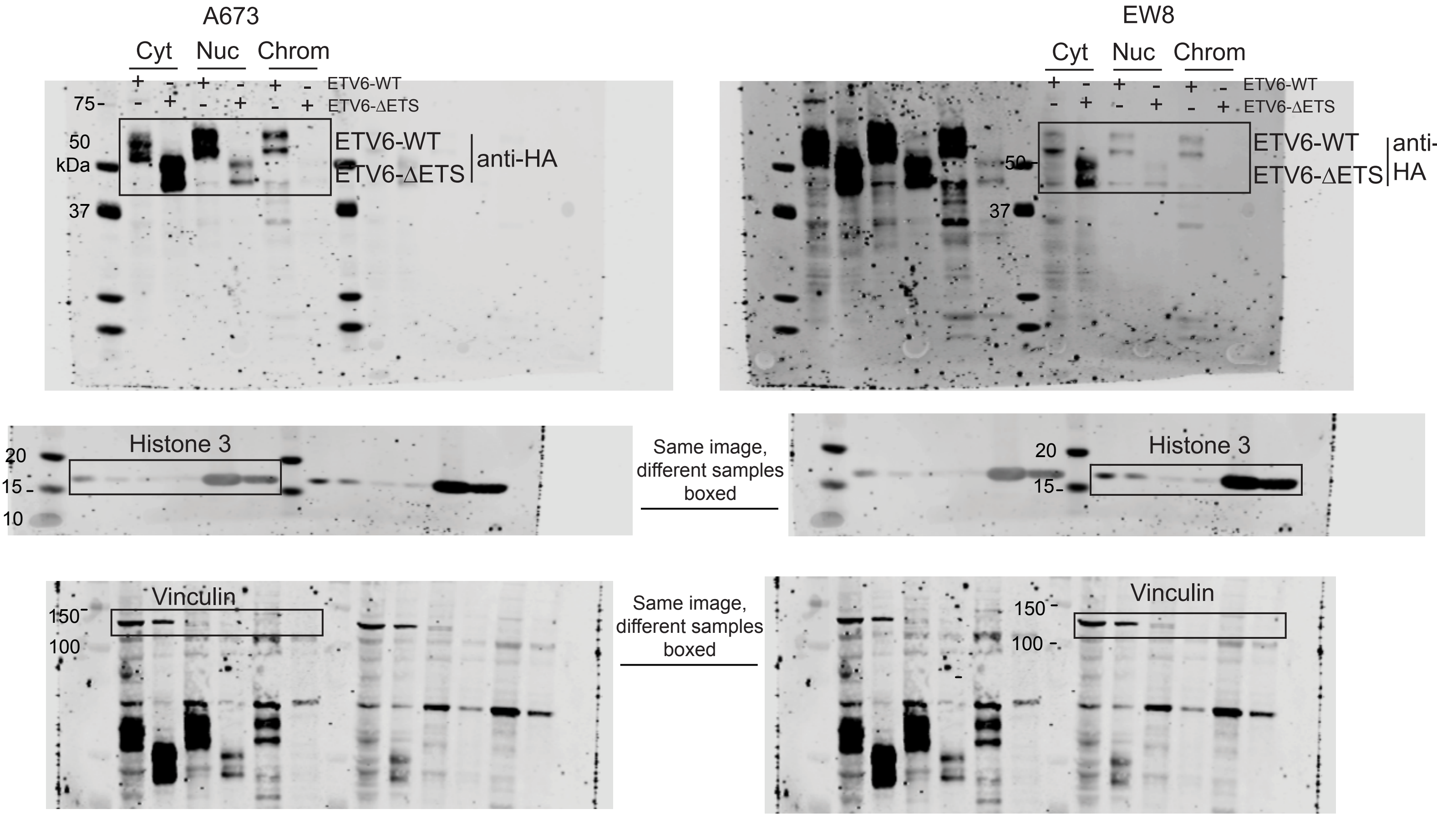

Supplement: Extended Data Fig. 2 — Unprocessed westerns shown in Extended Data Fig. 2. [file 41556_2022_1059_MOESM16_ESM.pdf]
